# Universally Quantitative Band-Selective Pure Shift NMR Spectroscopy

**DOI:** 10.1021/acs.analchem.4c01199

**Published:** 2024-05-30

**Authors:** Howard M. Foster, Mathias Nilsson, Ralph W. Adams, Gareth A. Morris

**Affiliations:** Department of Chemistry, The University of Manchester, Oxford Road, Manchester M13 9PL, U.K.

## Abstract

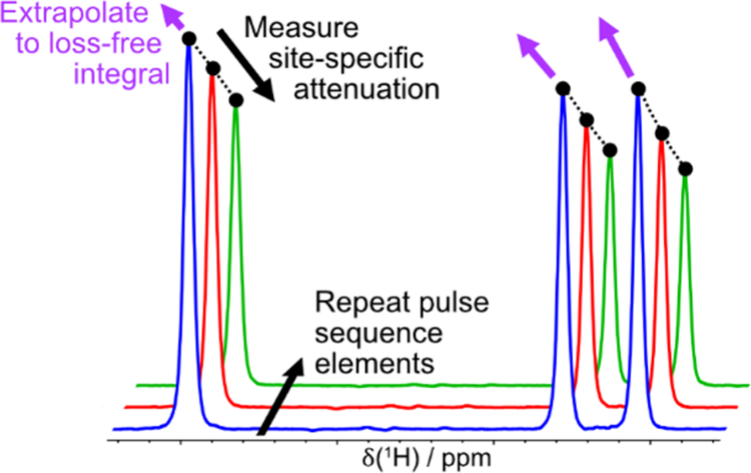

NMR spectroscopy is often described as a quantitative analytical technique. Strictly, only the simple pulse-acquire experiment is universally quantitative, but the poor signal resolution of the ^1^H NMR pulse-acquie experiment frequently complicates quantitative analysis. Pure shift NMR techniques provide higher resolution, by reducing signal overlap, but they are susceptible to a variety of sources of site-dependent signal loss. Here, we introduce a new method that corrects for signal loss from such sources in band-selective pure shift NMR experiments, by performing different numbers of iterations of the same pulse sequence elements before acquisition to allow extrapolation back to the loss-free signal. We apply this method to both interferogram and semi-realtime acquisition modes, obtaining integrals within 1% of those acquired from a pulse-acquire experiment for a three-component mixture.

NMR spectroscopy is capable of being a “universally” quantitative analytical technique in which all signal integrals have the same constant of proportionality with the number of nuclei that generate the signal. This has led to quantitative NMR (qNMR) being performed on a wide variety of analytes, including illicit drugs,^1−3^ pharmaceuticals,^4−6^ natural products,^7−9^ proteins,^10^ metabolites,^11^ and agrochemicals.^12^ However, the ^1^H NMR spectra of complex molecules and mixtures frequently show a high degree of signal overlap due to the limited range of ^1^H chemical shifts and ubiquitous signal multiplicity caused by scalar (*J*) coupling. The inability to clearly distinguish signals hinders the extraction of accurate signal integrals for quantitation. Pure shift (or homonuclear decoupled) NMR techniques are powerful tools for reducing signal overlap, as they yield spectra in which each multiplet is collapsed into a single peak.^13,14^ However, the challenge in applying pure shift NMR techniques to quantitation is that which faces any multiple-pulse NMR experiment: susceptibility to a variety of sources of site-dependent signal loss that stop the experiment being universally quantitative, meaning that quantitative information cannot be obtained by comparing the integrals of different signals within a given spectrum. These sources of error in quantitation include spin relaxation during delays, diffusional attenuation when applying pulsed field gradients (PFGs), losses due to the spatial nonuniformity of radiofrequency (RF) pulses and PFGs, and losses through scalar coupling. Any universally quantitative method must compensate or account for these losses, or ensure that they are uniform for all signals of interest.

Multidimensional experiments have received significant attention in qNMR, as they are better able to resolve signals by dispersing them along additional dimensions. However, as multiple-pulse NMR experiments, they too are susceptible to site-dependent signal losses.^15^ There has been a particular focus within the field of multidimensional qNMR on more quantitative ^1^H–^13^C heteronuclear single-quantum coherence (HSQC) experiments. The quantitative HSQC (Q-HSQC) experiment approximately compensates for the effect of variations in one-bond carbon–proton coupling constants by utilizing variable delays in the insensitive nuclei enhanced by polarization transfer (INEPT) blocks.^16^ The later “quick, quantitative HSQC” (QQ-HSQC) experiment provides a 4-fold reduction in experiment time by employing spatially selective pulses to make different parts of the sample experience the different INEPT delays.^17^ Further development of the Q-HSQC methodology led to the “quantitative, offset-compensated, CPMG-adjusted HSQC” (Q-OCCAHSQC) experiment, which compensates for ^13^C off-resonance effects and for the influence of carbon–proton and proton–proton couplings in the INEPT block.^18^ The most recent variant of the experiment is the “quantitative, equal carbon HSQC” (QEC-HSQC) experiment, which delivers an equal signal response irrespective of the number of protons attached to a carbon.^19^ All of these experiments provide a better degree of quantitativity than the parent experiment, but none reaches the goal of universal quantitativity.

A different approach, which is adapted and extended in this work, may be found in the extrapolated time-zero HSQC (HSQC_0_) experiment, which seeks to measure and correct for, rather than to eliminate, the losses for different signals in the HSQC experiment.^20^ To this end, the core of the HSQC pulse sequence (excluding the initial excitation and evolution periods) is applied 1, 2, ··· *n* times before data acquisition. Provided that the effects of successive applications of the core sequence on signal intensities are strictly multiplicative, the *n* HSQC spectra obtained can then be used to extrapolate the individual signal integrals back to zero attenuation. However, while the initial results reported for the HSQC_0_ method showed considerable improvements, they were still not fully quantitative.

Despite the interest in developing universally quantitative multidimensional NMR experiments,^16−20^ to date, there has been little effort to do the same for pure shift NMR experiments. Pure shift techniques have generally only been applied in qNMR where full quantitativity is not a concern: for example, where signals are expected to have similar relaxation, diffusion, and scalar coupling properties (e.g., diastereomeric ratio determination),^21^ or where calibration spectra of the species of interest with known concentration have been obtained.^22^ Mauve et al. combined developments in quantitative HSQC wit pure shift in their quantitative, perfected, and pure shifted (QUIPU) HSQC experiment, but the method does not compensate for site-dependent differences in relaxational or diffusional attenuation.^23^

There are a variety of ways in which pure shift NMR techniques^13,14^ remove the effect of homonuclear scalar coupling on a spectrum, so that each multiplet is collapsed to a single peak. 2D methods may either decouple the indirect dimension, as in constant-time^24,25^ or time-reversal experiments,^26,27^ or the 45° projection of the 2D spectrum may be decoupled, as in 2D *J*-resolved^28,29^ or anti-z-COSY experiments.^30^

Homonuclear decoupling of the direct dimension may be achieved with *J*-refocusing elements by exploiting the slow evolution of *J*-coupling compared to that of the chemical shift: a pure shift "free induction decay" (FID), or more properly an “interferogram”, is constructed using “chunks” of FID of short duration (typically 5–25 ms), with *J*-evolution being refocused at the midpoint of each chunk. In such experiments, the *J*-refocusing element typically consists of a broadband 180° RF pulse and an active spin refocusing (ASR) element, of which there are several varieties. The purpose of an ASR element is to refocus only the “active” spins (those contributing to the observed signal) while leaving the remaining coupled (“passive”) spins unperturbed. The simplest ASR element is a band-selective 180° RF pulse, which affords a pure shift spectrum for a band of frequencies, provided there is no mutual scalar coupling within the band.^31,32^ Alternative ASR elements that can allow broadband homonuclear decoupling do so at the expense of sensitivity, which, naturally, is detrimental to integral accuracy.^33−35^

Pure shift NMR experiments that employ *J*-refocusing elements may be used in several different acquisition modes. The first to be introduced was the interferogram mode, in which the pure shift FID is constructed from a series of individual data points,^33^ or chunks of multiple data points,^34^ acquired in a pseudo-2D manner. This results in a significantly worse signal-to-noise ratio (SNR) per unit time than a conventional pulse-acquire experiment. An alternative is real-time acquisition, in which FID acquisition is periodically interrupted by *J*-refocusing elements.^36−38^ Although this allows fast acquisition of a pure shift FID, relaxation during the ASR elements causes discontinuities in the FID, degrading both the resolution and cleanliness of the spectrum. A compromise is offered by the semi-realtime mode, in which FID acquisition is interleaved with passive spin inversion elements that leave active spin chemical shift evolution unperturbed.^39^ By performing multiple (at least two) experiments with time-shifted acquisition periods, a full pure shift “FID” can be constructed with the same resolution and spectral purity as in interferogram acquisition but in a much shorter experiment time. One limitation of the semi-realtime mode is that the approximate chemical shifts of the passive spins must be known so they can be targeted for inversion.

The HSQC_0_ experiment utilizes an elegant fundamental premise that has perhaps been underappreciated in the field of qNMR: site-specific attenuation can be corrected for in multiple-pulse experiments so long as the differential attenuation of each signal can be accurately measured. Implicit in this approach is the need to ensure that the effects of the core pulse sequence are strictly multiplicative, which in turn requires rigorous control of coherence transfer pathways (CTPs).

Band-selective pure shift NMR experiments are particularly vulnerable to site-dependent signal losses from spin relaxation and diffusion, both of which are implicitly accounted for in the HSQC_0_ approach. However, there are several challenges in exchanging the HSQC “core” sequence for a pure shift NMR equivalent. One of these is ensuring that the homonuclear scalar coupling evolution is left unaffected by performing multiple iterations of a *J*-refocusing element. A related issue is that *J*-couplings also cause site-dependent signal loss, which must either be minimized during acquisition or corrected for postacquisition. Applying multiple consecutive *J*-refocusing elements introduces greater opportunities for unwanted CTPs to contribute to the signals observed, and this is exacerbated by the requirement to use consistent PFG amplitudes to ensure that each iteration of the core sequence causes the same diffusional attenuation. Independent phase cycling of each selective 180° RF pulse is therefore needed. A further complication is that convection, while not a source of site-dependent signal loss, does not attenuate signal integrals consistently with each iteration, so convection compensation must be implemented to reduce distortions to the integral extrapolations.

Here, we apply the underlying principle of the HSQC_0_ experiment to band-selective pure shift NMR experiments, with the ambitious target of achieving results that are universally quantitative to within 1% (Figure 1). To reflect the more general applicability of this development of the HSQC_0_ approach, we suggest the name extrapolating quantitative integrals by successive iteration (EXQUISITE).

**Figure 1:**
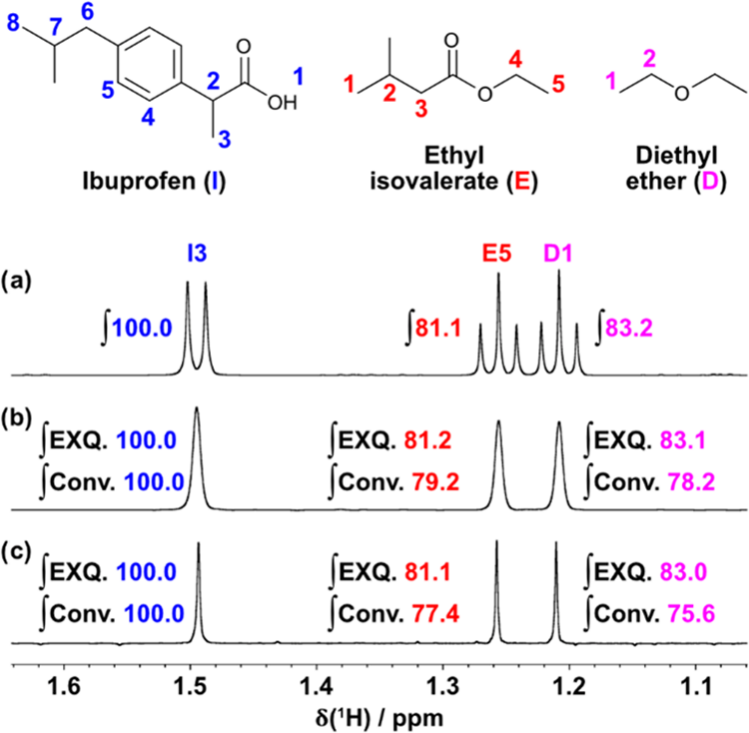
500 MHz ^1^H NMR spectra of a mixture of ibuprofen, diethyl ether, and ethyl isovalerate in CDCl_3_ obtained from (a) a pulse-acquire experiment, and (b, c) the first iterations of EXQUISITE band-selective pure shift NMR experiments, in interferogram and semi-realtime acquisition mode, respectively. Relative integrals are displayed next to each signal. The integrals shown in (b, c) were the mean values obtained from three replicates of a three-iteration EXQUISITE extrapolation (EXQ.) and the conventional pure shift experiment (Conv.). The relative integrals in (a) were obtained from a 700 MHz pulse-acquire experiment in order to reduce the effect of the slight overlap of signals E5 and D1 in (a); only 500 MHz NMR spectra are shown to allow for signal resolution to be compared. Spectra were scaled to show similar maximum intensities; Figure S4 in the Supporting Information shows the signals obtained for the individual EXQUISITE iterations.

## Experimental Section

### NMR Experiments

NMR experiments were performed using a Varian/Agilent VNMRS500 and two Bruker Avance NEO NMR spectrometers, operating at ^1^H resonance frequencies of 499.83, 500.13, and 700.13 MHz, respectively. The two 500 MHz spectrometers were equipped with 5 mm room temperature triple resonance probes with triple axis PFG coils of 67 G cm^–1^ maximum nominal *z*-axis strength in both cases. The 700 MHz spectrometer was equipped with a 5 mm helium-cooled broadband observe probe. Probe temperatures were nominally regulated at 25 °C unless stated otherwise. All experiments were performed with an intertransient delay at least seven times the maximum *T*_1_ value of the signal(s) of interest (to give >99.9% equilibrium magnetization recovery). The hard 90° RF pulse durations were calibrated for each set of NMR experiments. EXQUISITE band-selective pure shift NMR experiments, in both interferogram and semi-realtime acquisition modes, were carried out to assess the quantitative performance of the method. The durations and power levels of the selective shaped 180° RF pulses in these experiments were calibrated automatically using Pbox (Varian/Agilent) or WaveMaker (Bruker). A quantitative 700 MHz ^1^H pulse-acquire experiment was performed to accurately determine the composition of a mixture containing ibuprofen, diethyl ether, and ethyl isovalerate. *T*_1_ time constants were measured using inversion recovery experiments, which were processed using the General NMR Analysis Toolbox (GNAT), MATLAB version 1.2.^40^ All other spectra were processed using either VnmrJ 4.2 or TopSpin 4.1.3. Further experimental and data processing details, macros used for data processing and experimental setup, and pulse sequence codes may be found in the Supporting Information.

### NMR Simulations

Simulations of NMR pulse sequences were performed using the Spinach^41^ package (version 2.4.5157) in MATLAB (version R2021b), and Mathematica (version 12.3). TopSpin (version 4.1.3) was used to process the simulated Spinach FIDs. Other numerical simulations were performed in MATLAB and Mathematica. All files used to perform the simulations, along with all experimental data, macros, and pulse sequence codes, are available at DOI 10.48420/25324786.

### NMR Sample Preparation

The doped water sample was a Bruker standard sample in a thin-walled 5 mm NMR tube containing 0.1 mg mL^–1^ GdCl_3_, 0.1% DSS, and 1% H_2_O in D_2_O. All other samples were prepared in thin-walled 5 mm NMR tubes with chemicals purchased from commercial suppliers and used without further purification. The components of the ibuprofen, diethyl ether, and ethyl isovalerate mixture had concentrations of 154, 64, and 125 mM, respectively, in CDCl_3_. The doped ethanol sample consisted of ethanol (295 mM) and chromium acetylacetonate (3 mM) in CDCl_3_.

## Method

Here, we discuss the principles behind the EXQUISITE method as well as its practical implementation in band-selective pure shift NMR.

### Principles

**Figure 2:**
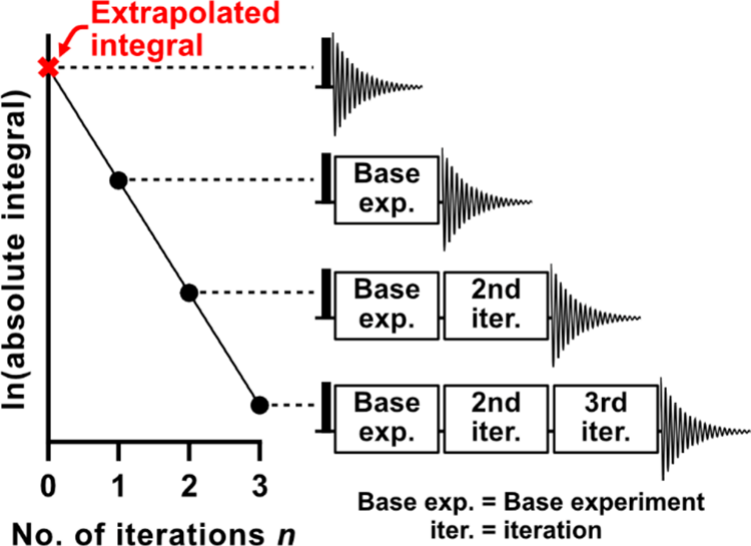
Schematic of the EXQUISITE method showing successive iteration of pulse sequence elements leading to an attenuated signal. The plot in the figure is the natural logarithm of the signal integral versus the number of iterations *n*.

If a given pulse sequence element attenuates a given signal integral by a factor *f*_element_, then provided that each iteration of that element acts independently, repeating the element *n* times before detection results in an integral attenuated by a factor (*f*_element_)^*n*^. The integrals from experiments with different *n* values may then be used to extrapolate the integral to zero attenuation. This is illustrated in the semilog plot of Figure 2; strictly, exponential extrapolation of the integrals is preferable to linear extrapolation of their logarithms, but for small attenuations the difference is negligible. Although each iteration contains the same basic elements, they are not necessarily identical, as they may require different delay durations, RF pulse phases, and/or PFG polarities if consistent attenuation is to be achieved.

The factor *f*_element_ can be divided into individual factors for the primary sources of signal loss during the application of a pulse sequence element. Spin relaxation during a time period *t* may be approximated as reducing a given signal integral by a factor
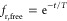
1where *T* is the longitudinal or transverse spin relaxation time constant, depending on whether the magnetization required is longitudinal or transverse during the delay, for a given spin.^42^ Relaxational attenuation caused by an on-resonance selective 180° refocusing pulse has been shown to be well approximated by a biexponential decay, attenuating a given signal integral by a factor
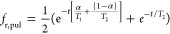
2where α is a pulse shape-specific factor, and *T*_1_ and *T*_2_ are the longitudinal and transverse relaxation time constants, respectively.^43^ The application of an identical pair of rectangular-shaped PFGs on either side of a 180° RF pulse causes diffusional attenuation of a given signal integral according to the Stejskal-Tanner equation by a factor
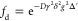
3where *D* is the translational diffusion coefficient of the molecule to which the signal belongs, γ is the gyromagnetic ratio, δ is the PFG duration, *g* is the PFG strength, and Δ′ is the corrected time for which diffusion causes signal attenuation.^44^ The convectional attenuation under the same circumstances can be approximated by a factor
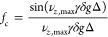
4where *ν*_*z*,max_ is the maximum convectional flow velocity parallel to the *z*-axis, and Δ is the time between the midpoints of the two PFGs.^45^ An additional attenuation factor, *f*_x_ may be defined to account for any signal loss due to imperfections in RF pulses, PFGs, and the applied magnetic field. If these phenomena all act independently, the total signal attenuation factor for a given pulse sequence element is
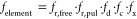
5

Provided each pulse sequence element acts independently, the overall signal attenuation factor for a given signal integral after applying *n* iterations of a collection of *k* individual pulse sequence elements is
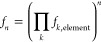
6

However, elements which cause convectional attenuation do not act independently, as convection is not a random but a coherent process. Equation 6 is therefore more appropriately formulated as
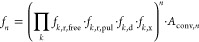
7where *A*_conv, *n*_ is the attenuation of signal integrals due to convection, which depends on the sample, the experimental conditions, and the number of iterations performed, but is not site-specific. This means that within a single experiment, all the extrapolated signal integrals are overestimated to the same extent due to convectional attenuation. Still, the additional attenuation caused by convection is undesirable as it results in poorer SNR, it may hide systematic distortions to the expected exponential decay of signal integral vs *n*, and it becomes a source of quantitation error when comparing the extrapolated integrals from experiments in which convection differs. Accordingly, our implementation of EXQUISITE band-selective pure shift NMR employs convection compensation.

One source of signal loss that cannot be corrected using the EXQUISITE method for pure shift NMR experiments is *J*-modulation of the resultant interferogram. Each chunk has the effects of scalar coupling refocused at its midpoint, but *J*-evolution causes its amplitude to decrease slightly toward the edges. When the chunks are assembled into an interferogram and Fourier transformed, the result is that some of the intensity of the desired pure shift peak is transferred to a series of “chunking sidebands”, spaced at integer multiples of the inverse of the chunk duration, either side of the centerband pure shift signal. The integral of the latter is determined by the average signal amplitude over the whole chunk rather than the amplitude of the first point of the FID. For a system containing *S* spins, the attenuation of the centerband peak of spin *k* is, to second order,
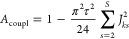
8

The derivation of eq 8 may be found in the Supporting Information (Section S6). Values of *A*_coupl_ predicted by eq 8 closely match those obtained by numerical simulation of “chunked” pure shift FIDs for a relatively wide multiplet in a four-spin system (*J* = 17.2, 10.3, and 7.6 Hz) for chunk durations ≤20 ms (Figure S6).

The dependence of the signal loss on the square of the chunk duration highlights the need either to employ short chunk durations (≤10 ms), or to apply the correction factor of eq 8 if all coupling constants are known. Ultraselective methods such as GEMSTONE (gradient-enhanced multiplet-selective targeted-observation NMR experiment),^46^ or 2D *J*-resolved techniques,^28,29^ may be used to determine coupling constants where there is severe signal overlap. Applying a correction factor permits the use of longer chunk durations, more typical of routine pure shift NMR experiments (ca. 20 ms), resulting in a shorter experiment time. Additionally, it affords a greater choice of chunk durations and thus sideband spacings, allowing a sideband spacing to be chosen that avoids chunking sidebands overlapping with nearby signals. It should be noted that although the sideband averaging by periodic phase incrementation of residual *J*-evolution (SAPPHIRE) method can be used to suppress chunking sidebands, and varying the chunk duration during time averaging can disperse them, neither method restores the lost centerband signal intensity.^47,48^

A further small complication caused by scalar coupling is that the rate of relaxation of transverse magnetization in coupled spin systems is greater for antiphase multiplet components than for in-phase, because of the extra contribution provided by the spin-lattice relaxation of passive spins.^49^ Loosely described by the term “scalar relaxation of the third kind”, this has two different types of effect in pure shift experiments. First, it causes weak sidebands to appear at ±*J*/2 either side of the centerband peak. These should be included in the peak integral regions. Second, it causes a small extra contribution to the relaxation losses during iterations, which can be included in the relaxation term *f*_k,r,free_ since the repeated *J*-refocusing should ensure that the effect is multiplicative.

Consideration of all of these sources of site-/sample-dependent signal loss leads to an overall signal attenuation factor for EXQUISITE band-selective pure shift NMR experiments of
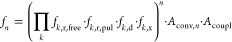
9

Fitting the signal integral (or natural logarithm of the signal integral) as a function of the number of iterations leads to a signal attenuation factor per iteration *f*_iter_.

### Practical Implementation

**Figure 3:**
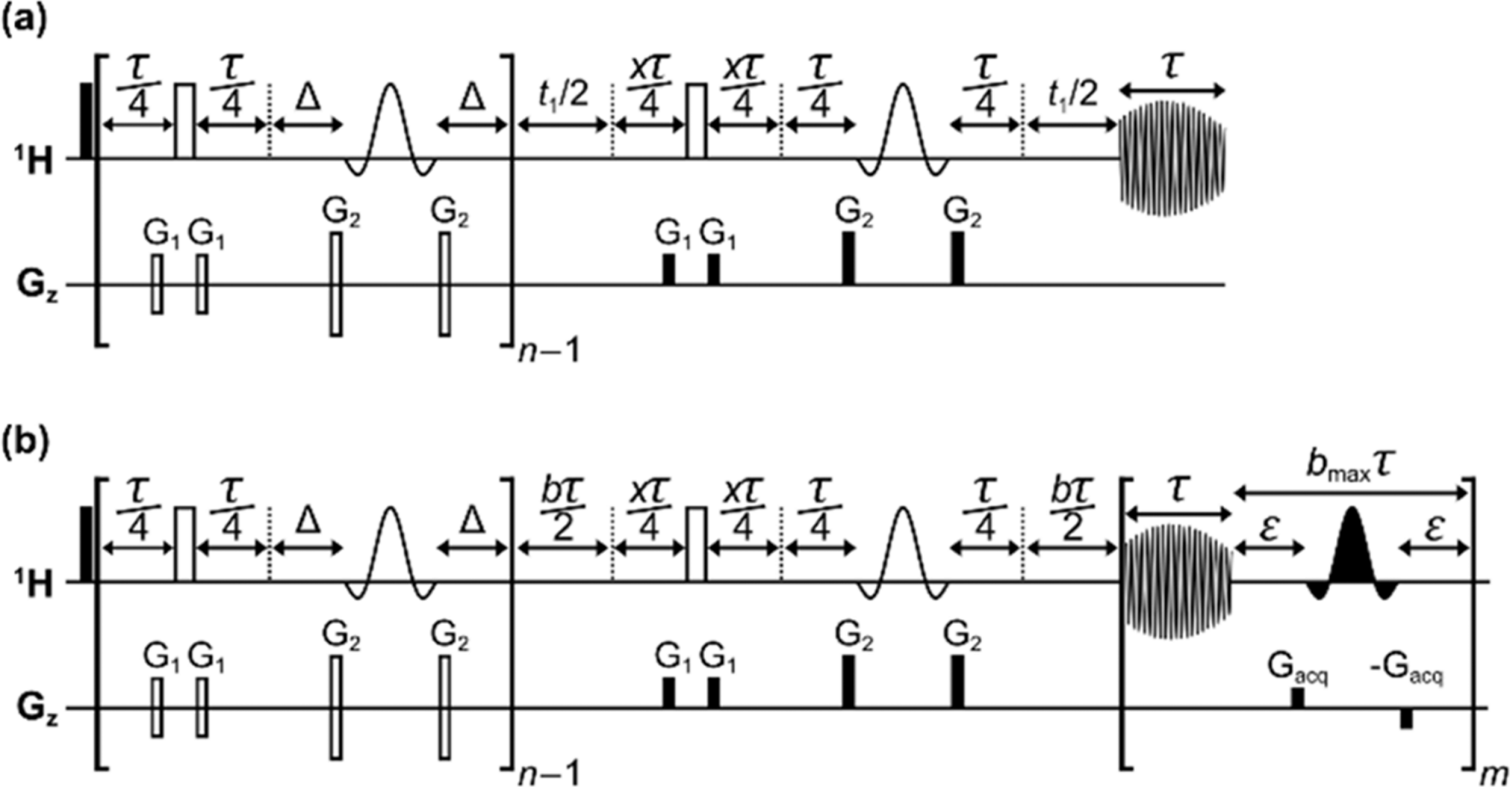
Schematic pulse sequences for quantitative band-selective pure shift NMR with the EXQUISITE method in interferogram (a) and semi-realtime (b) acquisition modes. Filled and open rectangles on the ^1^H line represent broadband 90 and 180° RF pulses, respectively. Filled and open “sinc” (sin[*x*]/*x*) shapes represent selective 180° RF pulses applied to the “passive” and “active” spins, respectively. Rectangles on the *G*_*z*_ line represent PFGs, with open rectangles representing the PFGs that alternate in polarity between successive iterations, for convection compensation. The number of EXQUISITE iterations is *n*. For odd *n*, *x* = 1; for even *n*, *x* = 2. See the Supporting Information (Section S1) for a detailed explanation of pulse sequence timings.

An implementation of the EXQUISITE method with band-selective pure shift NMR experiments must fulfill two criteria: signal integral attenuation should be consistent for each iteration, and all spectra obtained must be pure shift NMR spectra. Satisfying both conditions requires careful management of delays, PFG amplitudes and polarities, and RF pulse phases in such pulse sequences (Figure 3).

As noted earlier, there is an interaction between the different pulse sequence elements that cause convectional attenuation, which prevents convection from being accounted for in the EXQUISITE method. Although all signal integrals within the same spectrum are affected identically, this additional source of attenuation should ideally be minimized. In the pulse sequences of Figure 3, the effects of convection are refocused in alternate iterations, so that for even *n*, convection is completely compensated (*A*_conv, *n*_ = 1), and for odd *n*, convectional attenuation is limited to that arising from a single iteration (*A*_conv, *n*_ = *A*_conv, 1_). This is achieved by alternating PFG polarities for successive iterations while keeping PFG amplitudes constant. See the Supporting Information (Section S7) for a comparison of experiments performed with alternating and consistent PFG polarities. Further measures, as recommended in the literature, may be taken to reduce sample convection.^50^

The phases of chunking sidebands in pure shift NMR spectra are determined by the time point at which *J*-evolution is refocused during the chunk. Refocusing *J*-evolution at the beginning of each chunk results in pure dispersion mode sidebands, and refocusing at the midpoint gives the more desirable pure negative absorption mode chunking sidebands. In conventional pure shift NMR experiments, *J*-evolution is refocused at the midpoint of each chunk by the inclusion of a pair of τ/4 delays flanking the hard 180° RF pulse. If a second, identical *J*-refocusing element were to be applied, this would result in zero net *J*-evolution, and thus dispersion mode chunking sidebands, as the senses of *J*-evolution in the two sets of τ/4 delays are opposed. This effect is counteracted in the EXQUISITE pure shift NMR sequences of Figure 3 by doubling the duration of one set of these delays; relaxational attenuation is kept constant by adjusting the Δdelays to ensure that the total time between the initial 90° excitation pulse and the beginning of acquisition (excluding the *t*_1_ evolution periods) is an integer multiple of the duration of a single iteration.

CTP enforcement in NMR pulse sequences may be achieved by phase cycling, the application of PFGs, or (ideally) a combination of both. An important limitation of the EXQUISITE method is the requirement for consistent PFG amplitudes in order to effect consistent diffusional attenuation, meaning that CTP enforcement is more reliant on phase cycling. Systematically changing both the amplitude of a pair of PFGs and the interval between them would provide consistent diffusional attenuation but sacrifice convection compensation, and so was not used here. With consistent PFG amplitudes, certain stimulated echo pathways lead to measurable signal in the absence of sufficient independent phase cycling of the 180° RF pulses (see Section S8 of the Supporting Information for specific unwanted CTPs that are suppressed by the PFGs and by phase cycling). The selective 180° RF pulses are the most vulnerable to imperfections, so these are phase cycled in an alternating two-step (*x*, *y*), four-step (*x*, *y*, *–x*, *–y*) pattern in successive iterations. This results in the minimum number of phase cycling steps depending on the number of iterations performed: 2, 8, 16, 64, and 128 steps are needed for one–five iterations, respectively.

## Results and Discussion

To investigate whether the principles of the EXQUISITE band-selective pure shift NMR method would stand up to experimental scrutiny, the method was applied to two samples. One was a singlet model system (a doped water sample) so that the method could be assessed without the complication of differential signal loss due to *J*-modulation. Although informative, applying a pure shift NMR technique to a singlet signal is not of practical use, so we further investigated the method by targeting one multiplet signal from each component in a mixture of ibuprofen, diethyl ether, and ethyl isovalerate. Additionally, we assessed by Monte Carlo modeling how the quantitation uncertainty is influenced by *f*_iter_, SNR, and *n*.

### Water Model System

Four sets of EXQUISITE band-selective interferogram pure shift NMR experiments, each with different experimental parameters, were performed on a doped water sample. By varying the amplitudes of PFGs and the inclusion of additional delays in the sequence, the water signal could be attenuated differently by relaxation, diffusion, and convection in each set of experiments A–D. Experiments C and D used higher PFG amplitudes G_2_ than experiments A and B (29.4 vs 20.8 G cm^–1^), so were more susceptible to diffusion and convection losses. Experiments B and D included an additional 40 ms of delays per iteration, making them more susceptible to relaxation losses. Full experimental details are given in the Supporting Information (Section S2.3).

All experimental data for this sample were acquired on a Varian/Agilent VNMRS500 NMR spectrometer. The quantitativity of the method was assessed by considering the deviation of each of the four extrapolated integrals from their mean values in each of 13 replicates. As the same resonance was measured in each experiment, the extrapolated integrals should be identical. For simplicity, the EXQUISITE extrapolation was performed by fitting the natural logarithm of signal integral vs *n* (equivalent results from exponential fitting may be found in Section S4.1 of the Supporting Information). Table 1 shows the mean integral deviation of the “base” experiment (i.e., the conventional band-selective pure shift NMR experiment), and the mean deviations of the integrals derived from extrapolation using two and three iterations for the set of four experiments, as well as the mean values of *f*_iter_ for each set. These data show that there is a dramatic improvement in the quantitative performance of the band-selective pure shift NMR experiment when the EXQUISITE method is used: the conventional experiment has integral deviations with magnitudes of up to ca. 13%, but these fall to less than 0.5% when using a two- or three-iteration extrapolation.

**Table 1:** Mean Deviations of the Integrals for the “Base” Experiment and the Integrals Extrapolated from Two and Three Iterations for the Water Signal in Four EXQUISITE Band-Selective Pure Shift NMR Experiments A–D with Different Experimental Parameters

exp.	*f*_iter_	no. of iterations	integral deviation/%
A		base	13.36 (±0.03)^a^
	0.66	2	–0.10 (±0.06)
	0.66	3	0.00 (±0.06)
B		base	–6.64 (±0.03)
	0.55	2	–0.31 (±0.06)
	0.55	3	–0.17 (±0.06)
C		base	6.00 (±0.03)
	0.62	2	0.33 (±0.05)
	0.62	3	0.17 (±0.04)
D		base	–12.72 (±0.04)
	0.51	2	0.09 (±0.08)
	0.51	3	–0.01 (±0.08)

a The value quoted in brackets is 1.96 times the standard error of the mean.

### Ibuprofen, Diethyl Ether, and Ethyl Isovalerate (IDE) Mixture

The quantitative performance of the EXQUISITE method was further investigated with selected multiplet signals in a mixture of ibuprofen (I), diethyl ether (D), and ethyl isovalerate (E) in both semi-realtime and interferogram mode band-selective pure shift NMR experiments. Figure 1a shows the three selected signals, one from each component of the mixture, in a 500 MHz ^1^H pulse-acquisition NMR spectrum. Figure 1b,c are spectra obtained from the first iterations of EXQUISITE band-selective pure shift NMR experiments in interferogram and semi-realtime acquisition mode, respectively. The pure shift “FIDs” assembled from the interferogram and semi-realtime mode data consisted of 4000 and 32 640 complex points, respectively, resulting in significantly broader signals in Figure 1b, a reflection of the significantly greater cost in experiment duration of the interferogram mode of acquisition. The interferogram and semi-realtime mode experiments were performed on a 500 MHz Bruker Avance NEO and a 500 MHz Varian/Agilent VNMRS500 NMR spectrometer, respectively; the probe temperature was nominally regulated to 20 °C for the latter experiments due to the greater susceptibility of the VNMRS500 probe to convection. Further experimental and data processing details are given in the Supporting Information (Section S2.5).

Due to the slight overlap between signals E5 and D1 in the 500 MHz ^1^H pulse-acquire spectrum, relative integrals were instead determined from a 700 MHz ^1^H pulse-acquire spectrum. The composition of the IDE mixture was determined to be 44.9% ibuprofen, 18.7% diethyl ether, and 36.4% ethyl isovalerate using signals I3, D1, and E5. The mixture composition was independently estimated using these three signals in EXQUISITE pure shift NMR experiments, using either the relative integrals in the base experiment or the relative extrapolated integrals from semilog fitting of two and three iterations. The mean deviations between the pure shift and pulse-acquire composition values are shown in Table 2, for three replicates of the pure shift experiments. Table 2 contains composition deviations with and without a correction, using eq 8, for the different degrees of *J*-modulation for the three signals. In this case, due to the relatively short chunk durations used (τ = 12.5 and 16 ms for the interferogram and semi-realtime mode experiments, respectively) and the similar multiplet structure, this correction makes only a minor change to the composition deviation obtained. The quantitative accuracy of the EXQUISITE pure shift experiments is an order of magnitude better than that of the conventional pure shift experiments: the relative integral deviations obtained using either a two- or three-iteration extrapolation and a correction for *J*-modulation cover a range of less than 0.15%, whereas the range for the conventional experiments exceeds 1.5%. The relative integral deviations for each signal in each replicate may be found in Table S3 in the Supporting Information.

**Table 2:** Mean Mixture Composition Deviations Determined from the Integrals of Signals H3 of Ibuprofen (I3), H5 of Ethyl Isovalerate (E5), and H1 of Diethyl Ether (D1) in the IDE Mixture, for the “Base” Experiment and Extrapolated from Two and Three Iterations, Using Three Replicates of EXQUISITE Band-Selective Pure Shift NMR Experiments

		mixture composition deviation without correction for *J*-modulation/%			mixture composition deviation with correction for *J*-modulation/%		
acquisition mode	no. of iterations	I3	E5	D1	I3	E5	D1
interferogram	base	0.89 (±0.03)^a^	–0.12 (±0.02)	–0.78 (±0.02)	0.82 (±0.03)	–0.06 (±0.02)	–0.76 (±0.02)
	2	0.12 (±0.05)	–0.08 (±0.02)	–0.03 (±0.05)	0.04 (±0.05)	–0.03 (±0.02)	–0.01 (±0.05)
	3	0.06 (±0.04)	–0.01 (±0.02)	–0.05 (±0.04)	–0.02 (±0.04)	0.05 (±0.02)	–0.03 (±0.04)
semi-realtime	base	1.56 (±0.01)	–0.45 (±0.01)	–1.11 (±0.00)	1.43 (±0.01)	–0.36 (±0.01)	–1.07 (±0.00)
	2	0.21 (±0.02)	–0.12 (±0.04)	–0.09 (±0.02)	0.08 (±0.02)	–0.03 (±0.04)	–0.05 (±0.02)
	3	0.14 (±0.02)	–0.06 (±0.01)	–0.08 (±0.01)	0.01 (±0.02)	0.03 (±0.00)	–0.04 (±0.02)

a The value quoted in brackets is 1.96 times the standard error of the mean.

An alternative metric for judging quantitative accuracy is to consider the relative deviation in component concentration derived from the pure shift integrals and the pulse-acquire integrals. With this treatment of the data, the concentration deviations for the three signals cover a range less than 0.6% across all replicates when using either a two- or three-iteration extrapolation and a correction for *J*-modulation. For the conventional pure shift experiment, the concentration deviations cover a range of >6% across all replicates. The concentration deviations for each signal in each replicate may be found in Table S4.

### Limitations

The data in Table 2 show that the quantitative accuracy of the method is similar whether using interferogram or semi-realtime acquisition mode. As the total experiment time of an EXQUISITE experiment scales with the number of iterations performed and qNMR experiments rely on relatively long recovery delays, the time savings possible with semi-realtime acquisition, typically of an order of magnitude, are particularly attractive. This advantage is compounded by the fact that the achievable signal resolution is not limited by the total experiment time, as it is for the interferogram mode. Despite these benefits, semi-realtime acquisition is not unequivocally preferable, or even practically feasible, in all cases.

Spectrometer instability results in additional chunking artifacts in semi-realtime mode spectra, that occur at submultiples of 1/τ and can complicate quantitative analysis. In interferogram mode, spectrometer instability only causes a pseudorandom perturbation of the usual chunking sidebands at integer multiples of 1/τ. Furthermore, the semi-realtime mode relies not only on being able to selectively target the active spins with minimum excitation of the passive spins, a requirement common to both acquisition modes, but also requires the reverse: that inversion of the passive spins leave the active spins unaffected. In practice, the application of passive spin inversion pulses between chunks of data acquisition can introduce frequency-dependent phase shifts in the resultant spectrum due to the Bloch-Siegert effect.^51^ These problems are most serious when the active spins cover a frequency range that is not small compared to the frequency separation between the active and passive spin regions. Although these factors limit the range of analytes that are compatible with the semi-realtime mode for quantitative analysis, its ability to greatly improve SNR per unit time should be exploited where possible.

### SNR Analysis

As the EXQUISITE method relies on the measurement of signal attenuation, the SNR necessarily decreases as more iterations are performed prior to acquisition. A lower SNR results in greater uncertainty in signal integrals, imposing a limit on the number of iterations *n* that should be used in the extrapolation. However, as *n* increases, the fitting procedure is less susceptible to deviations due to the uncertainties in individual data points. These two opposing effects suggest that there is an optimal number of iterations to perform. Monte Carlo modeling (10 000 replicates) of the EXQUISITE method was performed to find this optimal value for a range of SNRs and values of *f*_iter_, initially assuming no constraint on experiment time. Signal integral attenuation was simulated by scaling a Gaussian-shaped singlet signal with a range of initial signal amplitudes by a range of (*f*_iter_)^*n*^ values, where *n* ranged from one to five. Gaussian noise was superimposed upon this signal, which was then integrated over a region five times its full width at half-maximum. Extrapolation to the loss-free integral was performed by semilog fitting using two to five iterations (*n* = 2, 3, 4, 5) for the 10 000 replicates of each combination of *f*_iter_ and initial signal amplitude (i.e., initial SNR). The quantitation error was calculated as 2√2σ_rel_, where σ_rel_ is the relative standard deviation of the extrapolated signal integral and the √2 factor accounts for comparing the integrals of two such signals.

**Figure 4:**
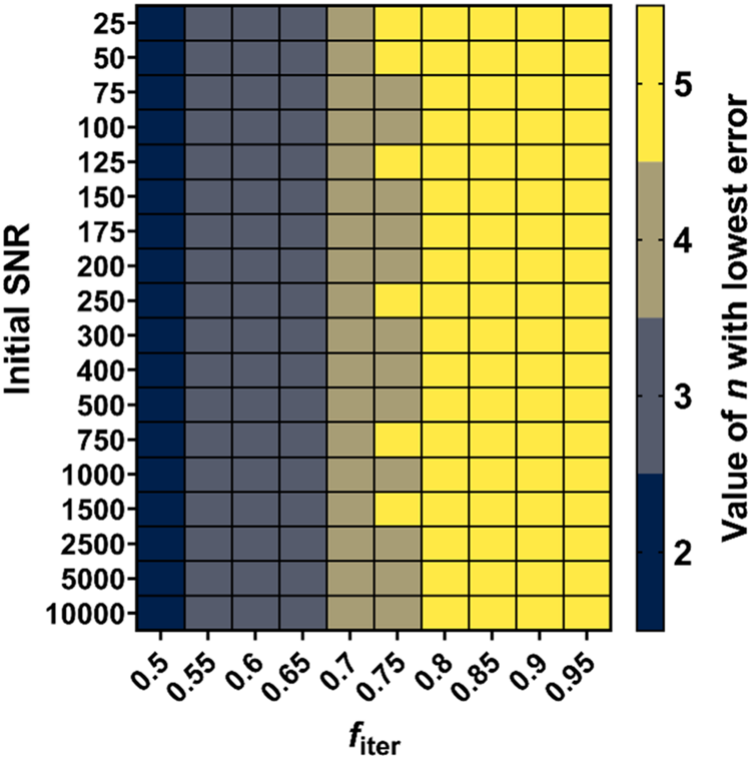
Heatmap showing the optimal value of the number of EXQUISITE iterations to perform for a range of values of *f*_iter_ and initial SNR, determined from Monte Carlo modeling.

Figure 4 shows a heatmap of the optimal value of *n* (which minimizes the quantitation error) for a range of values of *f*_iter_ and initial SNR. Interestingly, the optimal value for *n* decreases with *f*_iter_ but is essentially independent of the initial SNR. This can be rationalized by recognizing that for low values of *f*_iter_, the contribution of noise to integral uncertainty increases significantly with each successive iteration, rendering the inclusion of higher values of *n* detrimental to the quality of extrapolation. Conversely, for high values of *f*_iter_, the noise contribution increases less with each successive iteration but is more significant relative to the signal loss per iteration. Thus, the signal attenuation is more accurately measured by fitting with a greater number of data points. These effects are independent of the initial SNR, even though increasing the initial SNR results in a lower quantitation error for a given value of *f*_iter_.

The important factor that was not considered when determining the optimal values for *n* shown in Figure 4 is the increased experiment time required to perform a greater maximum number of iterations. While the absolute error in quantitation decreases with increasing *n* for high *f*_iter_, the improvement obtained is less than that available from acquiring more transients to raise the SNR. As a result, the optimal *n* is almost always two, if the assumption of Gaussian noise (i.e., random errors) is valid. A possible exception would be in the case of severe convection, where it may be advantageous to acquire only fully convection-compensated iterations (i.e., the second and fourth iterations), particularly if a comparison between the extrapolated integrals from different experiments is desired. Where there are grounds to suspect that systematic errors are present, acquiring higher values of *n* may be justified to identify any nonexponential decay of signal integral as a function of *n*. The same Monte Carlo modeling was also used to estimate the values of *n* required to provide a quantitation error of less than 1% (Figure S11) or 2% (Figure S12) for given values of *f*_iter_ and initial SNR.

## Conclusions

The EXQUISITE method described here allows the acquisition of universally quantitative band-selective pure shift NMR spectra. Relative signal integrals obtained for a mixture of ibuprofen, diethyl ether, and ethyl isovalerate were well within 1% of those from an equivalent pulse-acquire experiment. The technique has been demonstrated with both standard interferogram pure shift acquisition and the much quicker semi-realtime acquisition mode. Band-selective pure shift NMR is especially suited to qNMR, as there is relatively little sensitivity lost due to the ASR element compared to other pure shift methods. We expect that the ability to suppress ^1^H homonuclear coupling in selected spectral regions while retaining quantitative signal integrals will be of great utility in the quantitation of complex mixtures. Although broadband quantitative pure shift spectra are an attractive proposition, the implementation of broadband ASR elements with the EXQUISITE method poses additional challenges. Chief among these is their inherently poorer sensitivity, but unfavorable spin physics can also lead either to impractically severe signal attenuation when repeating the ASR, as in the case of PSYCHE, or to nonexponential signal attenuation. We nevertheless believe that the EXQUISITE method can be usefully extended to design a variety of universally quantitative multiple-pulse 1D and 2D NMR experiments.

## Supporting Information Available

Additional experimental and simulated data; theoretical explanations; pulse sequence and macro codes; and an experiment guide; all files used to perform the simulations. All files used to perform the simulations, along with all experimental data, macros, and pulse sequence codes, are available at DOI 10.48420/25324786 (PDF)
