# Supplementary material for: Universally Quantitative Band-Selective Pure Shift NMR Spectroscopy
Source: Anal Chem. 2024 May 30;96(23):9601–9. doi: 10.1021/acs.analchem.4c01199 (PMC11170551; doi:10.1021/acs.analchem.4c01199)
Supplement: Supplementary file 1 — ac4c01199_si_001.pdf [file ac4c01199_si_001.pdf]

Supporting Information

Universally quantitative band-selective pure shift NMR spectroscopy

Howard M. Foster, Mathias Nilsson, Ralph W. Adams, and Gareth A. Morris*

Department of Chemistry, The University of Manchester, Oxford Rd, Manchester M13 9PL, United Kingdom

* Corresponding author
Email address: gareth.morris@manchester.ac.uk

Contents

S1. Schematic pulse sequences S4

S2. Experimental details S5

S2.1. All NMR experiments S5

S2.2. All EXQUISITE band-selective pure shift NMR experiments S5

S2.3. Doped water NMR experiments S5

S2.3.1. Pulse-acquire NMR experiment S5

S2.3.2. T_1 inversion recovery NMR experiment S5

S2.3.3. EXQUISITE band-selective pure shift NMR experiments S6

S2.4. Doped ethanol NMR experiments S7

S2.4.1. Pulse-acquire NMR experiment S7

S2.4.2. T_1 inversion recovery NMR experiment S7

S2.4.3. EXQUISITE band-selective pure shift NMR experiments S7

S2.5. Ibuprofen, diethyl ether and ethyl isovalerate mixture NMR experiments S8

S2.5.1. Pulse-acquire NMR experiments S8

S2.5.2. T_1 inversion recovery NMR experiments S9

S2.5.3. EXQUISITE band-selective pure shift NMR experiments S9

S3. Guidance for EXQUSITE band-selective pure shift NMR experiments S11

S3.1. Varian/Agilent NMR spectrometers S11

S3.2. Bruker NMR spectrometers S12

S4. EXQUISITE band-selective pure shift NMR quantitation S13

S4.1. Doped water S13

S4.2. Ibuprofen, diethyl ether and ethyl isovalerate mixture S14

S5. Refocusing *J*-evolution at the midpoint of the chunk S17

S6. Influence of *J*-modulation on quantitation S18

S7. Convection compensation in EXQUISITE S20

S8. Coherence transfer pathway (CTP) enforcement S23

S9. Further EXQUISITE SNR analysis S25

S10. Assessment of quantitative performance using Spinach simulations S28

S11. Pulse sequence codes S30

S11.1. Varian/Agilent interferogram acquisition mode pulse sequence code S30

S11.2. Varian/Agilent semi-real-time acquisition mode pulse sequence code S43

S11.3. Bruker interferogram acquisition mode pulse sequence code S58

S12. Varian/Agilent setup and processing macros S65
S12.1. Setup macros S65
S12.1.1. UoM_setup_EXQUISITE S65
S12.1.2. UoM_setup_EXQUISITE_if S66
S12.1.3. UoM_setup_EXQUISITE_srt S69
S12.1.4. UoM_srt_ps_calcs S73
S12.1.5. go_UoM_EXQUISITE_if S74
S12.1.6. go_UoM_EXQUISITE_srt S74
S12.2. Processing macros S75
S12.2.1. UoM_proc_1d_if_array S75
S12.2.1. UoM_proc_1d_srt_array S78

S1. Schematic pulse sequences

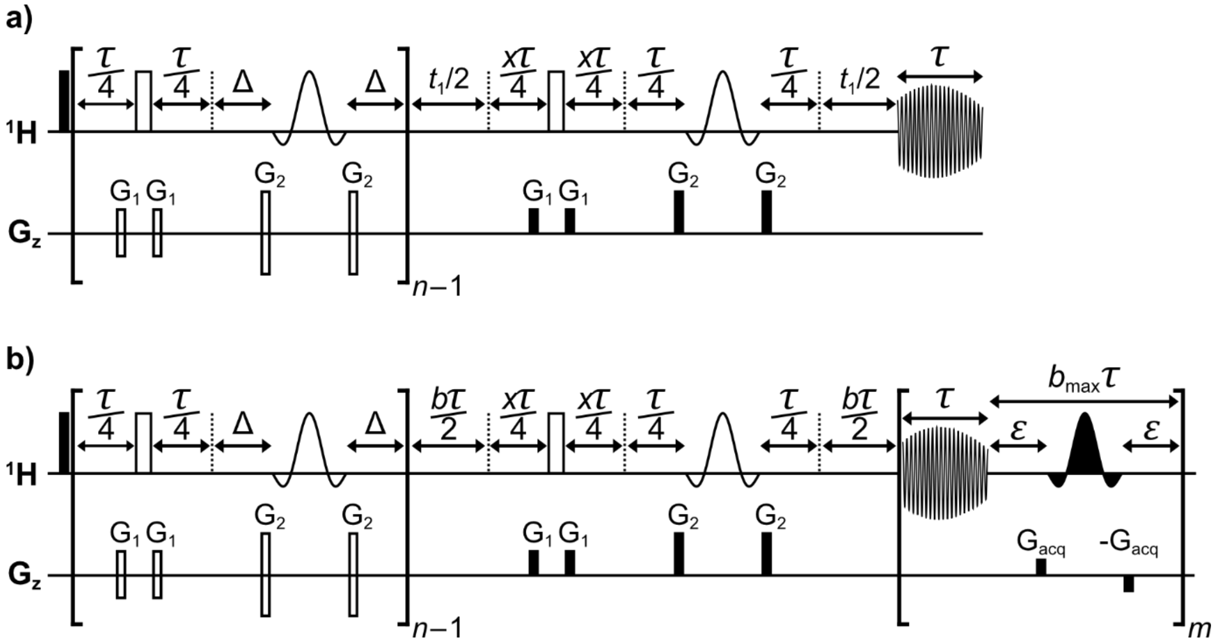

Figure S1. Schematic pulse sequences for quantitative band-selective pure shift NMR with the EXQUISITE method, in (a) interferogram and (b) semi-real-time acquisition mode. Filled and open rectangles on the ^{1}H line represent hard (broadband) 90° and 180° RF pulses, respectively. Filled and open "sinc" (sin[*x*]/*x*) shapes represent selective 180° RF pulses applied to the "passive" and "active" spins, respectively. Rectangles on the G_z line represent rectangular PFGs, with open rectangles representing PFGs for which the polarity is reversed in successive iterations in order to achieve convection compensation. The number of EXQUISITE iterations is *n*, and the chunk duration is τ. The $\tau/4$ delays ensure that *J*-evolution is refocused at the midpoint of the chunk. For odd *n*, *x* takes the value 1; for even *n*, *x* is 2. The Δ delays are adjusted to ensure that the total time between the initial 90° excitation pulse and acquisition (excluding the t_1 evolution periods) is an integer multiple of the duration of a single iteration for both odd and even *n*. The integer *b* (values 0, 1, 2, …) is used to time-shift the chunk acquisition period so that a complete "FID" can be obtained in the semi-real-time acquisition mode. Its maximum value b_{max} is one less than the number of complementary semi-real-time experiments required to construct a complete FID; in this work just two acquisitions were used, restricting *b* to values of 0 and 1. The delays ϵ are adjusted so the total duration of each loop in the semi-real-time acquisition is $(b_{\max}+1)\tau$. The semi-real-time acquisition is looped m times per experiment, so that the final FID/interferogram is composed of $m(b_{\max}+1)$ chunks.

For simplicity, the schematic pulse sequences of **Figure S1** omit the "dropped points" delays (τ_{drop}). These are used to delay the refocusing of chemical shift and scalar coupling evolution during the acquisition period so that the first few points, which are distorted by analogue or digital signal filtration, can be discarded. $\tau_{\mathrm{drop}}=n_{\mathrm{drop}}/sw$, where n_{drop} is the number of complex points to be acquired but then dropped. In both interferogram and semi-real-time acquisition mode a τ_{drop} delay is placed before and after each "active" spin selective 180° RF pulse. The τ_{drop} delay after the final active spin selective 180° RF pulse is incorporated into the start of the acquisition period, so that the points acquired during this time may be discarded. In the semi-real-time acquisition mode the acquisition period is extended by a further delay τ_{drop} so that the final points of each chunk may also be discarded.

S2. Experimental details

S2.1. All NMR experiments

All NMR experiments were performed using either a Varian/Agilent VNMRS500, a Bruker Avance NEO 500, or a Bruker Avance NEO 700 NMR spectrometer, operating at ^{1}H resonance frequencies of 499.83, 500.13, and 700.13 MHz, respectively. The two 500 MHz spectrometers were both equipped with 5 mm room temperature triple resonance (^{1}H, ^{13}C, ^{15}N) probes with triple axis PFG coils of 67 G cm^{-1} maximum nominal *z*-axis strength. The 700 MHz spectrometer was equipped with a 5 mm helium cooled broadband observe probe. The Varian/Agilent spectrometer was controlled with VnmrJ 4.2, and the Bruker spectrometers with TopSpin 4.3.0.

All NMR experiments were acquired with a calibrated 90° initial excitation pulse and an inter-transient recovery delay at least seven times the maximum T_1 value for the signal(s) of interest.

Various software packages were used to process NMR data: VnmrJ 4.2, TopSpin 4.1.3, and the General NMR Analysis Toolbox (GNAT)[1] MATLAB version 1.2.

All experimental and simulated data, macros and simulation and pulse sequence codes are available at DOI 10.48420/25324786.

S2.2. All EXQUISITE band-selective pure shift NMR experiments

All EXQUISITE band-selective pure shift NMR experiments used selective shaped RF pulses; the RF amplitudes and phases, and the durations, of these RF pulses were determined by either Pbox (VnmrJ 4.2) or WaveMaker (TopSpin 4.3.0) for a user-defined nominal bandwidth.

S2.3. Doped water NMR experiments

All doped water NMR experiments were performed using the Varian/Agilent VNMRS500 NMR spectrometer with the probe temperature nominally regulated at 25 °C.

S2.3.1. Pulse-acquire NMR experiment

A proton pulse-acquire experiment on the doped water sample was performed with a spectral width of 5000 Hz, a d1 delay of 3 s, and an acquisition time of 1.6384 s, which corresponded to acquiring 16,384 complex points. The experiment was executed with 32 transients and two steady state transients. The data were processed in VnmrJ: zero-filling the FID to 131,072 real points, phase correction, drift correction, and the application of a Lorentz to Gauss transform (lb = –0.8 Hz, gf = 1).

S2.3.2. T_1 inversion recovery NMR experiment

A proton T_1 inversion recovery experiment on the doped water sample was performed with a spectral width of 5000 Hz, a d1 delay of 2 s, and an acquisition time of 2.0448 s, which corresponded to acquiring 20448 complex points per FID. The delay between the 180° and 90° RF pulses was arrayed

[1] Castañar, L.; Poggetto, G. D.; Colbourne, A. A.; Morris, G. A.; Nilsson, M. The GNAT: A new tool for processing NMR data *Magn. Reson. Chem.* **2018**, *56* (6), 546–558.

from 12.5 ms to 3.2 s in 9 steps. The experiment was executed with four transients, and two steady state transients.

The data were processed in the General NMR Analysis Toolbox (GNAT): zero-filling the FIDs to 131,072 real points, phase correction, 5th order polynomial baseline correction, and the integration of signals of interest. GNAT was used to fit the integrals from the array of spectra to determine T_1 time constants.

S2.3.3. EXQUISITE band-selective pure shift NMR experiments

EXQUISITE band-selective pure shift NMR experiments in interferogram acquisition mode were carried out on the doped water sample to assess the quantitative performance of the method. Four sets of experiments were conducted, with different experimental parameters to cause different attenuation of the water signal. The parameters varied were an additional pair of delays placed either side of the selective 180° RF pulse in each iteration ($\tau_{\mathrm{E}}/2$ for each delay) and the nominal G_2 PFG amplitude. The values of these parameters in each set of experiments are shown in **Table S1**.

Table S1. Experimental parameters varied in the four sets of EXQUISITE band-selective pure shift NMR experiments on the doped water sample.

Experiment	τ_{E} / ms	G_2 PFG amplitude / G cm^{-1}
A	0	20.8
B	20	20.8
C	0	29.4
D	20	29.4

The experiments were performed with spectral widths of 5000 Hz and 25 Hz in the direct and indirect dimensions, respectively, a d1 delay of 0.5 s, and an acquisition time of 1.6384 s, which corresponded to acquiring 16,384 complex points. 10 t_1 increments (or pure shift chunks) were acquired in each experiment. The experiments were executed with 16 transients and two steady state transients. The transmitter offset was placed on resonance with the water signal. The ASR element was a 180° rSNOB pulse with a duration of 9.25 ms, corresponding to a nominal bandwidth of 200 Hz, targeting the water signal. The nominal amplitude of the G_1 PFG was 16.1 cm^{-1}. All PFGs were rectangular in shape. The duration of each PFG and the minimum delay following each was 1 ms. One, two and three EXQUISITE iterations were recorded in each experiment.

The data were assembled into pure shift "FIDs" (interferograms) in VnmrJ using the macro *UoM_proc_1d_if_array* (reproduced in **Section S12.1**). This macro discarded the first 16 complex points from each FID as "dropped" points and extracted the subsequent 200 complex points. These data points were then assembled into a pure shift interferogram for each EXQUISITE iteration. The pure shift FIDs were further processed in VnmrJ: zero-filling to 131,072 real points, phase correction, drift correction, and Gaussian weighting (gf = 0.2). The water signal was integrated over a region 80 Hz wide, corresponding to *ca.* 20 times the full width at half maximum (FWHM) of the signal.

S2.4. Doped ethanol NMR experiments

All doped ethanol NMR experiments were performed using a Varian/Agilent VNMRS500 NMR spectrometer with the probe temperature nominally regulated at 25 °C.

S2.4.1. Pulse-acquire NMR experiment

A proton pulse-acquire experiment on the doped ethanol sample was performed with a spectral width of 5000 Hz, a d1 delay of 3 s, and an acquisition time of 1.6384 s, which corresponded to acquiring 16,384 complex points. The experiment was executed with 32 transients and two steady state transients. The spectrum was processed in VnmrJ: zero-filling the FID to 131,072 real points, phase correction, drift correction, and the application of a Lorentz to Gauss transform (lb = –0.8 Hz, gf = 1).

S2.4.2. T_1 inversion recovery NMR experiment

A proton T_1 inversion recovery experiment on the doped ethanol sample was performed with a spectral width of 5000 Hz, a d1 delay of 8 s, and an acquisition time of 1.6384 s, which corresponded to acquiring 16,384 complex points per FID. The delay between the 180° and 90° RF pulses was arrayed from 25 ms to 12.8 s in 10 steps. The experiment was executed with four transients, and two steady state transients.

The data were processed in the General NMR Analysis Toolbox (GNAT): zero-filling the FIDs to 131,072 real points, phase correction, 5th order polynomial baseline correction, and the integration of signals of interest. GNAT was used to fit the integrals from the array of spectra to determine T_1 time constants.

S2.4.3. EXQUISITE band-selective pure shift NMR experiments

EXQUISITE band-selective pure shift NMR experiments in interferogram acquisition mode were carried out on the doped ethanol sample to assess convection compensation. The experiments were performed with spectral widths of 5000 Hz and 125 Hz in the direct and indirect dimensions, respectively, a d1 delay of 3 s, and an acquisition time of 1.6384 s, which corresponded to acquiring 16,384 complex points. The experiments were executed with 128 transients and four steady state transients. The transmitter offset was placed on resonance with the methylene signal. The ASR element was a 180° rSNOB pulse with a duration of 9.25 ms, corresponding to a nominal bandwidth of 200 Hz, targeting the methylene signal of ethanol. The G_1 and G_2 PFGs were rectangular in shape and had nominal amplitudes of 28.9 and 37.4 G cm^{-1}, respectively. The duration of each PFG was 1 ms and the minimum delay following the application of a PFG was 0.98 ms. One to five EXQUISITE iterations were recorded in each experiment. In one set of experiments, PFG polarities were alternated in successive iterations, to partially compensate for convection. In the other set of experiments, PFG polarities were kept constant. In total 22 experiments were conducted, with the two sets of experiments being interleaved.

Although the interferogram acquisition mode pulse sequence was used, only the first t_1 increment was acquired in each experiment. Each of these increments was Fourier transformed and processed individually, in VnmrJ. The first four complex points from each FID were removed by setting the parameter “lsfid = 4”. Further processing consisted of zero-filling the FIDs to 131,072 real points, phase correction, drift correction, and a Lorentz to Gauss transform (lb = –0.5 Hz, gf = 1). The methylene signal of ethanol was integrated over a region 50 Hz wide.

An analysis of these data may be found in **Section S7**.

S2.5. Ibuprofen, diethyl ether and ethyl isovalerate mixture NMR experiments

S2.5.1. Pulse-acquire NMR experiments

A proton pulse-acquire experiment on the ibuprofen, diethyl ether and ethyl isovalerate (IDE) mixture was conducted on a 500 MHz Bruker Avance NEO NMR spectrometer. The probe temperature was nominally regulated at 25 °C. The experiment had a spectral width of 10,000 Hz, a d1 delay of 32 s, and an acquisition time of 3.2768 s, which corresponded to acquiring 32,768 complex points. The experiment was executed with 32 transients and two steady state transients. The spectrum was processed in TopSpin: zero-filling the FID to 524,288 real points, phase correction, 5th order polynomial baseline correction, and the application of a mild Gaussian weighting (LB = –0.01, GB = 0.012868). Due to the proximity of signals E5 and D1, there was a slight degree of overlap between these signals: the closest peaks in each triplet had a separation of 9.8 Hz, and both peaks had linewidths of *ca.* 1.1 Hz.

Because of the overlap, a proton pulse-acquire NMR spectrum of the mixture was obtained at a higher magnetic field, using a 700 MHz Bruker Avance NEO NMR spectrometer, increasing the separation between the closest peaks of E5 and D1 to 19.8 Hz. This experiment was performed using the same experimental parameters as for the 500 MHz spectrometer, except that the number of transients was doubled to 64, and the d1 delay was set to 34 s. The spectrum was processed in TopSpin: zero-filling the FID to 524,288 real points, phase correction, 5th order polynomial baseline correction, and the application of a mild Gaussian weighting (LB = –0.01, GB = 0.012868). The three selected signals in the mixture (I3, E5, and D1) were integrated over a region 27 times the FWHM of a single peak in each multiplet.

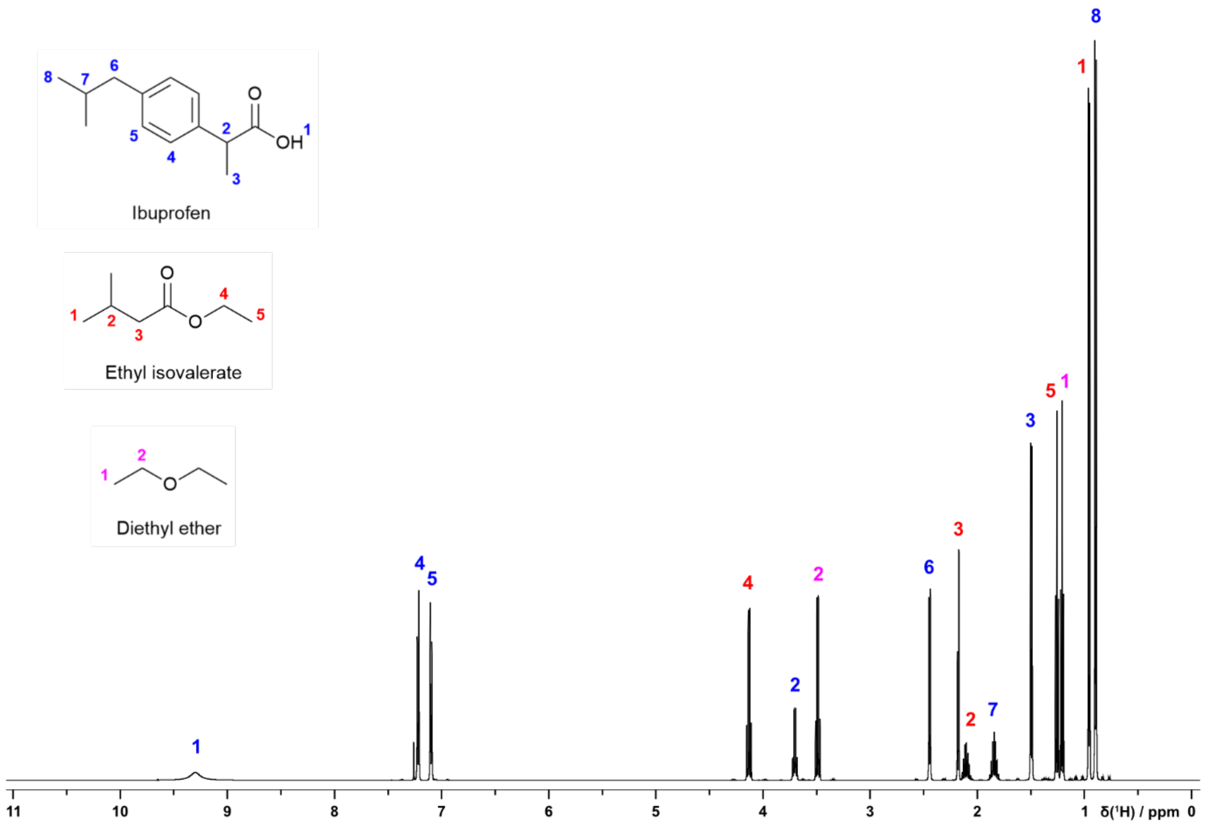

Figure S2. 500 MHz ^{1}H pulse-acquire NMR spectrum of an ibuprofen (154 mM), ethyl isovalerate (125 mM) and diethyl ether (64 mM) mixture in $CDCl_3$, with signal assignments.

S2.5.2. T_1 inversion recovery NMR experiments

Proton T_1 inversion recovery experiments were conducted on the IDE mixture on the Varian/Agilent VNMRS500, and both Bruker Avance Neo NMR spectrometers. The probe temperatures were nominally regulated at 20 °C for the former, and 25 °C for the latter.

The three experiments were performed with a spectral width of 10,000 Hz, a d1 delay of 32 s, and an acquisition time of 3.2768 s, which corresponded to acquiring 32,768 complex points per FID. The delay between the 180° and 90° RF pulses was arrayed from 62.5 ms to 32 s in 10 steps. The experiments were executed with four transients and two steady state transients.

The data were processed in the General NMR Analysis Toolbox (GNAT): zero-filling the FIDs to 131,072 real points, phase correction, 5th order polynomial baseline correction, and the integration of signals of interest. The GNAT was used to fit the integrals from the array of spectra to determine T_1 time constants.

S2.5.3. EXQUISITE band-selective pure shift NMR experiments

EXQUISITE band-selective pure shift NMR experiments in interferogram and semi-real-time acquisition mode were carried out on the IDE mixture. The interferogram mode experiments were conducted on a 500 MHz Bruker Avance NEO NMR spectrometer, with a nominal regulated probe temperature of 25 °C. The semi-real-time mode experiments were conducted on a 500 MHz Varian/Agilent VNMRS500 NMR spectrometer, with a nominal regulated probe temperature of 20 °C.

S2.5.3.1. Both acquisition modes

All experiments used a spectral width of 10,000 Hz in the direct dimension and a d1 delay of 30 s. All experiments were executed with 16 transients and two steady state scans. The transmitter offset was placed at the centre of the frequency range spanned by signals I3, E5 and D1 in all experiments. The ASR element in all experiments was a 180° REBURP pulse with a duration of 24.38 ms, corresponding to a nominal bandwidth of 200 Hz. One to three EXQUISITE iterations were recorded in each experiment, and each experiment was acquired in triplicate.

S2.5.3.2. Interferogram mode

The interferogram mode experiments were performed with a spectral width in the indirect dimension of 80 Hz, corresponding to a chunk duration of 12.5 ms. However, the acquisition time for each t_1 increment was 3.2768 s, which corresponded to acquiring 32,768 complex points. 32 t_1 increments (or pure shift chunks) were acquired in each experiment. The G_1 and G_2 PFGs were 10 % smoothed square (SMSQ10.100) shaped, and had nominal amplitudes of 20.8 and 28.8 G cm^{-1}, respectively. The duration of each PFG and the minimum delay following each was 1 ms.

The data were assembled into pure shift FIDs in TopSpin using the macro *pshift4f*, which is available for download on the website of the NMR Methodology Group at The University of Manchester (https://nmr.chemistry.manchester.ac.uk). This macro discarded the first 8 complex points from each FID as “dropped” points and extracted the subsequent 125 complex points. These data were then assembled into a pure shift interferogram for each EXQUISITE iteration. The pure shift FIDs were further processed in TopSpin: zero-filling to 131,072 real points, phase correction, 5th order polynomial baseline correction, and a mild Gaussian weighting determined by the *setgtc_auto* macro (also available for download from the Manchester NMR Methodology Group website). Each signal was integrated over a range *ca.* six times its FWHM.

S2.5.3.3. Semi-real-time mode

The semi-real-time mode experiments were performed with a spectral width in the indirect dimension of 62.5 Hz, corresponding to a chunk duration of 16 ms. The total acquisition time for each of the two t_1 increments was 1.7136 s, corresponding to acquiring 17,136 complex points. The G_1 and G_2 PFGs were rectangular in shape, and had nominal amplitudes of 18.7 and 25.9 G cm^{-1}, respectively. The duration of each PFG and the minimum delay following the application of PFGs was 1 ms (except for those which flanked the passive spin inversion element). The passive spin inversion element was a 9.75 ms REBURP sine-modulated pulse (500 Hz nominal bandwidth) applied 1229 Hz from the transmitter offset. A 28-step t7m4 phase supercycle was applied to this pulse. PFGs surrounding the passive spin inversion element had a duration of 0.5 ms, and a subsequent recovery delay of 0.4 ms. The nominal amplitudes of these PFGs were cycled through four values during acquisition: 6.3, 8.5, 7.3 and 9.7 G cm^{-1}, to minimise refocusing of unwanted coherence transfer pathways.

The data were assembled into pure shift FIDs in VnmrJ using the macro *UoM_proc_1d_srt_array* (reproduced in **Section S12.2**). This macro divided each FID into data chunks of 168 complex points, discarding the first and last 4 complex points from each. It then interleaved chunks from the first and second t_1 increments to assemble a pure shift FID for each EXQUISITE iteration. The pure shift FIDs were further processed in VnmrJ: zero-filling to 131,072 real points, phase correction, 4th order polynomial baseline correction, and a Gaussian weighting (gf = 1.6384). Each signal was integrated over a range *ca.* 10 times its FWHM.

S3. Guidance for EXQUSITE band-selective pure shift NMR experiments

S3.1. Varian/Agilent NMR spectrometers

The Varian/Agilent pulse sequence code is set up to acquire all EXQUISITE transients within a single experiment, by arraying *numreps*, the number of iterations to be used. Pulse sequence codes for Varian/Agilent spectrometers may be found in **Sections S11.1** and **S11.2**. All Varian/Agilent setup and processing macros may be found in **Section S12.1**. Alternatively, pulse sequence codes and macros may be obtained at DOI 10.48420/25324786.

1. Use the setup macro *UoM_setup_EXQUISITE_if* or *UoM_setup_EXQUISITE_srt* on a proton pulse-acquire experiment. These macros provide sensible starting parameters.
2. Ensure that *pw*, *pw90* and *ref_pw90* are all set to the calibrated 90° RF pulse width.
3. Set *sw* and *sw1*, ensuring that *sw*/*sw1* is an exact integer. If using a correction for *J*-modulation then an *sw1* as low as 50 Hz may be used. If not, it is recommended to use an *sw1* ≥ 100 Hz.
4. Interferogram only: set the number of *droppts*, which is the number of complex points to discard from the beginning of each chunk. This should be an even number ≥ 2.
5. Semi-real-time only: set the number of *droppts1* and *droppts2*, which are the numbers of complex points to discard from the beginning and end of each chunk, respectively. These should both be an even number ≥ 2.
6. Place *tof* at the centre of the active spin region.
7. Semi-real-time only: set *offset* to be an array of values of the centre(s) of the passive spin region(s).
8. Define the bandwidth (in Hz) of the active and passive spin 180° RF pulses (if applicable) with *bw_a* and *bw_p*. A shape other than the default rSNOB may be defined using the parameters *kp_wave_a* and *kp_wave_p* (*e.g.*, 'reburp', 'gaus180r').
9. Interferogram only: set *ni* to the number of t_1 increments (chunks) and ensure that *at* ≥ (1/*sw1* + *droppts*/*sw*).
10. Semi-real-time only: set *np* to be approximately the number of real plus imaginary points in the processed pure shift FID (it will be slightly less due to *droppts1* and *droppts2*).
11. Ensure that *gstab + gt1* < 1/(4*sw1*). Adjust either *gstab* or *gt1*, or both, if necessary.
12. Define the number of EXQUISITE iterations to be performed as an array *numreps* (*e.g.*, to acquire up to three EXQUISITE iterations set *numreps = 1, 2, 3*).
13. Ensure that *nt* is set to at least the minimum number of transients required to complete a full phase cycle
14. Run *go('check')*, which will automatically calculate the duration and power levels of the selective 180° RF pulses. For semi-real-time mode experiments, it will calculate *ni* and recalculate *np* so that only full chunks of data are acquired.
15. Run the experiment.
16. Process the data using the relevant processing macro: *UoM_proc_1d_if_array* or *UoM_proc_1d_srt_array*. These macros generate an array of 1D pure shift NMR spectra.
17. Perform general processing of the spectra, integrate the signals of interest, and carry out an exponential or semi-log fitting to obtain the extrapolated integrals.

S3.2. Bruker NMR spectrometers

The Bruker pulse sequence code is set up to use interferogram mode to acquire only a single number of EXQUISITE iterations, given by the parameter *CNST23*, in any given experiment. For example, if you wish to acquire up to three iterations, you should create three separate experiments, and set *CNST23 = 1* in the first experiment, *CNST23 = 2* in the second experiment, and *CNST23 = 3* in the third experiment. Pulse sequence code for Bruker spectrometers may be found in **Section S11.3**. The processing macro *pshift4f* is available for download on the website of the NMR Methodology Group at The University of Manchester (https://nmr.chemistry.manchester.ac.uk).

1. Set up a 2D dataset experiment and set the *PULPROG* to *UoM_EXQUISITE_if_2d*.
2. Ensure that *P1* is set to the calibrated 90° RF pulse width at the default power level *PLW1*.
3. Set *SWH* in *F1* and *F2*, ensuring that *SWH(F2)*/*SWH(F1)* is an integer. If using a correction for *J*-modulation then an *SWH(F1)* as low as 50 Hz may be used. If not, it is recommended to use an *sw1* ≥ 100 Hz.
4. Set *CNST4*, which is the number of complex points to discard from the beginning of each chunk. This should be an even number ≥ 2.
5. Place *O1* or *O1P* at the centre of the active spin region.
6. Define the bandwidth (in Hz) of the active spin 180° RF pulse with *CNST50*. Define the shape of the pulse in the *USERA1* parameter.
7. Set *TD(F1)* to the number of t_1 increments (chunks) and ensure that *AQ* ≥ (1/*SWH(F1)* + *CNST4*/*SWH(F2)*).
8. Ensure that *D16 + P16 + 50us* < 1/(4*SWH(F1)*). Adjust *D16* if necessary.
9. Ensure that *NS* is set to at least the minimum number of transients required to complete a full phase cycle.
10. Run *gppp* to populate the PFG parameters.
11. Run WaveMaker (*e.g.*, with *wvm -a*) to generate the selective 180° RF pulse.
12. Define the number of EXQUISITE iterations to be performed in that experiment in *CNST23*.
13. Make a copy of that experiment, and add one to the value of *CNST23* in the new experiment. Repeat this step until you have as many experiments as the number of EXQUISITE iterations you would like to acquire.
14. Run the experiments.
15. Generate a 1D pure shift NMR spectrum from each dataset using the macro *pshift4f*.
16. Perform general processing of the spectra, integrate the signals of interest, and carry out an exponential or semi-log fitting to obtain the extrapolated integrals.

WARNING: In recent versions of TopSpin (4.3+) on Windows systems, users may experience an error with the message "The system detected an overrun of a stack-based buffer in this application" when attempting to run the *wvm* command. It seems that this error is related to the length of the phase cycles of the selective 180° RF pulses (ph11 – ph15). Shortening the length of these phase cycles to 64 steps appears to solve the issue, but does preclude the use of experiments with five iterations.

S4. EXQUISITE band-selective pure shift NMR quantitation

S4.1. Doped water

A doped water sample was used as a model system for assessing the quantitative performance of the EXQUISITE band-selective pure shift NMR method. **Figure S3** shows the mean natural logarithm of signal integral *versus* the number of EXQUISITE iterations *n* for the water signal from four sets of experiments acquired with different parameters causing different attenuation of the signal.

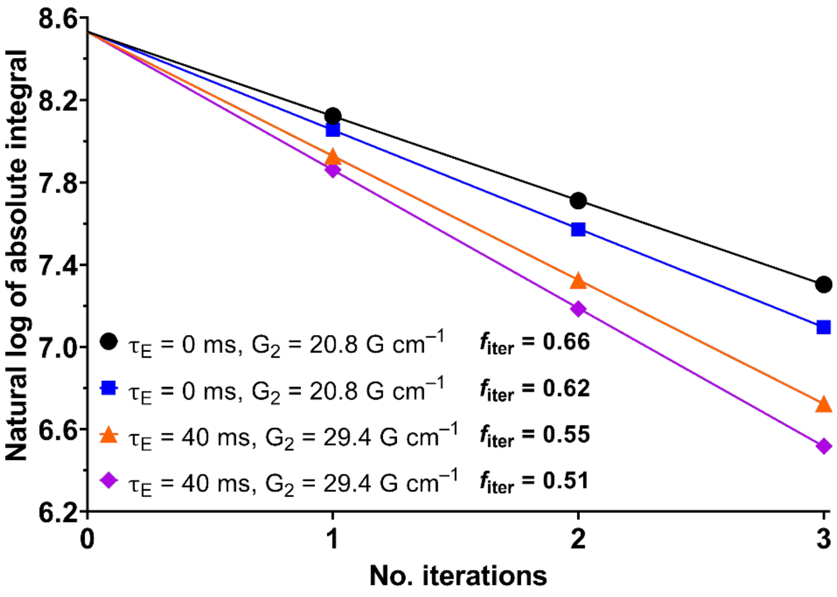

Figure S3. Plots of the natural logarithm of signal integral *versus n* for a doped water signal obtained from four sets of EXQUISITE band-selective pure shift NMR experiments performed with different parameters. The data shown are the means of 13 replicates; error bars are too small to be visible.

For comparison, we performed fitting of the data to an exponential decay for three EXQUISITE iterations. The data obtained from this exponential fitting and linear regression of the semi-log plots is shown in **Table S2**; no statistically significant difference was seen between the two methods.

Table S2. Mean integral deviations obtained by exponential and semi-log fitting of the EXQUISITE band-selective pure shift NMR data for the water signal, using three EXQUISITE iterations.

Experiment	Mean integral deviation from exponential fitting / %	Mean integral deviation from semi-log fitting / %
A	–0.08 (± 0.06)†	0.00 (± 0.06)
B	–0.22 (± 0.06)	–0.17 (± 0.06)
C	0.22 (± 0.04)	0.17 (± 0.04)
D	0.08 (± 0.08)	–0.01 (± 0.08)

†The value quoted in brackets is 1.96 times the standard error of the mean.

S4.2. Ibuprofen, diethyl ether and ethyl isovalerate mixture

Figure S4 shows the attenuation of pure shift signals seen in three iterations of EXQUISITE band-selective pure shift NMR experiments (interferogram and semi-real-time acquisition modes) performed on the ibuprofen, diethyl ether and ethyl isovalerate (IDE) mixture.

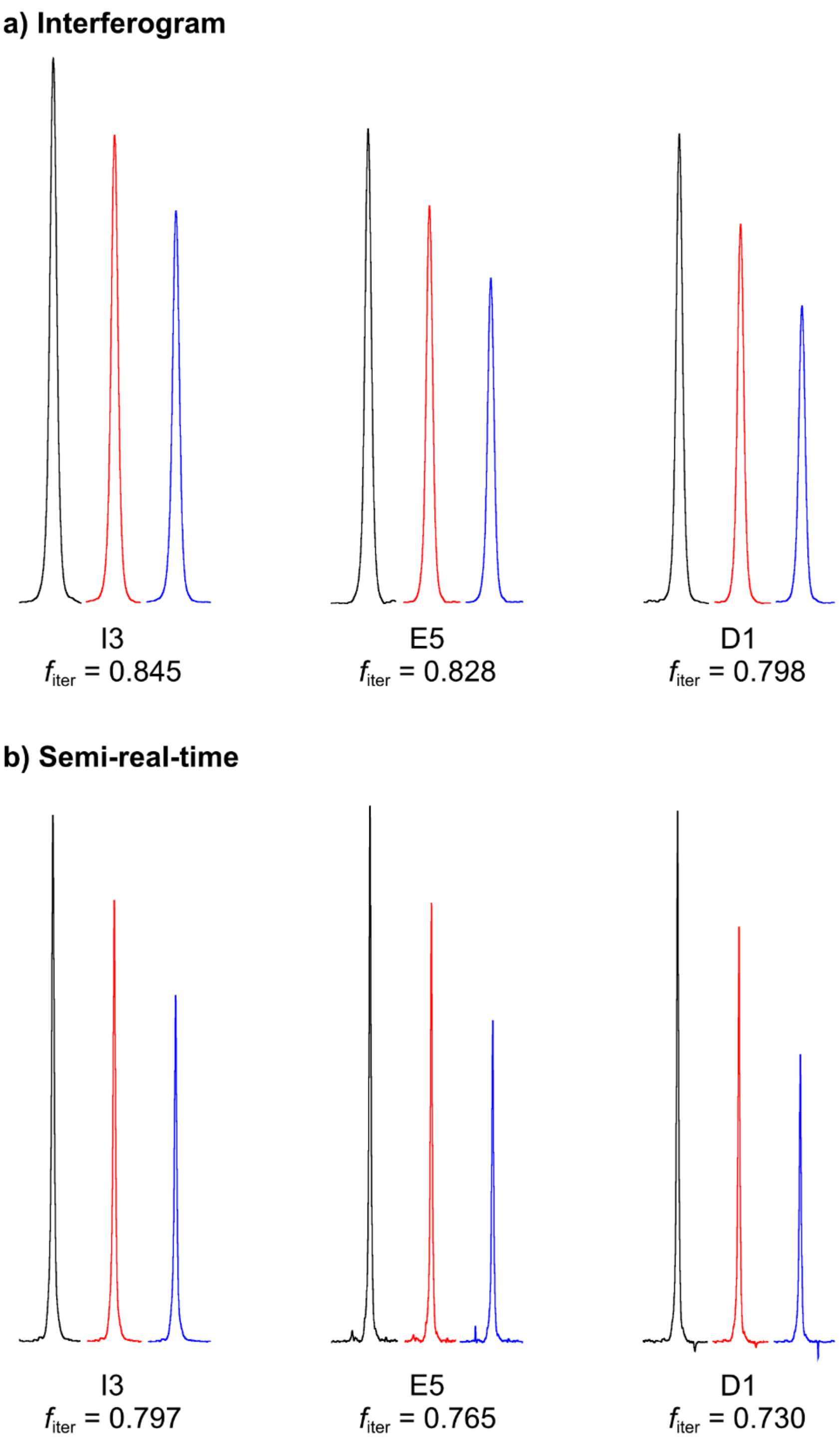

Figure S4. Signals H3 of ibuprofen (I3), H5 of ethyl isovalerate (E5), and H1 of diethyl ether (D1) obtained from the first (black), second (red) and third (blue) iterations of EXQUISITE band-selective pure shift NMR experiments in (a) interferogram and (b) semi-real-time acquisition mode. The mean value of f_{iter} across three replicates is shown below each set of signals. Note that within (a) and (b) all signals were scaled identically, but (a) and (b) were scaled to show similar maximum signal intensities.

Table S3 shows the mixture composition deviations obtained by performing EXQUISITE band-selective pure shift NMR experiments in interferogram (if) and semi-real-time (srt) acquisition mode on the IDE mixture. The EXQUISITE extrapolations were performed using linear regression of the natural logarithm of signal integral as a function of the number of iterations.

The correction for *J*-modulation was determined using eq. S3 (eq. 8 in the main manuscript). The coupling constants for the three signals were measured from the proton pulse-acquire experiment as 7.17, 7.13 and 7.01 Hz for signals B3, E5 and D1, respectively. The chunk durations in the interferogram and semi-real-time mode experiments were 12.5 and 16 ms, respectively.

Table S3. Mixture composition deviations determined using the integrals of signals H3 of ibuprofen (I3), H5 of ethyl isovalerate (E5) and H1 of diethyl ether (D1) in the IDE mixture, from the "base" experiment and extrapolated from two and three iterations of EXQUISITE band-selective pure shift NMR experiments.

**Acq. mode**	**Replicate**	**No. of iterations**	**Mixture composition deviation without correction for *J*-modulation / %**			**Mixture composition deviation with correction for *J*-modulation / %**		
			**I3**	**E5**	**D1**	**I3**	**E5**	**D1**
if	i	Base	0.90	–0.13	–0.77	0.82	–0.08	–0.74
		2	0.10	–0.10	0.00	0.02	–0.05	0.03
		3	0.06	–0.03	–0.03	–0.02	0.03	–0.01
	ii	Base	0.87	–0.10	–0.77	0.79	–0.05	–0.75
		2	0.08	–0.07	–0.02	0.01	–0.01	0.01
		3	0.03	0.01	–0.03	–0.05	0.06	–0.01
	iii	Base	0.92	–0.11	–0.80	0.84	–0.06	–0.78
		2	0.16	–0.08	–0.08	0.09	–0.03	–0.06
		3	0.09	0.00	–0.09	0.01	0.06	–0.07
srt	i	Base	1.58	–0.47	–1.11	1.45	–0.37	–1.07
		2	0.22	–0.15	–0.07	0.09	–0.06	–0.03
		3	0.15	–0.06	–0.09	0.02	0.03	–0.05
	ii	Base	1.57	–0.46	–1.11	1.44	–0.36	–1.07
		2	0.22	–0.14	–0.08	0.09	–0.05	–0.04
		3	0.15	–0.06	–0.08	0.02	0.03	–0.04
	iii	Base	1.55	–0.44	–1.11	1.42	–0.35	–1.07
		2	0.19	–0.08	–0.10	0.06	0.01	–0.06
		3	0.12	–0.05	–0.07	–0.01	0.04	–0.03

Table S4 shows the mixture component concentration deviations obtained by performing EXQUISITE band-selective pure shift NMR experiments in interferogram (if) and semi-real-time (srt) acquisition mode on the IDE mixture. The EXQUISITE extrapolations were performed by linear regression of the natural logarithm of signal integral as a function of the number of iterations. The concentration deviations were obtained by calculating the deviation in composition determined from the EXQUISITE pure shift integrals relative to the composition determined from the pulse-acquire integrals.

Table S4. Mixture component concentration deviations determined from the integrals of signals H3 of ibuprofen (I3), H5 of ethyl isovalerate (E5) and H1 of diethyl ether (D1) in the IDE mixture, from the "base" experiment and extrapolated from two and three iterations and of EXQUISITE band-selective pure shift NMR experiments.

**Acq. mode**	**Replicate**	**No. of iterations**	**Concentration deviation without correction for *J*-modulation / %**			**Concentration deviation with correction for *J*-modulation / %**		
			**I3**	**E5**	**D1**	**I3**	**E5**	**D1**
if	i	Base	2.00	−0.36	−4.09	1.82	−0.21	−3.97
		2	0.22	−0.28	0.01	0.04	−0.13	0.14
		3	0.13	−0.07	−0.16	−0.05	0.08	−0.03
	ii	Base	1.94	−0.28	−4.12	1.76	−0.12	−3.99
		2	0.19	−0.19	−0.09	0.01	−0.04	0.04
		3	0.06	0.02	−0.17	−0.12	0.17	−0.04
	iii	Base	2.04	−0.31	−4.29	1.87	−0.16	−4.17
		2	0.37	−0.22	−0.44	0.19	−0.07	−0.32
		3	0.20	0.01	−0.50	0.03	0.16	−0.37
srt	i	Base	3.51	−1.28	−5.95	3.22	−1.03	−5.74
		2	0.49	−0.41	−0.39	0.21	−0.16	−0.18
		3	0.34	−0.17	−0.49	0.06	0.07	−0.28
	ii	Base	3.49	−1.25	−5.94	3.20	−1.00	−5.73
		2	0.49	−0.38	−0.44	0.21	−0.14	−0.23
		3	0.32	−0.17	−0.45	0.04	0.08	−0.24
	iii	Base	3.46	−1.21	−5.95	3.17	−0.96	−5.74
		2	0.41	−0.23	−0.55	0.13	0.02	−0.34
		3	0.26	−0.14	−0.36	−0.03	0.11	−0.15

S5. Refocusing *J*-evolution at the midpoint of the chunk

Refocusing *J*-evolution at the midpoint of the acquired chunk causes chunking sidebands in pure shift NMR spectra to be in negative absorption mode. Refocusing is achieved by the inclusion of two $\tau/4$ delays, either side of a hard 180° RF pulse, before the active spin refocusing element (a selective 180° RF pulse for band-selective pure shift experiments). Repeating this hard 180° RF pulse and pair of $\tau/4$ delays in a subsequent EXQUISITE iteration results in additional time during which scalar coupling can evolve prior to acquisition, changing the point at which *J* is refocused during the chunk. Thus, in EXQUISITE pure shift sequences it is important to adjust these delays so that *J*-evolution remains refocused at the midpoint of the chunk for every iteration.

Figure S5 shows the net scalar coupling evolution during the delays flanking the hard 180° pulses for EXQUISITE band-selective pure shift experiments with different numbers of iterations. The total duration for which scalar coupling evolves is shown on the right hand side of each pulse sequence, for two different values of *x*, which is a multiplier of the delays around the final hard 180° RF pulse. The sense of *J*-evolution during the delays alternates between iterations, due to the intervening selective 180° pulse that affects only the active spins. For an even number of iterations, if these delays are equal, there is a complete cancellation of scalar coupling evolution, leading to scalar coupling evolution being refocused at the beginning the chunk. Therefore, it is necessary to double the duration of one set of these delays (*i.e.,* to set the value of *x* to be 2) when acquiring an even number of iterations, to refocus scalar coupling evolution at the midpoint of the chunk. Note that if *x* = 2, the durations of other delays in the sequence need to be adjusted so that relaxational attenuation remains the same for every iteration.

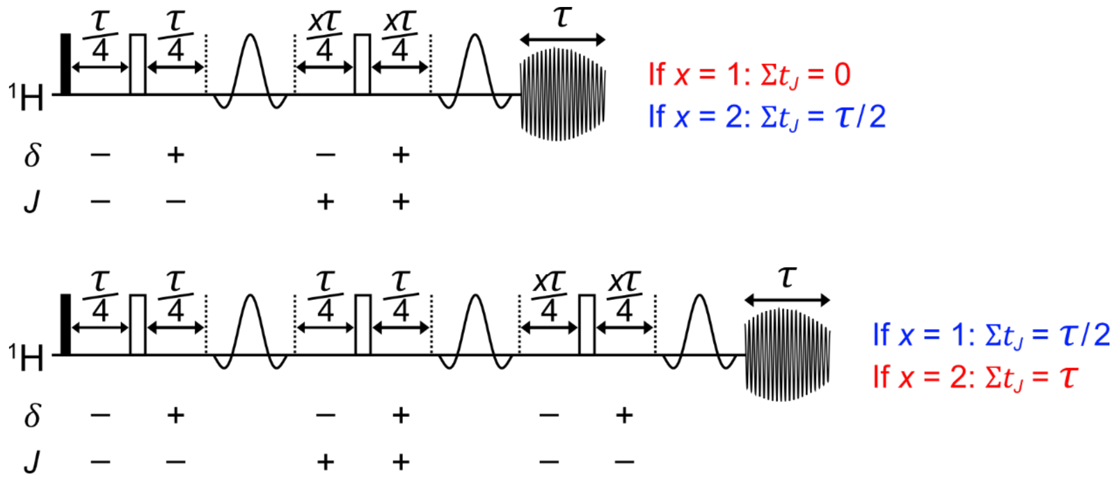

Figure S5. Simplified EXQUISITE band-selective pure shift pulse sequences showing two (top) and three (bottom) iterations. The sense of chemical shift evolution and *J*-evolution during the delays flanking the hard 180° pulses is denoted on the "δ" and "*J*" lines, respectively. Shown to the right of the sequences is the net scalar coupling evolution time Σt_J during the delays flanking the hard 180° pulses when the value of *x* is changed.

S6. Influence of *J*-modulation on quantitation

The periodic refocusing of *J*-evolution in a pure shift interferogram causes sidebands to occur in the spectrum. These appear either side of each peak in the spectrum at integer multiples of the inverse of the chunk duration τ. If the *J*-evolution is refocused at the midpoint of each data chunk, the sidebands appear in negative absorption mode; if *J* is refocused at the start of each chunk they are in dispersion mode, and for other refocusing points the sidebands have mixed phase. These sidebands "steal" signal intensity from the centreband pure shift signal, reducing its integral. The amount of signal lost is dependent on the number of *J*-couplings and the magnitudes of the coupling constants, and is approximately proportional to $1/\tau^2$.

The amplitude of the first point of an FID or interferogram is proportional to the integral of its Fourier transform (the spectrum) over all frequencies. The contribution to this integral, that is the integral of the centreband peak, at the nominal chemical shift, plus all its sidebands, for a particular spin *k* is

$$A_k = A_{k,0} \prod_{s=2}^{S} \cos\left(\frac{\pi J_{ks} \tau}{2}\right) \qquad \textbf{(S1)}$$

where $A_{k,0}$ is the amplitude contribution in the absence of any scalar coupling interactions, *S* is the number of spins in the spin system, J_{ks} are the coupling constants, and τ is the chunk duration. It is generally preferable for chunking sidebands to be in pure absorption mode since this minimises signal overlap. In this case, the integral of the centreband pure shift signal for spin *k* in the spectrum is

$$I_{k,\text{centre}} = \frac{I_{k,0}}{\tau} \int_{-\tau/2}^{\tau/2} \prod_{s=2}^{S} \cos(\pi J_{ks} t)\ dt \qquad \textbf{(S2)}$$

where $I_{k,0}$ is the integral of signal *k* that would be measured in the absence of scalar coupling. The ratio $A_{k,\text{coupl}} = I_{k,\text{centre}}/I_{k,0}$ can be approximated as

$$A_{k,\text{coupl}} = 1 - \frac{\pi^2 \tau^2}{24} \sum_{s=2}^{S} {J_{ks}}^2 \qquad \textbf{(S3)}$$

where terms of order greater than three in τ and *J* are ignored. **Figure S6** shows that this is a very good approximation for the chunk durations typically used in pure shift NMR ($\tau \leq 20$ ms). A Mathematica notebook (*UoM_EXQUISITE_Jmod_correction*) is provided at DOI 10.48420/25324786 which determines $A_{k,\text{coupl}}$ by evaluating eq. S2. **Figure S6** shows a plot of A_{coupl} *versus* chunk duration obtained from numerically simulated "chunked" pure shift FIDs for a relatively wide multiplet in a four-spin system (*J* = 17.2, 10.3 and 7.6 Hz). The numerical simulation was performed in MATLAB by generating the exponentially decaying FID of a single on-resonance spin and introducing "chunking" by multiplication of the FID by $\cos(\pi J t_c)$, where $t_\text{c} = (t \text{ modulo } \tau) - \tau/2$, for each of the coupling constants *J*. The constructed FIDs were Fourier transformed and the centreband pure shift signal was integrated to obtain A_{coupl}. The values obtained for A_{coupl} closely match those predicted by eq. S3 (eq. 8 in the manuscript, dashed line in **Figure S6**) for chunk durations $\leq$20 ms.

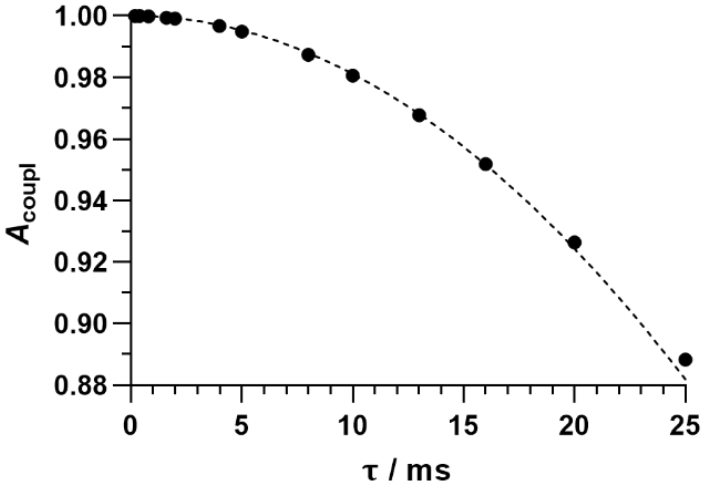

Figure S6. Plot of A_{coupl} *versus* chunk duration obtained from numerically simulated "chunked" pure shift FIDs for a four-spin system (J = 17.2, 10.3 and 7.6 Hz). The dashed line represents the values predicted by eq. 8.

Plots of A_{coupl} *versus* chunk duration for some sample spin systems and scalar coupling constants are shown in **Figure S7**. These data highlight the importance of using short chunk durations ($\tau \leq 10$ ms) when comparing the integrals of signals with significantly different multiplet structure and not using a correction for *J*-modulation.

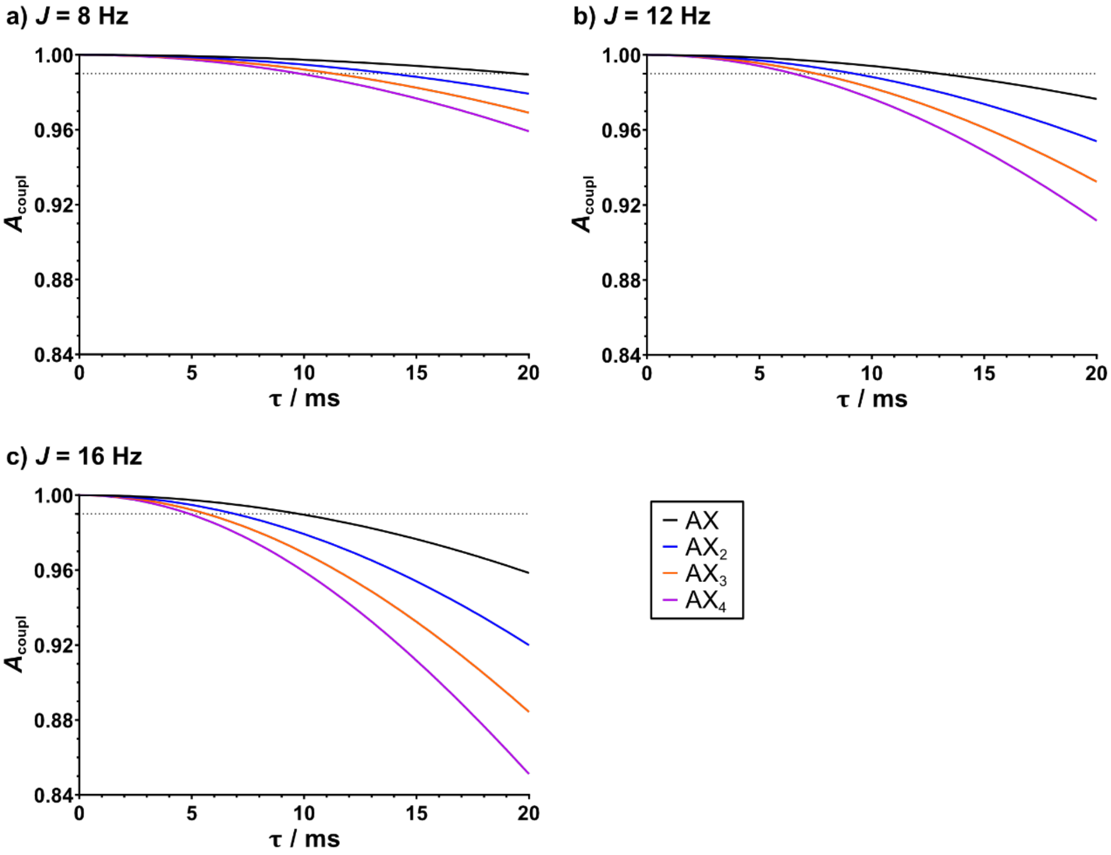

Figure S7. Plots of A_{coupl} *versus* chunk duration for various spin systems (AX, AX_2, AX_3 and AX_4) and scalar coupling constants (8, 12 and 16 Hz) predicted by eq. S2. A dotted line at A_{coupl} = 0.99 is shown in each plot.

S7. Convection compensation in EXQUISITE

As convection is a coherent not a random process, unlike diffusion or spin relaxation, its effect in repeated PFG spin echoes will not be simply multiplicative. The convectional attenuation from *n* iterations of a PFG spin echo will not, even under ideal circumstances, be the convectional attenuation of a single iteration raised to the power *n*. This poses a problem for the EXQUISITE method, which relies on each iteration attenuating signal integrals by the same fraction. However, the coherent nature of convection attenuation provides a way for it to be compensated (at least partially) in EXQUISITE experiments. In the case of EXQUISITE pure shift pulse sequences, this is achieved by alternating the polarity of PFGs in successive iterations.

Figure S8 shows that the signal decays more rapidly if PFG polarities are not alternated in successive iterations, because of sample convection. Alternating PFG polarities in successive iterations means that the convectional attenuation in one iteration is reversed in the next. This leads to complete convection compensation for even-numbered iterations, but residual convectional attenuation (the amount caused by a single iteration) in odd-numbered iterations. The extrapolated integral for the experiment with consistent PFG polarities is *ca.* 27 % higher than that for the experiment with alternating PFG polarities. For these experiments, G_1 and G_2 had nominal amplitudes of 28.9 and 37.4 G cm^{-1}. The sample was ethanol (295 mM) doped with chromium acetylacetonate (*ca.* 3 mM) in $CDCl_3$. The data points plotted are the mean of eleven replicates of each experiment.

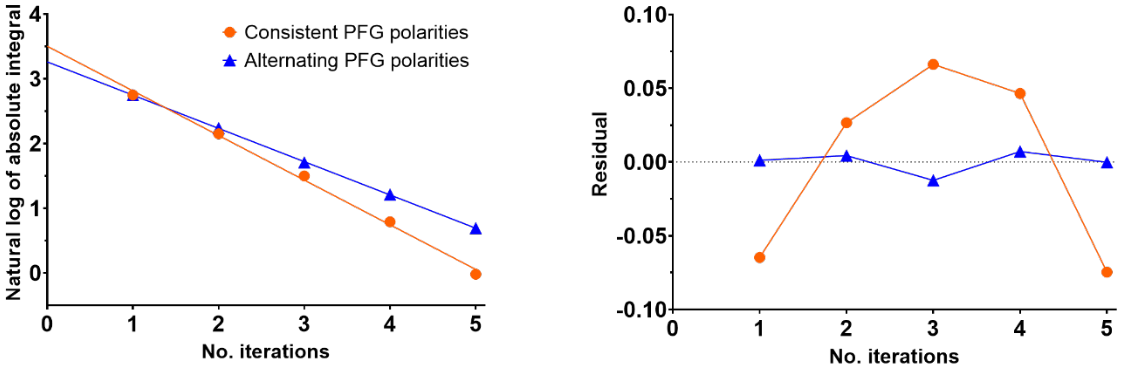

Figure S8. (Left) plot of the natural logarithm of mean absolute integral for the methylene signal of ethanol in $CDCl_3$ *versus* the number of iterations. The integrals were obtained from the FTs of the first increments of two sets of interferogram EXQUISITE band-selective pure shift NMR experiments, one with PFG polarities alternating in successive iterations (blue triangles) and one with consistent PFG polarities in each iteration (orange circles). (Right) plot of the linear regression residuals for these data.

The reduction in signal integral caused by convection driven by a uniform horizontal temperature gradient in an infinite cylinder, in a simple PFG spin echo using rectangular PFGs is given by[2]

$$f_{\mathrm{c}} = 2\int_0^1 rJ_0\left(\frac{3\sqrt{3}}{2}v_{z,\mathrm{max}}kr[1-r^2]\right)drdk \qquad \textbf{(S4)}$$

[2] Swan, I.; Reid, M.; Howe, P. W. A.; Connnel, M. A.; Nilsson, M.; Moore, M. A.; Morris, G. A. Sample convection in liquid-state NMR: why it is always with us, and what we can do about it *J. Magn. Reson.* **2015**, *252*, 120–129.

where J_0 is a Bessel function of the first kind, $v_{z,\max}$ is the maximum convectional flow velocity parallel to the *z*-axis, *r* is the tube radius, $k = \gamma\delta g\Delta$, γ is the gyromagnetic ratio, δ is the gradient duration, g is the gradient amplitude, and Δ is the time for which convection causes signal attenuation.

If the distribution of convective flow velocities is instead approximated as uniform across the range $-v_{z,\max}$ to $+v_{z,\max}$ and zero outside this range, f_c is given by

$$f_c = \frac{\sin(v_{z,\max}\gamma\delta g\Delta)}{v_{z,\max}\gamma\delta g\Delta} \quad \textbf{(S5)}$$

where Δ is the time between the midpoints of a pair of PFGs. The uniform velocity distribution approximation underestimates the contribution of small velocities, but is commonly applied in the measurement of NMR convection velocities, and was applied here in *Spinach*[3] simulations for modelling convection.

Integration of the Bloch-Torrey equations allows the convectional attenuation for *n* iterations of the sequence to be determined (see the Mathematica notebook *UoM_EXQUISITE_convection_analysis* at DOI 10.48420/25324786). Where the PFG polarities are consistent in successive iterations, the overall convectional attenuation is, applying the approximation in eq. S5,

$$f_{c,n} = \frac{\sin(nk)}{nk} \quad \textbf{(S6)}$$

where $k = v_{z,\max}\gamma(\delta_1 g_1 \Delta_1 - \delta_2 g_2 \Delta_2)$. However, when PFG polarities are alternated in successive iterations, the convectional attenuation is zero for even-numbered iterations and is limited to that which occurs in a single iteration for odd-numbered iterations. Thus, the ratio between integrals from experiments where PFG polarities are consistent (I_{con}) and those where polarities are alternated (I_{alt}) is, for odd-numbered iterations,

$$\frac{I_{con}}{I_{alt}} = \frac{k\sin(nk)}{nk\sin(k)} \quad \textbf{(S7)}$$

Figure S9 shows the result of fitting integral ratios from experiments and from *Spinach* simulations to eq. S7. The fitting of the experimental data gave a value of $v_{z,\max}$ of 0.36 mm s^{-1}, and the fitting of simulated data returned a value of $v_{z,\max}$ of 1.008 mm s^{-1} (expected $v_{z,\max}$ = 1 mm s^{-1}). These fits confirm that the loss in signal integral when using fixed rather than alternating PFG polarities is explained by convectional attenuation.

[3] Hogben, H. J.; Krzystyniak, M.; Charnock, G. T. P.; Hore, P. J.; Kuprov, I. Spinach--a software library for simulation of spin dynamics in large spin systems *J. Magn. Reson.* **2011**, *208* (2), 179–194.

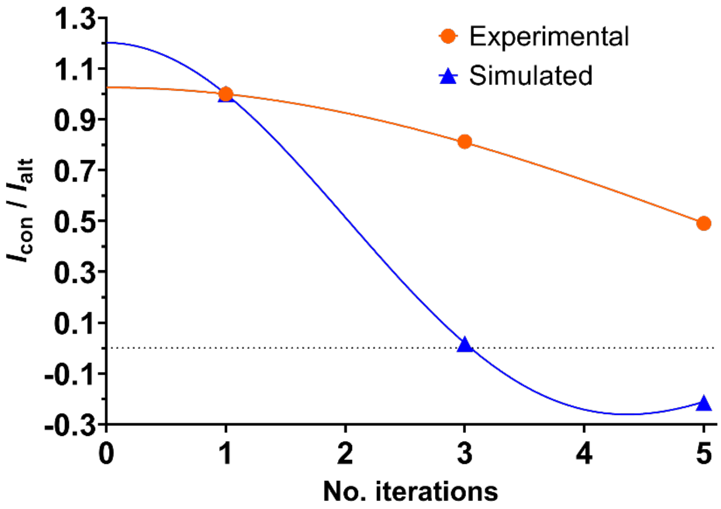

Figure S9. Plot of experimental (orange circles) and simulated (blue triangles) data fitted to eq. S7.

Spinach simulations were performed in the *imaging* context with 1000 points in a spatial grid spanning 1.5 cm along the *z*-axis. Eleven spatial points in an orthogonal dimension were used to provide a linearly spaced distribution of flow velocities from $-v_{z,\max}$ to $+v_{z,\max}$ (1 mm s^{-1}). There was no interaction between these eleven separate slices: diffusion was modelled only along the *z*-direction (with D = 5 x 10^{-10} m^2 s^{-1}). The spin system simulated was a single spin with T_1 and T_2 time constants of 2 and 0.6 s, respectively. The G_1 and G_2 PFGs were simulated using the *evolution* function, with amplitudes of 0.354 and 0.403 T m^{-1}, respectively. RF pulses were simulated using the *step* function. The on-resonance selective 180° RF pulses were 9.25 ms rSNOB shaped pulses simulated using 1000 time points. Explicit phase cycling of the selective RF pulses was implemented, with a total of 128 transients. Only the FID of the first t_1 increment (consisting of 32772 complex points) was simulated for each iteration. The first four complex points of each FID were discarded as dropped points. The FIDs were then exported to TopSpin, Fourier transformed, processed (SI = 131072, PHC0 = 90, LB = –0.53, GB = 0.1) and integrated over a region 20 times the FWHM of the peak to give the blue data points of **Figure S9**.

S8. Coherence transfer pathway (CTP) enforcement

CTP enforcement in the EXQUISTE band-selective pure shift NMR sequences is impaired by the requirement of consistent PFG amplitudes in each successive EXQUISITE iteration. This can lead to certain undesirable CTPs generating observable magnetisation at the beginning of acquisition. We refer to these CTPs as “stimulated echo CTPs”, as they arise from an imperfect 180° RF pulse converting single-quantum coherences to longitudinal magnetisation, which is converted back to single-quantum coherence by a subsequent imperfect 180° RF pulse. **Figure S10** shows an example of the desired CTP and a possible stimulated echo CTP for a two-iteration EXQUISITE band-selective pure shift NMR experiment. In **Figure S10**, both selective 180° RF pulses are assumed to be imperfect. Although the first of the two G_2 PFGs highlighted by the dashed blue boxes will dephase magnetisation following the stimulated echo CTP, the second highlighted G_2 PFG will completely refocus this magnetisation. As PFGs do not affect longitudinal magnetisation, the intervening G_2 PFGs have no effect. Thus, we must rely on independent phase cycling of the selective 180° RF pulses in the sequence to suppress this stimulated echo CTP. In the EXQUISITE band-selective pure shift sequences, these pulses are phase cycled in an alternating two-step (*x*, *y*), four-step pattern (*x*, *y*, –*x*, –*y*) pattern, which is shown in full in **Table S5**.

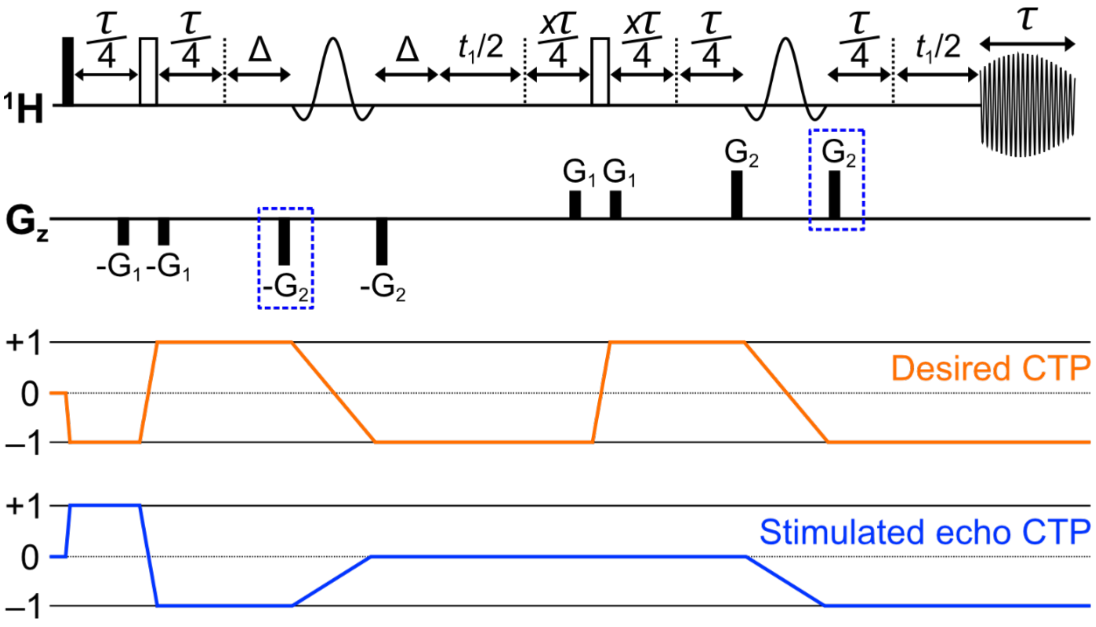

Figure S10. Schematic pulse sequence for a two-iteration EXQUISITE band-selective pure shift NMR experiment, with the desired (orange) and interfering stimulated echo (blue) CTPs shown underneath. The stimulated echo CTP assumes that both selective 180° RF pulses are imperfect.

It is important to note that we have shown only one possible stimulated echo CTP, which leads to observable magnetisation and is not suppressed by PFGs in the sequence. Other possible CTPs may lead to unobservable magnetisation at the beginning of acquisition, or may be suppressed by PFGs.

Table S5. Phase cycles for five selective 180° RF pulses in the EXQUISITE band-selective pure shift NMR sequences.

Pulse no.	Phase cycle	Minimum number of steps
1	x, y	2
2	x_2, y_2, $-x_2$, $-y_2$	8
3	x_8, y_8	16
4	x_{16}, y_{16}, $-x_{16}$, $-y_{16}$	64
5	x_{64}, y_{64}	128

Here, we have assumed that the selective 180° RF pulses are imperfect, but an equivalent argument can be made in the case of the two hard 180° RF pulses being imperfect, and thus giving rise to a stimulated echo CTP. However, as selective 180° RF pulses are more susceptible to miscalibration and imperfections, independent phase cycling of these pulses should be prioritised. Independent phase cycling of the hard 180° RF pulses in addition would make the minimum number of phase cycling steps impractically large.

S9. Further EXQUISITE SNR analysis

The importance of signal-to-noise ratio (SNR) for quantitative accuracy in NMR is widely appreciated. One might assume that for the EXQUISITE method, the more iterations *n* utilised in the extrapolation, the more accurate the extrapolated integral would be. However, as SNR decreases with increasing *n*, there comes a point at which the uncertainty contributed by the noise means that the inclusion of further iterations decreases the accuracy of the extrapolated integral.

We performed Monte Carlo modelling in MATLAB to assess how the initial SNR (*i.e.*, the SNR in a pulse-acquire experiment), fraction of signal retained per iteration f_{iter}, and number of iterations *n*, affected quantitative accuracy. In these simulations, Gaussian noise was added to a Gaussian shaped singlet signal, which was integrated over a region five times its FWHM. The amplitude of the signal was scaled to simulate different initial SNR values and was attenuated perfectly by each EXQUISITE iteration for a range of values of f_{iter}. The quantitation error was calculated as $2\sqrt{2}\sigma_{\mathrm{rel}}$, where σ_{rel} is the relative standard deviation of the extrapolated integral values of the Gaussian singlet signal. The $\sqrt{2}$ factor accounts for the error in comparing two Gaussian singlet signals, with the same initial SNR and value of f_{iter}. **Figure S11a** shows the results of Monte Carlo modelling to find the values of *n* for which the quantitation error is less than 1 %. **Figure S11b** shows the corresponding SNR values for which the quantitation error using a pulse-acquire experiment is within 1 %. **Figure S12** is equivalent to **Figure S11**, but with an error threshold of 2 % rather than 1 %.

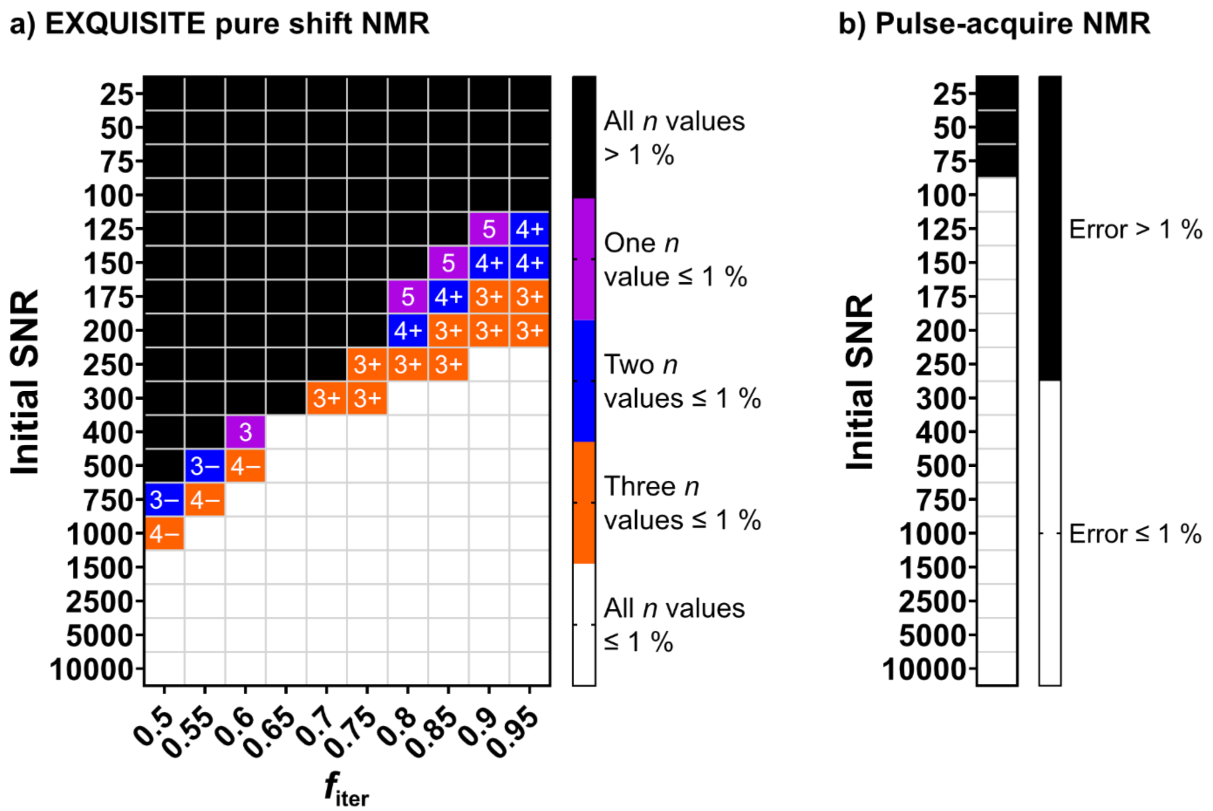

Figure S11. (a) Heatmap of Monte Carlo simulations showing the error in quantitation when using the EXQUISITE method with various values for initial SNR, fraction of signal retained with each EXQUISITE iteration f_{iter}, and number of EXQUISITE iterations *n* (2–5) used in the extrapolation. Black and white squares represent an error exceeding and within 1 %, respectively, for all values of *n*. Purple, blue and orange squares correspond to an error within 1 % for one, two and three values of *n*, respectively, with the specific values of *n* noted in the text within the square: "3+" indicates an error within 1 % for n_m = 3, 4 and 5; "3–" indicates an error within 1 % for *n* = 2 and 3. (b) Heatmap of Monte Carlo simulations assessing the error in quantitation for a pulse-acquire experiment.

Figure S11a clearly shows the expected inverse relationship between quantitation error and SNR (both the initial SNR, and the SNR in later iterations, which is determined by f_{iter}. There are two regions of the plot where the interplay of SNR and *n* value is more subtle. The first of these is the cluster of coloured (purple, blue and orange) squares with higher values of f_{iter} and lower initial SNR values, at the top right hand corner of **Figure S11a**, where increasing *n* results in lower quantitation error. This can be rationalised by noting that for high values of f_{iter}, the noise contribution to the signal integral is greater relative to the signal loss per iteration than it is for low f_{iter} values. The result of this is more uncertainty in the integrals derived from individual *n* values, which can be offset somewhat by utilising more data points (greater *n*) in the extrapolation. The opposite is true for the second cluster of coloured squares (at the bottom left hand corner of **Figure S11a**), at lower f_{iter} values and higher initial values of SNR, where a lower value of *n* gives a lower quantitation error. This can be rationalised by noting that for low values of f_{iter}, the contribution of noise to integral uncertainty increases significantly with each successive iteration, rendering the later iterations, with higher values of *n*, detrimental to the extrapolation.

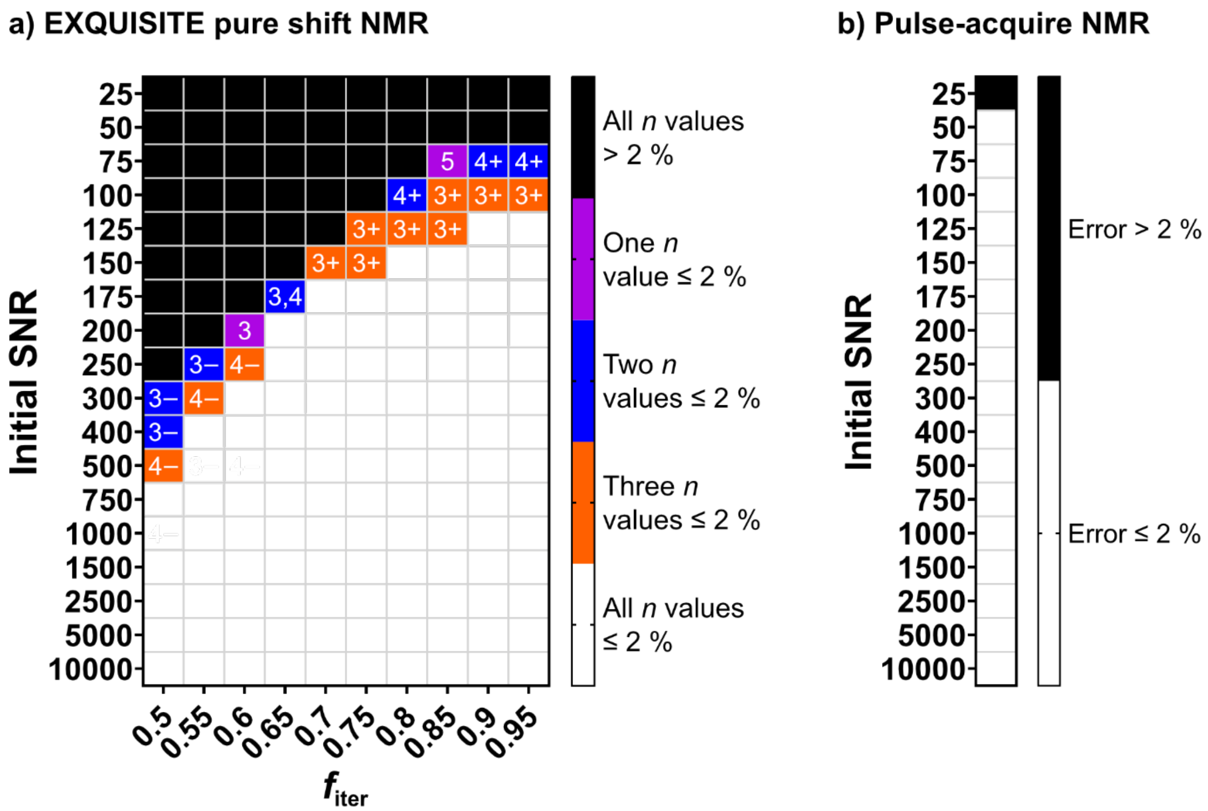

Figure S12. Heatmaps of Monte Carlo simulations showing the error in quantitation when using the EXQUISITE method (a) and a pulse-acquire experiment (b), as in **Figure S11** but with an error threshold of 2 %.

S10. Assessment of quantitative performance using Spinach simulations

Quantitation in interferogram acquisition mode EXQUISITE band-selective pure shift NMR was investigated using *Spinach* simulations of AX spin systems with four different combinations of relaxation and diffusion properties (**Figure S13**). The same AX spin system (δ_A = 4.9987 ppm and δ_X = 1.2500 ppm, J_{AX} = 8 Hz) was used throughout. The simulations were performed in the *imaging* context with 1000 points in a spatial grid spanning 1.5 cm, so that the effects of coherence selection with PFGs and diffusional attenuation could be simulated. The G_1 and G_2 PFGs were simulated using the *evolution* function, with amplitudes of 0.354 and 0.403 T m^{-1}, respectively. RF pulses were simulated using the *step* function. The on-resonance selective 180° RF pulses were 37 ms rSNOB shaped pulses simulated using 1000 time points. Phase cycling of the selective RF pulses was implemented, with a 16-step cycle. The first four complex points of each chunk were discarded as dropped points, and the following 8 ms worth of points from each chunk were used to construct pure shift interferograms consisting of 80 chunks. The simulated interferograms were exported to TopSpin, Fourier transformed, processed (SI = 131072, PHC0 = 90, LB = –0.53, GB = 0.01, AQ = 0.64) and integrated over a region 14 times the FWHM of the peak. The extrapolated integrals from the four simulations had a relative standard deviation (RSD) of 0.135 %.

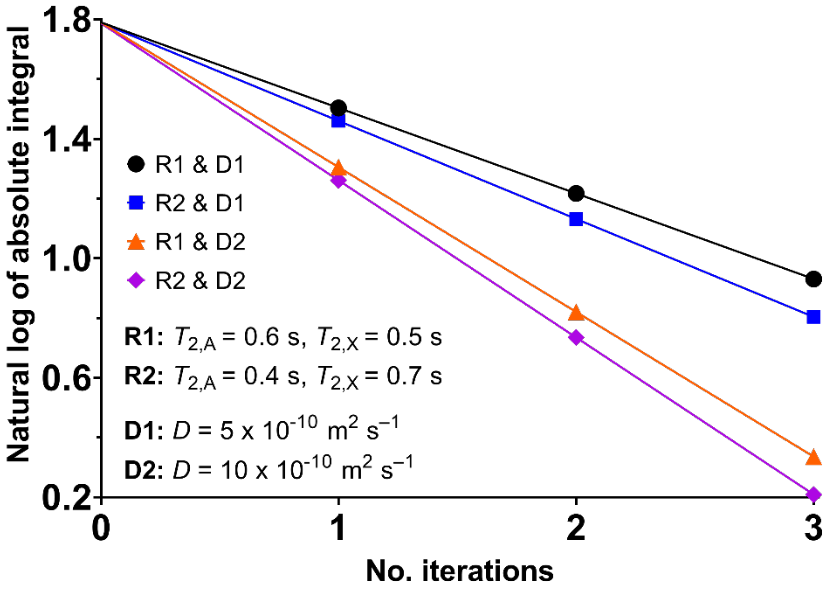

Figure S13. Plot of the natural logarithm of absolute integral vs number of iterations for the “A” signal in four Spinach simulations of the EXQUISITE band-selective pure shift NMR sequence in interferogram acquisition mode on an AX spin system with different combinations of relaxation and diffusion parameters, labelled R1 and R2, and D1 and D2, respectively.

Table S6 shows the relative standard deviations of the four extrapolated integrals when using different simulation parameters and integral region widths. Using more points in the spatial grid and to represent the shaped pulse waveform resulted in small reductions in the extrapolated integral RSD. Expanding the integral region to cover the entire spectrum nullifies the effect of lineshape variation, arising from different relaxation properties, on integration and thus results in much smaller RSDs. The quantitation by extrapolation is essentially perfect if sufficient data points are used in the simulation, and if the full signal integrals are measured.

Table S6. Relative standard deviation between four extrapolated integrals from sets of *Spinach* simulations with different simulation parameters and integral region widths

Shape points	Spatial grid points	Integral region width	Extrapolated integral RSD / %
1000	1000	14 × FWHM	0.135
2000	1000	14 × FWHM	0.112
1000	2000	14 × FWHM	0.111
1000	1000	Full spectrum	0.016
2000	1000	Full spectrum	0.016
1000	2000	Full spectrum	0.005

S11. Pulse sequence codes

S11.1. Varian/Agilent interferogram acquisition mode pulse sequence code

```
/* UoM_EXQUISITE_if --------------------------------------------------------

 EXQUISITE band-selective/Zangger-Sterk pure shift NMR experiment with
 interferogram acquisition.

 Howard M. Foster
 The University of Manchester
 Manchester NMR Methodology Group
 v1.0 29/07/2023
---------------------------------------------------------------------------*/

#include <standard.h>

// Quadrature phase cycling
static int ph1[128] = {0,0, 0,0, 0,0,0,0, 0,0,0,0,0,0,0,0, 0,0,0,0,0,0,0,0,0,0,0,0,0,0,0,0,
0,0,0,0,0,0,0,0,0,0,0,0,0,0,0,0,0,0,0,0,0,0,0,0,0,0,0,0,0,0,0,0,
0,0,0,0,0,0,0,0,0,0,0,0,0,0,0,0,0,0,0,0,0,0,0,0,0,0,0,0,0,0,0,0,0,0,0,0,0,0,0,0,0,0,0,0,0,0,0,
0,0,0,0,0,0,0,0,0,0,0,0,0,0,0,0,0}; // initial 90
static intph2[128] = {0,0, 0,0, 0,0,0,0, 0,0,0,0,0,0,0,0, 0,0,0,0,0,0,0,0,0,0,0,0,0,0,0,0,
0,0,0,0,0,0,0,0,0,0,0,0,0,0,0,0,0,0,0,0,0,0,0,0,0,0,0,0,0,0,0,0,
0,0,0,0,0,0,0,0,0,0,0,0,0,0,0,0,0,0,0,0,0,0,0,0,0,0,0,0,0,0,0,0,0,0,0,0,0,0,0,0,0,0,0,0,0,0,0,
0,0,0,0,0,0,0,0,0,0,0,0,0,0,0,0,0}; // hard 180

static int ph21[128] = {0,1, 0,1, 0,1,0,1, 0,1,0,1,0,1,0,1, 0,1,0,1,0,1,0,1,0,1,0,1,0,1,0,1,
0,1,0,1,0,1,0,1,0,1,0,1,0,1,0,1,0,1,0,1,0,1,0,1,0,1,0,1,0,1,0,1,
0,1,0,1,0,1,0,1,0,1,0,1,0,1,0,1,0,1,0,1,0,1,0,1,0,1,0,1,0,1,0,1,0,1,0,1,0,1,0,1,0,1,0,1,0,1,0,
1,0,1,0,1,0,1,0,1,0,1,0,1,0,1,0,1}; // 1st iteration sel. 180
static int ph22[128] = {0,0, 1,1, 2,2,3,3, 0,0,1,1,2,2,3,3, 0,0,1,1,2,2,3,3,0,0,1,1,2,2,3,3,
0,0,1,1,2,2,3,3,0,0,1,1,2,2,3,3,0,0,1,1,2,2,3,3,0,0,1,1,2,2,3,3,
0,0,1,1,2,2,3,3,0,0,1,1,2,2,3,3,0,0,1,1,2,2,3,3,0,0,1,1,2,2,3,3,0,0,1,1,2,2,3,3,0,0,1,1,2,2,3,
3,0,0,1,1,2,2,3,3,0,0,1,1,2,2,3,3}; // 2nd iteration sel. 180
static int ph23[128] = {0,0, 0,0, 0,0,0,0, 1,1,1,1,1,1,1,1, 0,0,0,0,0,0,0,0,1,1,1,1,1,1,1,1,
0,0,0,0,0,0,0,0,1,1,1,1,1,1,1,1,0,0,0,0,0,0,0,0,1,1,1,1,1,1,1,1,
0,0,0,0,0,0,0,0,1,1,1,1,1,1,1,1,0,0,0,0,0,0,0,0,1,1,1,1,1,1,1,1,0,0,0,0,0,0,0,0,1,1,1,1,1,1,1,
1,0,0,0,0,0,0,0,0,1,1,1,1,1,1,1,1}; // 3rd iteration sel. 180
static int ph24[128] = {0,0, 0,0, 0,0,0,0, 0,0,0,0,0,0,0,0, 1,1,1,1,1,1,1,1,1,1,1,1,1,1,1,1,
2,2,2,2,2,2,2,2,2,2,2,2,2,2,2,2,3,3,3,3,3,3,3,3,3,3,3,3,3,3,3,3,
0,0,0,0,0,0,0,0,0,0,0,0,0,0,0,0,1,1,1,1,1,1,1,1,1,1,1,1,1,1,1,1,2,2,2,2,2,2,2,2,2,2,2,2,2,2,2,
2,3,3,3,3,3,3,3,3,3,3,3,3,3,3,3,3}; // 4th iteration sel. 180
static int ph25[128] = {0,0, 0,0, 0,0,0,0, 0,0,0,0,0,0,0,0, 0,0,0,0,0,0,0,0,0,0,0,0,0,0,0,0,
0,0,0,0,0,0,0,0,0,0,0,0,0,0,0,0,0,0,0,0,0,0,0,0,0,0,0,0,0,0,0,0,
1,1,1,1,1,1,1,1,1,1,1,1,1,1,1,1,1,1,1,1,1,1,1,1,1,1,1,1,1,1,1,1,1,1,1,1,1,1,1,1,1,1,1,1,1,1,1,
1,1,1,1,1,1,1,1,1,1,1,1,1,1,1,1,1}; // 5th iteration sel. 180

static int ph31[128] = {0,2, 0,2, 0,2,0,2, 0,2,0,2,0,2,0,2, 0,2,0,2,0,2,0,2,0,2,0,2,0,2,0,2,
0,2,0,2,0,2,0,2,0,2,0,2,0,2,0,2,0,2,0,2,0,2,0,2,0,2,0,2,0,2,0,2,
0,2,0,2,0,2,0,2,0,2,0,2,0,2,0,2,0,2,0,2,0,2,0,2,0,2,0,2,0,2,0,2,0,2,0,2,0,2,0,2,0,2,0,2,0,2,0,
2,0,2,0,2,0,2,0,2,0,2,0,2,0,2,0,2}; // 1st iteration rec. phase
static int ph32[128] = {0,2, 2,0, 0,2,2,0, 0,2,2,0,0,2,2,0, 0,2,2,0,0,2,2,0,0,2,2,0,0,2,2,0,
0,2,2,0,0,2,2,0,0,2,2,0,0,2,2,0,0,2,2,0,0,2,2,0,0,2,2,0,0,2,2,0,
0,2,2,0,0,2,2,0,0,2,2,0,0,2,2,0,0,2,2,0,0,2,2,0,0,2,2,0,0,2,2,0,0,2,2,0,0,2,2,0,0,2,2,0,0,2,2,
0,0,2,2,0,0,2,2,0,0,2,2,0,0,2,2,0}; // 2nd iteration rec. phase
static int ph33[128] = {0,2, 2,0, 0,2,2,0, 2,0,0,2,2,0,0,2, 0,2,2,0,0,2,2,0,2,0,0,2,2,0,0,2,
0,2,2,0,0,2,2,0,2,0,0,2,2,0,0,2,0,2,2,0,0,2,2,0,2,0,0,2,2,0,0,2,
0,2,2,0,0,2,2,0,2,0,0,2,2,0,0,2,0,2,2,0,0,2,2,0,2,0,0,2,2,0,0,2,0,2,2,0,0,2,2,0,2,0,0,2,2,0,0,
2,0,2,2,0,0,2,2,0,2,0,0,2,2,0,0,2}; // 3rd iteration rec. phase
static int ph34[128] = {0,2, 2,0, 0,2,2,0, 2,0,0,2,2,0,0,2, 2,0,0,2,2,0,0,2,0,2,2,0,0,2,2,0,
0,2,2,0,0,2,2,0,2,0,0,2,2,0,0,2,2,0,0,2,2,0,0,2,0,2,2,0,0,2,2,0,
0,2,2,0,0,2,2,0,2,0,0,2,2,0,0,2,2,0,0,2,2,0,0,2,0,2,2,0,0,2,2,0,0,2,2,0,0,2,2,0,2,0,0,2,2,0,0,
2,2,0,0,2,2,0,0,2,0,2,2,0,0,2,2,0}; // 4th iteration rec. phase
static int ph35[128] = {0,2, 2,0, 0,2,2,0, 2,0,0,2,2,0,0,2, 2,0,0,2,2,0,0,2,0,2,2,0,0,2,2,0,
0,2,2,0,0,2,2,0,2,0,0,2,2,0,0,2,2,0,0,2,2,0,0,2,0,2,2,0,0,2,2,0,
2,0,0,2,2,0,0,2,0,2,2,0,0,2,2,0,0,2,2,0,0,2,2,0,2,0,0,2,2,0,0,2,2,0,0,2,2,0,0,2,0,2,2,0,0,2,2,
0,0,2,2,0,0,2,2,0,2,0,0,2,2,0,0,2}; // 5th iteration rec. phase


// 120 degree step size phase cycling
```

```
static int  ph1_3s[72] = {0,0,0,0,0,0, 0,0,0,0,0,0, 0,0,0,0,0,0, 0,0,0,0,0,0, 0,0,0,0,0,0,
0,0,0,0,0,0, 0,0,0,0,0,0, 0,0,0,0,0,0, 0,0,0,0,0,0, 0,0,0,0,0,0, 0,0,0,0,0,0, 0,0,0,0,0,0};
  // initial 90
static int ph2_3s[72] = {0,0,0,0,0,0, 0,0,0,0,0,0, 0,0,0,0,0,0, 0,0,0,0,0,0, 0,0,0,0,0,0,
0,0,0,0,0,0, 0,0,0,0,0,0, 0,0,0,0,0,0, 0,0,0,0,0,0, 0,0,0,0,0,0, 0,0,0,0,0,0, 0,0,0,0,0,0};
  // hard 180

static int ph21_3s[72] = {0,1,0,1,0,1, 0,1,0,1,0,1, 0,1,0,1,0,1, 0,1,0,1,0,1, 0,1,0,1,0,1,
0,1,0,1,0,1, 0,1,0,1,0,1, 0,1,0,1,0,1, 0,1,0,1,0,1, 0,1,0,1,0,1, 0,1,0,1,0,1, 0,1,0,1,0,1};
  // 1st iteration sel. 180
static int ph22_3s[72] = {0,0,1,1,2,2, 0,0,1,1,2,2, 0,0,1,1,2,2, 0,0,1,1,2,2, 0,0,1,1,2,2,
0,0,1,1,2,2, 0,0,1,1,2,2, 0,0,1,1,2,2, 0,0,1,1,2,2, 0,0,1,1,2,2, 0,0,1,1,2,2, 0,0,1,1,2,2};
  // 2nd iteration sel. 180
static int ph23_3s[72] = {0,0,0,0,0,0, 1,1,1,1,1,1, 0,0,0,0,0,0, 1,1,1,1,1,1, 0,0,0,0,0,0,
1,1,1,1,1,1, 0,0,0,0,0,0, 1,1,1,1,1,1, 0,0,0,0,0,0, 1,1,1,1,1,1, 0,0,0,0,0,0, 1,1,1,1,1,1};
  // 3rd iteration sel. 180
static int ph24_3s[72] = {0,0,0,0,0,0, 0,0,0,0,0,0, 1,1,1,1,1,1, 1,1,1,1,1,1, 2,2,2,2,2,2,
2,2,2,2,2,2, 0,0,0,0,0,0, 0,0,0,0,0,0, 1,1,1,1,1,1, 1,1,1,1,1,1, 2,2,2,2,2,2, 2,2,2,2,2,2};
  // 4th iteration sel. 180
static int ph25_3s[72] = {0,0,0,0,0,0, 0,0,0,0,0,0, 0,0,0,0,0,0, 0,0,0,0,0,0, 0,0,0,0,0,0,
0,0,0,0,0,0, 1,1,1,1,1,1, 1,1,1,1,1,1, 1,1,1,1,1,1, 1,1,1,1,1,1, 1,1,1,1,1,1, 1,1,1,1,1,1};
  // 5th iteration sel. 180

static int ph31_3s[72] = {0,2,0,2,0,2, 0,2,0,2,0,2, 0,2,0,2,0,2, 0,2,0,2,0,2, 0,2,0,2,0,2,
0,2,0,2,0,2, 0,2,0,2,0,2, 0,2,0,2,0,2, 0,2,0,2,0,2, 0,2,0,2,0,2, 0,2,0,2,0,2, 0,2,0,2,0,2};
  // 1st iteration rec. phase
static int ph32_3s[72] = {0,2,2,1,1,0, 0,2,2,1,1,0, 0,2,2,1,1,0, 0,2,2,1,1,0, 0,2,2,1,1,0,
0,2,2,1,1,0, 0,2,2,1,1,0, 0,2,2,1,1,0, 0,2,2,1,1,0, 0,2,2,1,1,0, 0,2,2,1,1,0, 0,2,2,1,1,0};
  // 2nd iteration rec. phase
static int ph33_3s[72] = {0,2,2,1,1,0, 2,1,1,0,0,2, 0,2,2,1,1,0, 2,1,1,0,0,2, 0,2,2,1,1,0,
2,1,1,0,0,2, 0,2,2,1,1,0, 2,1,1,0,0,2, 0,2,2,1,1,0, 2,1,1,0,0,2, 0,2,2,1,1,0, 2,1,1,0,0,2};
  // 3rd iteration rec. phase
static int ph34_3s[72] = {0,2,2,1,1,0, 2,1,1,0,0,2, 2,1,1,0,0,2, 1,0,0,2,2,1, 1,0,0,2,2,1,
0,2,2,1,1,0, 0,2,2,1,1,0, 2,1,1,0,0,2, 2,1,1,0,0,2, 1,0,0,2,2,1, 1,0,0,2,2,1, 0,2,2,1,1,0};
  // 4th iteration rec. phase
static int ph35_3s[72] = {0,2,2,1,1,0, 2,1,1,0,0,2, 2,1,1,0,0,2, 1,0,0,2,2,1, 1,0,0,2,2,1,
0,2,2,1,1,0, 2,1,1,0,0,2, 1,0,0,2,2,1, 1,0,0,2,2,1, 0,2,2,1,1,0, 0,2,2,1,1,0, 2,1,1,0,0,2};
  // 5th iteration rec. phase


pulsesequence() {

double
  rof3 = getval("rof3"),
  tpwr = getval("tpwr"),
  tpwrf = getval("tpwrf"),
  pw = getval("pw"),
  pp = 2.0*pw,
  pplvl = tpwr,
  pplvlf = getval("pplvlf"),
  pw180_a = getval("pw180_a"),
  pwr180_a = getval("pwr180_a"),
  hsgt = getval("hsgt"),
  hsglvl = getval("hsglvl"),
  gstab = getval("gstab"),
  gt1 = getval("gt1"),
  gzlvl1 = getval("gzlvl1"),
  gt2 = getval("gt2"),
  gzlvl2 = getval("gzlvl2"),
  gzlvl7 = getval("gzlvl7"),
  kp_pfgtc = getval("kp_pfgtc"),
  sw1 = getval("sw1"),
  droppts = getval("droppts"),
  tau_E = getval("tau_E"),
  phstepsize = getval("phstepsize"),
  pw_BIP = getval("pw_BIP"),
  pwr_BIP = getval("pwr_BIP");

int
  kpph = getval("kpph"),
  numreps = getval("numreps"),
  ph_index = getval("ph_index"),
  min_nt = 0;

char
  sspul[MAXSTR],
  lkgate_flg[MAXSTR],
```

```
  shp_a[MAXSTR],
  shp_BIP[MAXSTR],
  BIPflg[MAXSTR],
  altgradflg[MAXSTR];
  getstr("sspul",sspul);
  getstr("lkgate_flg",lkgate_flg);
  getstr("shp_a",shp_a);
  getstr("shp_BIP",shp_BIP);
  getstr("BIPflg",BIPflg);
  getstr("altgradflg",altgradflg);


// If band-selective experiment set no pfgtc
if (gzlvl7==0.0) kp_pfgtc=0.0;

// Only write to the FID when acquiring data
setacqmode(WACQ|NZ);

// Error messages
if ((kpph < 0) || (kpph > 128))       {abort_message("Number of steps for phase cycling is
incorrect - kpph invalid");}
if ((fmod(droppts,2))!=0.0)        {abort_message("droppts must be an integer multiple of
2");}
if ((0.25/sw1-gt1-gstab) < 0.0)       {abort_message("Chunk duration is too short to
accomodate gt1 and gstab");}
if ((fmod(sw/sw1,1)) !=0.0)        {abort_message("sw1 must be an integer submultiple of
sw");}
if (gzlvl1 > 20000)            {abort_message("gzlvl1 cannot exceed 20000");}
if (gzlvl2 > 20000)            {abort_message("gzlvl2 cannot exceed 20000");}
if (numreps > 5)             {abort_message("numreps cannot exceed 5");}
if (numreps < 1)             {abort_message("numreps cannot be less than 1");}
if ((rof2 != rof3) || (rof2 != getval("alfa"))) {abort_message("rof2, rof3 and alfa must be
equal. Check values.");}

// Adjust rof3 to account for the "rof3 + 50 ns" timing of startacq() versus "rof3" for
rcvron()
rof3 = rof2 - 50e-9;

// Phase cycling
if (phstepsize == 120.0) {

  obsstepsize(phstepsize);

  if (kpph == 0) {

    settable(t1,72,ph1_3s);
    settable(t2,72,ph2_3s);

    settable(t21,72,ph21_3s);
    settable(t22,72,ph22_3s);
    settable(t23,72,ph23_3s);
    settable(t24,72,ph24_3s);
    settable(t25,72,ph25_3s);

    settable(t31,72,ph31_3s);
    settable(t32,72,ph32_3s);
    settable(t33,72,ph33_3s);
    settable(t34,72,ph34_3s);
    settable(t35,72,ph35_3s);

  } else {

    settable(t1,kpph,ph1_3s);
    settable(t2,kpph,ph2_3s);

    settable(t21,kpph,ph21_3s);
    settable(t22,kpph,ph22_3s);
    settable(t23,kpph,ph23_3s);
    settable(t24,kpph,ph24_3s);
    settable(t25,kpph,ph25_3s);

    settable(t31,kpph,ph31_3s);
    settable(t32,kpph,ph32_3s);
    settable(t33,kpph,ph33_3s);
    settable(t34,kpph,ph34_3s);
    settable(t35,kpph,ph35_3s);
```

```
  }

} else {

  if (kpph == 0) {

    settable(t1,128,ph1);
    settable(t2,128,ph2);

    settable(t21,128,ph21);
    settable(t22,128,ph22);
    settable(t23,128,ph23);
    settable(t24,128,ph24);
    settable(t25,128,ph25);

    settable(t31,128,ph31);
    settable(t32,128,ph32);
    settable(t33,128,ph33);
    settable(t34,128,ph34);
    settable(t35,128,ph35);

  } else {

    settable(t1,kpph,ph1);
    settable(t2,kpph,ph2);

    settable(t21,kpph,ph21);
    settable(t22,kpph,ph22);
    settable(t23,kpph,ph23);
    settable(t24,kpph,ph24);
    settable(t25,kpph,ph25);

    settable(t31,kpph,ph31);
    settable(t32,kpph,ph32);
    settable(t33,kpph,ph33);
    settable(t34,kpph,ph34);
    settable(t35,kpph,ph35);

  }
}
/*
// Check nt is correctly set
if (phstepsize == 120.0) {
  if (numreps == 1) {min_nt = 2;}
  if (numreps == 2) {min_nt = 6;}
  if (numreps == 3) {min_nt = 12;}
  if (numreps == 4) {min_nt = 36;}
  if (numreps == 5) {min_nt = 72;}
} else {
  if (numreps == 1) {min_nt = 2;}
  if (numreps == 2) {min_nt = 8;}
  if (numreps == 3) {min_nt = 16;}
  if (numreps == 4) {min_nt = 64;}
  if (numreps == 5) {min_nt = 128;}
}
if (nt < min_nt)        {abort_message("nt is less than the minimum number of phase cycle
steps");}
if (fmod(nt,min_nt) != 0.0) {abort_message("The current value of nt results in a partial phase
cycle");}
*/

// Assign real-time variables from phase tables
if (ph_index == 0) {

  sub(ct, ssctr, v20);

  // Asign phases for hard pulses
  getelem(t1, v20, v1);
  getelem(t2, v20, v2);

  if (numreps == 1) {
    getelem(t21, v20, v21); // 1st iteration sel. 180 phase (2-step)
    getelem(t31, v20, oph); // rec. phase
  }
  if (numreps == 2) {
    getelem(t22, v20, v21); // 1st iteration sel. 180 phase (4-step)
    getelem(t21, v20, v22);  // 2nd iteration sel. 180 phase (2-step)
```

```
    getelem(t32, v20, oph); // rec. phase
  }
  if (numreps == 3) {
    getelem(t21, v20, v21); // 1st iteration sel. 180 phase (2-step)
    getelem(t22, v20, v22);  // 2nd iteration sel. 180 phase (4-step)
    getelem(t23, v20, v23);  // 3rd iteration sel. 180 phase (2-step)
    getelem(t33, v20, oph); // rec. phase
  }
  if (numreps == 4) {
    getelem(t22, v20, v21); // 1st iteration sel. 180 phase (4-step)
    getelem(t21, v20, v22);  // 2nd iteration sel. 180 phase (2-step)
    getelem(t24, v20, v23);  // 3rd iteration sel. 180 phase (4-step)
    getelem(t23, v20, v24); // 4th iteration sel. 180 phase (2-step)
    getelem(t34, v20, oph); // rec. phase
  }
  if (numreps == 5) {
    getelem(t21, v20, v21); // 1st iteration sel. 180 phase (2-step)
    getelem(t22, v20, v22);  // 2nd iteration sel. 180 phase (4-step)
    getelem(t23, v20, v23);  // 3rd iteration sel. 180 phase (2-step)
    getelem(t24, v20, v24);  // 4th iteration sel. 180 phase (4-step)
    getelem(t25, v20, v25);  // 5th iteration sel. 180 phase (2-step)
    getelem(t35, v20, oph); // rec. phase
  }

} else {

  initval(ph_index-1,v19);

  // Asign phases for hard pulses
  getelem(t1, v19, v1);
  getelem(t2, v19, v2);

  if (numreps == 1) {
    getelem(t21, v19, v21); // 1st iteration sel. 180 phase (2-step)
    getelem(t31, v19, oph); // rec. phase
  }
  if (numreps == 2) {
    getelem(t22, v19, v21); // 1st iteration sel. 180 phase (4-step)
    getelem(t21, v19, v22);  // 2nd iteration sel. 180 phase (2-step)
    getelem(t32, v19, oph); // rec. phase
  }
  if (numreps == 3) {
    getelem(t21, v19, v21); // 1st iteration sel. 180 phase (2-step)
    getelem(t22, v19, v22);  // 2nd iteration sel. 180 phase (4-step)
    getelem(t23, v19, v23);  // 3rd iteration sel. 180 phase (2-step)
    getelem(t33, v19, oph); // rec. phase
  }
  if (numreps == 4) {
    getelem(t22, v19, v21); // 1st iteration sel. 180 phase (4-step)
    getelem(t21, v19, v22);  // 2nd iteration sel. 180 phase (2-step)
    getelem(t24, v19, v23);  // 3rd iteration sel. 180 phase (4-step)
    getelem(t23, v19, v24); // 4th iteration sel. 180 phase (2-step)
    getelem(t34, v19, oph); // rec. phase
  }
  if (numreps == 5) {
    getelem(t21, v19, v21); // 1st iteration sel. 180 phase (2-step)
    getelem(t22, v19, v22);  // 2nd iteration sel. 180 phase (4-step)
    getelem(t23, v19, v23);  // 3rd iteration sel. 180 phase (2-step)
    getelem(t24, v19, v24);  // 4th iteration sel. 180 phase (4-step)
    getelem(t25, v19, v25);  // 5th iteration sel. 180 phase (2-step)
    getelem(t35, v19, oph); // rec. phase
  }
}


// Define timings
double   tau_C = 0.25/sw1,
    tau_G = gt1 + gstab + rof1,
    tau_H = tau_C - tau_G,
    tau_D = (droppts/sw) + rof2,
    tau_S = tau_C + tau_D;

// Determine if odd or even transient for alternating gradients
mod2(ct,v9);
```

```
/* ACTUAL PULSE SEQUENCE ------------------------------------------------- */

// Relaxation delay (and optional sspul)
status(A);
  txphase(zero);
  xmtrphase(zero);
    obsoffset(tof);
  obspower(tpwr);
  obspwrf(tpwrf);
  delay(0.001);
  if (sspul[0]=='y') {
    if ( (lkgate_flg[0] == 'y') || (lkgate_flg[0] == 'k') )  lk_hold();
    delay(0.001);
    zgradpulse(hsglvl,hsgt);
    delay(gstab);
    rgpulse(pw,zero,rof1,rof1);
    zgradpulse(hsglvl,hsgt);
    delay(0.05);
  }
  if ( (lkgate_flg[0] == 'y') || (lkgate_flg[0] == 'k') )  lk_sample();
  delay(d1);
  if ( (lkgate_flg[0] == 'y') || (lkgate_flg[0] == 'k') )  lk_hold();
  delay(0.001);


// Pulse sequence proper
status(B);

// 90 PULSE
if (phstepsize == 120.0) {
  sub(three,oph,v1);
  add(oph,one,oph);
  obsstepsize(30.0);
  xmtrphase(v1);
  rgpulse(pw,zero,rof1,0.0);
  xmtrphase(zero);
  obsstepsize(phstepsize);
} else {
  rgpulse(pw,v1,rof1,0.0);
}

// "FIFTH" REPETITION
if (numreps > 4) {

  // HARD 180

    // Set pulse power for hard 180
    obspower(pplvl);
    if (tpwrf!=pplvlf) obspwrf(pplvlf);

    // CTP grad
    // Opposite gradient polarity for consecutive repetitions
    delay(tau_H);

    ifzero(v9); zgradpulse(gzlvl1,gt1);
    elsenz(v9);  zgradpulse(-gzlvl1,gt1);
    endif(v9);

    delay(gstab);

    // Hard 180
    if (BIPflg[0] == 'y') {
      shaped_pulse(shp_BIP,pw_BIP,v2,rof1,rof1);
    } else {
      rgpulse(pp,v2,rof1,rof1);
    }

    // Set selective pulse power
    obspower(pwr180_a);
    if (tpwrf!=pplvlf) obspwrf(4095.0);

    // CTP grad
    ifzero(v9); zgradpulse(gzlvl1,gt1);
    elsenz(v9);  zgradpulse(-gzlvl1,gt1);
    endif(v9);

    delay(gstab + tau_H);
```

```
  // SELECTIVE 180

    // Set phase shift for 120 degree step size phase cycling
    if (phstepsize == 120.0) xmtrphase(v25);

    // CTP grad
    // shift the delay from around the hard pulse in the normal sequence
    // to around the selective pulse in the repeating block
    delay(tau_S + gstab + tau_E);


    // CTP grad
    ifzero(v9); zgradpulse(gzlvl2,gt2);
    elsenz(v9); zgradpulse(-gzlvl2,gt2);
    endif(v9);

    delay(gstab);


    // Turn on grad for ZS experiment
    if (gzlvl7>0.0) {
      rgradient('z',gzlvl7);
      delay(kp_pfgtc);
    }

    // Selective 180
    if (phstepsize == 120.0) {
      shaped_pulse(shp_a,pw180_a,zero,rof1,rof1);
      xmtrphase(zero);
    } else {
      shaped_pulse(shp_a,pw180_a,v25,rof1,rof1);
    }

    // Turn off grad for ZS experiment
    if (gzlvl7>0.0) {
      rgradient('z',0.0);
      delay(kp_pfgtc);
    }

    // CTP grad
    delay(gstab);

    ifzero(v9); zgradpulse(gzlvl2,gt2);
    elsenz(v9); zgradpulse(-gzlvl2,gt2);
    endif(v9);

    delay(gstab + tau_S + tau_E);

}

// "FOURTH" REPETITION
if (numreps > 3) {

  // HARD 180

    // Set pulse power for hard 180
    obspower(pplvl);
    if (tpwrf!=pplvlf) obspwrf(pplvlf);

    // CTP grad
    // Opposite gradient polarity for consecutive repetitions
    delay(tau_H);

    if (altgradflg[0] == 'y') {
      ifzero(v9); zgradpulse(-gzlvl1,gt1);
      elsenz(v9);  zgradpulse(gzlvl1,gt1);
      endif(v9);
    } else {
      ifzero(v9); zgradpulse(gzlvl1,gt1);
      elsenz(v9);  zgradpulse(-gzlvl1,gt1);
      endif(v9);
    }


    delay(gstab);
```

```
  // Hard 180
  if (BIPflg[0] == 'y') {
    shaped_pulse(shp_BIP,pw_BIP,v2,rof1,rof1);
  } else {
    rgpulse(pp,v2,rof1,rof1);
  }

  // Set selective pulse power
  obspower(pwr180_a);
  if (tpwrf!=pplvlf) obspwrf(4095.0);

  // CTP grad
  if (altgradflg[0] == 'y') {
    ifzero(v9); zgradpulse(-gzlvl1,gt1);
    elsenz(v9);  zgradpulse(gzlvl1,gt1);
    endif(v9);
  } else {
    ifzero(v9); zgradpulse(gzlvl1,gt1);
    elsenz(v9);  zgradpulse(-gzlvl1,gt1);
    endif(v9);
  }

  delay(gstab + tau_H);


// SELECTIVE 180

  // Set phase shift for 120 degree step size phase cycling
  if (phstepsize == 120.0) xmtrphase(v24);

  // CTP grad
  // shift the delay from around the hard pulse in the normal sequence
  // to around the selective pulse in the repeating block
  delay(tau_S + gstab + tau_E);


  // CTP grad
  if (altgradflg[0] == 'y') {
    ifzero(v9); zgradpulse(-gzlvl2,gt2);
    elsenz(v9); zgradpulse(gzlvl2,gt2);
    endif(v9);
  } else {
    ifzero(v9); zgradpulse(gzlvl2,gt2);
    elsenz(v9); zgradpulse(-gzlvl2,gt2);
    endif(v9);
  }

  delay(gstab);


  // Turn on grad for ZS experiment
  if (gzlvl7>0.0) {
    rgradient('z',gzlvl7);
    delay(kp_pfgtc);
  }

  // Selective 180
  if (phstepsize == 120.0) {
    shaped_pulse(shp_a,pw180_a,zero,rof1,rof1);
    xmtrphase(zero);
  } else {
    shaped_pulse(shp_a,pw180_a,v24,rof1,rof1);
  }

  // Turn off grad for ZS experiment
  if (gzlvl7>0.0) {
    rgradient('z',0.0);
    delay(kp_pfgtc);
  }

  // CTP grad
  delay(gstab);

  if (altgradflg[0] == 'y') {
    ifzero(v9); zgradpulse(-gzlvl2,gt2);
    elsenz(v9); zgradpulse(gzlvl2,gt2);
```

```
      endif(v9);
    } else {
      ifzero(v9); zgradpulse(gzlvl2,gt2);
      elsenz(v9); zgradpulse(-gzlvl2,gt2);
      endif(v9);
    }

    delay(gstab + tau_S + tau_E);

}


// "THIRD" REPETITION
if (numreps > 2) {

  // HARD 180

    // Set pulse power for hard 180
    obspower(pplvl);
    if (tpwrf!=pplvlf) obspwrf(pplvlf);

    // CTP grad
    // Opposite gradient polarity for consecutive repetitions
    delay(tau_H);

    ifzero(v9); zgradpulse(gzlvl1,gt1);
    elsenz(v9);  zgradpulse(-gzlvl1,gt1);
    endif(v9);

    delay(gstab);

    // Hard 180
    if (BIPflg[0] == 'y') {
      shaped_pulse(shp_BIP,pw_BIP,v2,rof1,rof1);
    } else {
      rgpulse(pp,v2,rof1,rof1);
    }

    // Set selective pulse power
    obspower(pwr180_a);
    if (tpwrf!=pplvlf) obspwrf(4095.0);

    // CTP grad
    ifzero(v9); zgradpulse(gzlvl1,gt1);
    elsenz(v9);  zgradpulse(-gzlvl1,gt1);
    endif(v9);

    delay(gstab + tau_H);


  // SELECTIVE 180

    // Set phase shift for 120 degree step size phase cycling
    if (phstepsize == 120.0) xmtrphase(v23);

    // CTP grad
    // shift the delay from around the hard pulse in the normal sequence
    // to around the selective pulse in the repeating block
    delay(tau_S + gstab + tau_E);


    // CTP grad
    ifzero(v9); zgradpulse(gzlvl2,gt2);
    elsenz(v9); zgradpulse(-gzlvl2,gt2);
    endif(v9);

    delay(gstab);


    // Turn on grad for ZS experiment
    if (gzlvl7>0.0) {
      rgradient('z',gzlvl7);
      delay(kp_pfgtc);
    }

    // Selective 180
    if (phstepsize == 120.0) {
```

```
      shaped_pulse(shp_a,pw180_a,zero,rof1,rof1);
      xmtrphase(zero);
    } else {
      shaped_pulse(shp_a,pw180_a,v23,rof1,rof1);
    }

    // Turn off grad for ZS experiment
    if (gzlvl7>0.0) {
      rgradient('z',0.0);
      delay(kp_pfgtc);
    }

    // CTP grad
    delay(gstab);

    ifzero(v9); zgradpulse(gzlvl2,gt2);
    elsenz(v9); zgradpulse(-gzlvl2,gt2);
    endif(v9);

    delay(gstab + tau_S + tau_E);

}


// "SECOND" REPETITION
if (numreps > 1) {

  // HARD 180

    // Set pulse power for hard 180
    obspower(pplvl);
    if (tpwrf!=pplvlf) obspwrf(pplvlf);

    // CTP grad
    delay(tau_H);

    if (altgradflg[0] == 'y') {
      ifzero(v9); zgradpulse(-gzlvl1,gt1);
      elsenz(v9);  zgradpulse(gzlvl1,gt1);
      endif(v9);
    } else {
      ifzero(v9); zgradpulse(gzlvl1,gt1);
      elsenz(v9);  zgradpulse(-gzlvl1,gt1);
      endif(v9);
    }

    delay(gstab);

    // Hard 180
    if (BIPflg[0] == 'y') {
      shaped_pulse(shp_BIP,pw_BIP,v2,rof1,rof1);
    } else {
      rgpulse(pp,v2,rof1,rof1);
    }

    // Set selective pulse power
    obspower(pwr180_a);
    if (tpwrf!=pplvlf) obspwrf(4095.0);

    // CTP grad
    if (altgradflg[0] == 'y') {
      ifzero(v9); zgradpulse(-gzlvl1,gt1);
      elsenz(v9);  zgradpulse(gzlvl1,gt1);
      endif(v9);
    } else {
      ifzero(v9); zgradpulse(gzlvl1,gt1);
      elsenz(v9);  zgradpulse(-gzlvl1,gt1);
      endif(v9);
    }
    delay(gstab + tau_H);


  // SELECTIVE 180

    // Set phase shift for 120 degree step size phase cycling
    if (phstepsize == 120.0) xmtrphase(v22);
```

```
    // Extra delay
    if (numreps % 2 == 0) {
      delay(tau_D + tau_E);
    } else {
      delay(tau_S + tau_E);
    }

    // CTP grad
    delay(gstab);
    if (altgradflg[0] == 'y') {
      ifzero(v9); zgradpulse(-gzlvl2,gt2);
      elsenz(v9); zgradpulse(gzlvl2,gt2);
      endif(v9);
    } else {
      ifzero(v9); zgradpulse(gzlvl2,gt2);
      elsenz(v9); zgradpulse(-gzlvl2,gt2);
      endif(v9);
    }
    delay(gstab);

    // Turn on grad for ZS experiment
    if (gzlvl7>0.0) {
      rgradient('z',gzlvl7);
      delay(kp_pfgtc);
    }

    // Selective 180
    if (phstepsize == 120.0) {
      shaped_pulse(shp_a,pw180_a,zero,rof1,rof1);
      xmtrphase(zero);
    } else {
      shaped_pulse(shp_a,pw180_a,v22,rof1,rof1);
    }

    // Turn off grad for ZS experiment
    if (gzlvl7>0.0) {
      rgradient('z',0.0);
      delay(kp_pfgtc);
    }

    // CTP grad
    delay(gstab);
    if (altgradflg[0] == 'y') {
      ifzero(v9); zgradpulse(-gzlvl2,gt2);
      elsenz(v9); zgradpulse(gzlvl2,gt2);
      endif(v9);
    } else {
      ifzero(v9); zgradpulse(gzlvl2,gt2);
      elsenz(v9); zgradpulse(-gzlvl2,gt2);
      endif(v9);
    }
    delay(gstab);

    // Extra delay
    if (numreps % 2 == 0) {
      delay(tau_D + tau_E);
    } else {
      delay(tau_S + tau_E);
    }
}

// "FIRST" REPETITION

// 1st t1/2 evolution
delay(d2/2.0);

// Set pulse power for hard 180
obspower(pplvl);
if (tpwrf!=pplvlf) obspwrf(pplvlf);

// CTP grad
if (numreps % 2 == 0) {
  delay(tau_C);
}
delay(tau_H);
ifzero(v9); zgradpulse(gzlvl1,gt1);
elsenz(v9); zgradpulse(-gzlvl1,gt1);
```

```
endif(v9);
delay(gstab);

// Hard 180
if (BIPflg[0] == 'y') {
  shaped_pulse(shp_BIP,pw_BIP,v2,rof1,rof1);
} else {
  rgpulse(pp,v2,rof1,rof1);
}

// Set selective pulse power
obspower(pwr180_a);
if (tpwrf!=pplvlf) obspwrf(4095.0);

// CTP grad
ifzero(v9); zgradpulse(gzlvl1,gt1);
elsenz(v9); zgradpulse(-gzlvl1,gt1);
endif(v9);

delay(gstab + tau_H);
if (numreps % 2 == 0) {
  delay(tau_C);
}

// Set phase shift for 120 degree step size phase cycling
if (phstepsize == 120.0) xmtrphase(v21);

// Drop points
delay(tau_D);

// CTP grad
delay(gstab + tau_C + tau_E);
ifzero(v9); zgradpulse(gzlvl2,gt2);
elsenz(v9); zgradpulse(-gzlvl2,gt2);
endif(v9);
delay(gstab);

// Turn on grad for ZS experiment
if (gzlvl7>0.0) {
  rgradient('z',gzlvl7);
  delay(kp_pfgtc);
}

// Selective 180
if (phstepsize == 120.0) {
  shaped_pulse(shp_a,pw180_a,zero,rof1,rof1);
  xmtrphase(zero);
} else {
  shaped_pulse(shp_a,pw180_a,v21,rof1,rof1);
}

// Turn off grad for ZS experiment
if (gzlvl7>0.0) {
  rgradient('z',0.0);
  delay(kp_pfgtc);
}

// CTP grad
// if/else to have the longer delay after the gradient pulse
delay(gstab);
ifzero(v9); zgradpulse(gzlvl2,gt2);
elsenz(v9); zgradpulse(-gzlvl2,gt2);
endif(v9);
delay(gstab + tau_C + tau_E);

// 2nd t1/2 evolution
delay(d2/2.0);

// Set phase shift for 120 degree step size phase cycling
if (phstepsize == 120.0) xmtrphase(v31);

// ACQUISITION
status(C);
startacq(getval("alfa"));
  sample(np/2.0/sw);
  recoff();
  endacq();
```

```
delay(0.001);
  if ( (lkgate_flg[0] == 'y') || (lkgate_flg[0] == 'k') )  lk_sample();
delay(0.001);
}
```

S11.2. Varian/Agilent semi-real-time acquisition mode pulse sequence code

```
/* UoM_EXQUISITE_srt -------------------------------------------------------

 EXQUISITE band-selective/Zangger-Sterk pure shift NMR experiment with
 semi-real-time acquisition.

 Howard M. Foster
 The University of Manchester
 Manchester NMR Methodology Group
 v1.0 27/07/2023
--------------------------------------------------------------------------*/


#include <standard.h>


//phase cycle on all pulses
//v10;v11 reserved for the real-time loop
//v12;v13 reserved for ifzero PFGs

static int  ph1[128] = {0,0, 0,0, 0,0,0,0, 0,0,0,0,0,0,0,0, 0,0,0,0,0,0,0,0,0,0,0,0,0,0,0,0,
0,0,0,0,0,0,0,0,0,0,0,0,0,0,0,0,0,0,0,0,0,0,0,0,0,0,0,0,0,0,0,0,
0,0,0,0,0,0,0,0,0,0,0,0,0,0,0,0,0,0,0,0,0,0,0,0,0,0,0,0,0,0,0,0,0,0,0,0,0,0,0,0,0,0,0,0,0,0,0,
0,0,0,0,0,0,0,0,0,0,0,0,0,0,0,0,0}; // initial 90
static int ph2[128]  = {0,0, 0,0, 0,0,0,0, 0,0,0,0,0,0,0,0, 0,0,0,0,0,0,0,0,0,0,0,0,0,0,0,0,
0,0,0,0,0,0,0,0,0,0,0,0,0,0,0,0,0,0,0,0,0,0,0,0,0,0,0,0,0,0,0,0,
0,0,0,0,0,0,0,0,0,0,0,0,0,0,0,0,0,0,0,0,0,0,0,0,0,0,0,0,0,0,0,0,0,0,0,0,0,0,0,0,0,0,0,0,0,0,0,
0,0,0,0,0,0,0,0,0,0,0,0,0,0,0,0,0}; // hard 180

static int ph21[128] = {0,1, 0,1, 0,1,0,1, 0,1,0,1,0,1,0,1, 0,1,0,1,0,1,0,1,0,1,0,1,0,1,0,1,
0,1,0,1,0,1,0,1,0,1,0,1,0,1,0,1,0,1,0,1,0,1,0,1,0,1,0,1,0,1,0,1,
0,1,0,1,0,1,0,1,0,1,0,1,0,1,0,1,0,1,0,1,0,1,0,1,0,1,0,1,0,1,0,1,0,1,0,1,0,1,0,1,0,1,0,1,0,1,0,
1,0,1,0,1,0,1,0,1,0,1,0,1,0,1,0,1}; // 1st iteration sel. 180
static int ph22[128] = {0,0, 1,1, 2,2,3,3, 0,0,1,1,2,2,3,3, 0,0,1,1,2,2,3,3,0,0,1,1,2,2,3,3,
0,0,1,1,2,2,3,3,0,0,1,1,2,2,3,3,0,0,1,1,2,2,3,3,0,0,1,1,2,2,3,3,
0,0,1,1,2,2,3,3,0,0,1,1,2,2,3,3,0,0,1,1,2,2,3,3,0,0,1,1,2,2,3,3,0,0,1,1,2,2,3,3,0,0,1,1,2,2,3,
3,0,0,1,1,2,2,3,3,0,0,1,1,2,2,3,3}; // 2nd iteration sel. 180
static int ph23[128] = {0,0, 0,0, 0,0,0,0, 1,1,1,1,1,1,1,1, 0,0,0,0,0,0,0,0,1,1,1,1,1,1,1,1,
0,0,0,0,0,0,0,0,1,1,1,1,1,1,1,1,0,0,0,0,0,0,0,0,1,1,1,1,1,1,1,1,
0,0,0,0,0,0,0,0,1,1,1,1,1,1,1,1,0,0,0,0,0,0,0,0,1,1,1,1,1,1,1,1,0,0,0,0,0,0,0,0,1,1,1,1,1,1,1,
1,0,0,0,0,0,0,0,0,1,1,1,1,1,1,1,1}; // 3rd iteration sel. 180
static int ph24[128] = {0,0, 0,0, 0,0,0,0, 0,0,0,0,0,0,0,0, 1,1,1,1,1,1,1,1,1,1,1,1,1,1,1,1,
2,2,2,2,2,2,2,2,2,2,2,2,2,2,2,2,3,3,3,3,3,3,3,3,3,3,3,3,3,3,3,3,
0,0,0,0,0,0,0,0,0,0,0,0,0,0,0,0,1,1,1,1,1,1,1,1,1,1,1,1,1,1,1,1,2,2,2,2,2,2,2,2,2,2,2,2,2,2,2,
2,3,3,3,3,3,3,3,3,3,3,3,3,3,3,3,3}; // 4th iteration sel. 180
static int ph25[128] = {0,0, 0,0, 0,0,0,0, 0,0,0,0,0,0,0,0, 0,0,0,0,0,0,0,0,0,0,0,0,0,0,0,0,
0,0,0,0,0,0,0,0,0,0,0,0,0,0,0,0,0,0,0,0,0,0,0,0,0,0,0,0,0,0,0,0,
1,1,1,1,1,1,1,1,1,1,1,1,1,1,1,1,1,1,1,1,1,1,1,1,1,1,1,1,1,1,1,1,1,1,1,1,1,1,1,1,1,1,1,1,1,1,1,
1,1,1,1,1,1,1,1,1,1,1,1,1,1,1,1,1}; // 5th iteration sel. 180

static int ph31[128] = {0,2, 0,2, 0,2,0,2, 0,2,0,2,0,2,0,2, 0,2,0,2,0,2,0,2,0,2,0,2,0,2,0,2,
0,2,0,2,0,2,0,2,0,2,0,2,0,2,0,2,0,2,0,2,0,2,0,2,0,2,0,2,0,2,0,2,
0,2,0,2,0,2,0,2,0,2,0,2,0,2,0,2,0,2,0,2,0,2,0,2,0,2,0,2,0,2,0,2,0,2,0,2,0,2,0,2,0,2,0,2,0,2,0,
2,0,2,0,2,0,2,0,2,0,2,0,2,0,2,0,2}; // 1st iteration rec. phase
static int ph32[128] = {0,2, 2,0, 0,2,2,0, 0,2,2,0,0,2,2,0, 0,2,2,0,0,2,2,0,0,2,2,0,0,2,2,0,
0,2,2,0,0,2,2,0,0,2,2,0,0,2,2,0,0,2,2,0,0,2,2,0,0,2,2,0,0,2,2,0,
0,2,2,0,0,2,2,0,0,2,2,0,0,2,2,0,0,2,2,0,0,2,2,0,0,2,2,0,0,2,2,0,0,2,2,0,0,2,2,0,0,2,2,0,0,2,2,
0,0,2,2,0,0,2,2,0,0,2,2,0,0,2,2,0}; // 2nd iteration rec. phase
static int ph33[128] = {0,2, 2,0, 0,2,2,0, 2,0,0,2,2,0,0,2, 0,2,2,0,0,2,2,0,2,0,0,2,2,0,0,2,
0,2,2,0,0,2,2,0,2,0,0,2,2,0,0,2,0,2,2,0,0,2,2,0,2,0,0,2,2,0,0,2,
0,2,2,0,0,2,2,0,2,0,0,2,2,0,0,2,0,2,2,0,0,2,2,0,2,0,0,2,2,0,0,2,0,2,2,0,0,2,2,0,2,0,0,2,2,0,0,
2,0,2,2,0,0,2,2,0,2,0,0,2,2,0,0,2}; // 3rd iteration rec. phase
static int ph34[128] = {0,2, 2,0, 0,2,2,0, 2,0,0,2,2,0,0,2, 2,0,0,2,2,0,0,2,0,2,2,0,0,2,2,0,
0,2,2,0,0,2,2,0,2,0,0,2,2,0,0,2,2,0,0,2,2,0,0,2,0,2,2,0,0,2,2,0,
```

```
0,2,2,0,0,2,2,0,2,0,0,2,2,0,0,2,2,0,0,2,2,0,0,2,0,2,2,0,0,2,2,0,0,2,2,0,0,2,2,0,2,0,0,2,2,0,0,
2,2,0,0,2,2,0,0,2,0,2,2,0,0,2,2,0}; // 4th iteration rec. phase
static int ph35[128] = {0,2, 2,0, 0,2,2,0, 2,0,0,2,2,0,0,2, 2,0,0,2,2,0,0,2,0,2,2,0,0,2,2,0,
0,2,2,0,0,2,2,0,2,0,0,2,2,0,0,2,2,0,0,2,2,0,0,2,0,2,2,0,0,2,2,0,
2,0,0,2,2,0,0,2,0,2,2,0,0,2,2,0,0,2,2,0,0,2,2,0,2,0,0,2,2,0,0,2,2,0,0,2,2,0,0,2,0,2,2,0,0,2,2,
0,0,2,2,0,0,2,2,0,2,0,0,2,2,0,0,2}; // 5th iteration rec. phase



//v6 for auto-incremented supercycle additional to the scan-to-scan on v4/v14-v15/v21-v22-v23
//v7 used for small-angle phases
static int
   ph6_m4[4]      = {0,0,2,2},
   ph6_m8[8]      = {0,0,2,2, 0,2,2,0},
   ph6_t5[5]      = {0,5,2,5,0},
   ph6_t7[7]      = {0,7,20,17,20,7,0},
   ph6_t9[9]      = {0,1,12,11,18,11,12,1,0},
   ph6_m16[16]    = {0,0,2,2, 0,2,2,0, 2,2,0,0, 2,0,0,2},
   ph6_m32[32]    = {0,0,2,2, 2,0,0,2, 2,2,0,0, 0,2,2,0,  0,0,0,2, 2,2,0,0, 2,2,2,0, 0,0,2,2},
   ph6_m64[64]    = {0,0,2,2, 2,0,0,2, 2,2,0,0, 0,2,2,0,  0,0,0,2, 2,2,0,0, 2,2,2,0, 0,0,2,2,
  2,2,0,0, 0,2,2,0, 0,0,2,2, 2,0,0,2,  2,2,2,0, 0,0,2,2, 0,0,0,2, 2,2,0,0},
   ph6_t5m4[20]   = {0,5,2,5,0, 0,5,2,5,0, 6,11,8,11,6, 6,11,8,11,6},
     ph6_t5m16[80]  = {0,5,2,5,0, 0,5,2,5,0, 6,11,8,11,6, 6,11,8,11,6, 0,5,2,5,0, 6,11,8,11,6,
6,11,8,11,6, 0,5,2,5,0, 6,11,8,11,6, 6,11,8,11,6, 0,5,2,5,0, 0,5,2,5,0, 6,11,8,11,6, 0,5,2,5,0,
0,5,2,5,0, 6,11,8,11,6},
     ph6_t7m4[28]        =   {0,7,20,17,20,7,0,   0,7,20,17,20,7,0,   12,19,32,29,32,19,12,
12,19,32,29,32,19,12},
     ph6_t7m16[112]    =    {0,7,20,17,20,7,0,    0,7,20,17,20,7,0,    12,19,32,29,32,19,12,
12,19,32,29,32,19,12,    0,7,20,17,20,7,0,    12,19,32,29,32,19,12,    12,19,32,29,32,19,12,
0,7,20,17,20,7,0,     12,19,32,29,32,19,12,     12,19,32,29,32,19,12,     0,7,20,17,20,7,0,
0,7,20,17,20,7,0,      12,19,32,29,32,19,12,      0,7,20,17,20,7,0,      0,7,20,17,20,7,0,
12,19,32,29,32,19,12,},
   ph6_t9m4[36]                 =      {0,1,12,11,18,11,12,1,0,      0,1,12,11,18,11,12,1,0,
12,13,24,23,30,23,24,13,12, 12,13,24,23,30,23,24,13,12},
   ph6[4]        = {0,0,0,0};

pulsesequence() {

double
  rof3 = getval("rof3"),   // should be 2.0us
  tau_p = getval("tau_p"), // tau_p is the total length of the period without acquisition of
data points; must be an integer multiple of 1.0/sw
  tau_r = 0.0,      // calculated by the sequence based on the parameters; the executable delay
before and after the decoupling element; this must accomodate 2*(ACQ_gt1+ACQ_gstab)
  tpwr = getval("tpwr"),
  tpwrf = getval("tpwrf"),
  pw = getval("pw"),
  tau_E = getval("tau_E"),
  pp = 2.0*pw,
  pplvl = tpwr,
  pplvlf = getval("pplvlf"),
  pw180_a = getval("pw180_a"),
  pwr180_a = getval("pwr180_a"),
  pw180_p = getval("pw180_p"),
  pwr180_p = getval("pwr180_p"),
  hsgt = getval("hsgt"),
  hsglvl = getval("hsglvl"),
  gstab = getval("gstab"),
  gt1 = getval("gt1"),
  gzlvl1 = getval("gzlvl1"),
  gt2 = getval("gt2"),
  gzlvl2 = getval("gzlvl2"),
  gzlvl7 = getval("gzlvl7"),
  kp_pfgtc = getval("kp_pfgtc"),  //time correction for PFG group delay
  ACQ_gt1 = getval("ACQ_gt1"),
  ACQ_gzlvl1 = getval("ACQ_gzlvl1"),
  ACQ_gzlvl2 = getval("ACQ_gzlvl2"),
```

```
  ACQ_gzlvl3 = getval("ACQ_gzlvl3"),
  ACQ_gzlvl4 = getval("ACQ_gzlvl4"),
  ACQ_gstab = getval("ACQ_gstab"),
  sw1 = getval("sw1"),     // length of chunk is 1.0/sw1; must be an integer multiple of 1.0/sw
  npoints = getval("npoints"),// no. of complex points acquired in each chunk
  droppts1 = getval("droppts1"),// droppts at the begining of each chunk
  droppts2 = getval("droppts2"),// droppts at the end of each chunk
  pw_BIP = getval("pw_BIP"),
  pwr_BIP = getval("pwr_BIP"),
  cycles=(double)((floor)(np/(2.0*(droppts1+droppts2+npoints)) -1.0 ));//int    truncates    if
needed
  F_initval(cycles,v10);     //loop counters v10(v11)


int
  kpph = getval("kpph"),   // number of steps used from the phase cycle; if zero than all steps
are used
  kp_scyc_len,
  numreps = getval("numreps"),
  ph_index = getval("ph_index"),
  min_nt = 0;

char
  sspul[MAXSTR],
  lkgate_flg[MAXSTR],
  kp_scyc[MAXSTR],
  shp_a[MAXSTR],
  shp_p[MAXSTR],
  shp_BIP[MAXSTR],
  BIPflg[MAXSTR];

  getstr("sspul",sspul);
  getstr("lkgate_flg",lkgate_flg);
  getstr("kp_scyc",kp_scyc);
  getstr("shp_a",shp_a);   //created by macro kp_makePS7 using bw_a/kp_phincr_a
  getstr("shp_p",shp_p);   //created by macro kp_makePS7
  getstr("shp_BIP",shp_BIP);
  getstr("BIPflg",BIPflg);


// Calculate tau_r
tau_r = (tau_p - 2.0*ACQ_gt1-2.0*ACQ_gstab-2.0*rof1-pw180_p)/2.0;

// ACQmode definitions
setacqmode(WACQ|NZ);

// Error messages
if ((kpph<0) || (kpph>64))  {abort_message("Number of steps for phase cycling is incorrect.
Change kpph.");}
if (tau_r < 0)       {abort_message("tau_r  is  less  than  zero,  increase  the  number  of
increments.");}
if (droppts1 != droppts2)  {abort_message("droppts1 and droppts2 should be equal.");}
if (gzlvl1 > 20000)    {abort_message("gzlvl1 cannot exceed 20000");}
if (gzlvl2 > 20000)    {abort_message("gzlvl2 cannot exceed 20000");}
if (numreps > 5)      {abort_message("numreps cannot exceed 5");}
if (numreps < 1)      {abort_message("numreps cannot be less than 1");}
if  ((rof2  !=  rof3)  ||  (rof2  !=  getval("alfa")))  {abort_message("rof2,  rof3  and  alfa  must  be
equal. Check values.");}

// Adjust rof3 to account for the "rof3 + 50 ns" timing of startacq() versus "rof3" for rcvron()
rof3 = rof2 - 50e-9;

// Phase cycling
if (kpph == 0) {

  settable(t1,128,ph1);
  settable(t2,128,ph2);
```

```
  settable(t21,128,ph21);
  settable(t22,128,ph22);
  settable(t23,128,ph23);
  settable(t24,128,ph24);
  settable(t25,128,ph25);

  settable(t31,128,ph31);
  settable(t32,128,ph32);
  settable(t33,128,ph33);
  settable(t34,128,ph34);
  settable(t35,128,ph35);

} else {

  settable(t1,kpph,ph1);
  settable(t2,kpph,ph2);

  settable(t21,kpph,ph21);
  settable(t22,kpph,ph22);
  settable(t23,kpph,ph23);
  settable(t24,kpph,ph24);
  settable(t25,kpph,ph25);

  settable(t31,kpph,ph31);
  settable(t32,kpph,ph32);
  settable(t33,kpph,ph33);
  settable(t34,kpph,ph34);
  settable(t35,kpph,ph35);

}

/*
// Check nt is correctly set
if (phstepsize == 120.0) {
  if (numreps == 1) {min_nt = 2;}
  if (numreps == 2) {min_nt = 6;}
  if (numreps == 3) {min_nt = 12;}
  if (numreps == 4) {min_nt = 36;}
  if (numreps == 5) {min_nt = 72;}
} else {
  if (numreps == 1) {min_nt = 2;}
  if (numreps == 2) {min_nt = 8;}
  if (numreps == 3) {min_nt = 16;}
  if (numreps == 4) {min_nt = 64;}
  if (numreps == 5) {min_nt = 128;}
}
if (nt < min_nt)        {abort_message("nt is less than the minimum number of phase cycle steps");}
if (fmod(nt,min_nt) != 0.0) {abort_message("The current value of nt results in a partial phase cycle");}
*/

// Assign real-time variables from phase tables
if (ph_index == 0) {

  sub(ct, ssctr, v20);

  // Asign phases for hard pulses
  getelem(t1, v20, v1);
  getelem(t2, v20, v2);

  if (numreps == 1) {
    getelem(t21, v20, v21); // 1st iteration sel. 180 phase (2-step)
    getelem(t31, v20, oph); // rec. phase
  }
  if (numreps == 2) {
```

```
    getelem(t22, v20, v21); // 1st iteration sel. 180 phase (4-step)
    getelem(t21, v20, v22);  // 2nd iteration sel. 180 phase (2-step)
    getelem(t32, v20, oph); // rec. phase
  }
  if (numreps == 3) {
    getelem(t21, v20, v21); // 1st iteration sel. 180 phase (2-step)
    getelem(t22, v20, v22);  // 2nd iteration sel. 180 phase (4-step)
    getelem(t23, v20, v23);  // 3rd iteration sel. 180 phase (2-step)
    getelem(t33, v20, oph); // rec. phase
  }
  if (numreps == 4) {
    getelem(t22, v20, v21); // 1st iteration sel. 180 phase  (4-step)
    getelem(t21, v20, v22);  // 2nd iteration sel. 180 phase (2-step)
    getelem(t24, v20, v23);  // 3rd iteration sel. 180 phase (4-step)
    getelem(t23, v20, v24); // 4th iteration sel. 180 phase (2-step)
    getelem(t34, v20, oph); // rec. phase
  }
  if (numreps == 5) {
    getelem(t21, v20, v21); // 1st iteration sel. 180 phase  (2-step)
    getelem(t22, v20, v22);  // 2nd iteration sel. 180 phase (4-step)
    getelem(t23, v20, v23);  // 3rd iteration sel. 180 phase (2-step)
    getelem(t24, v20, v24);  // 4th iteration sel. 180 phase (4-step)
    getelem(t25, v20, v25);  // 5th iteration sel. 180 phase (2-step)
    getelem(t35, v20, oph); // rec. phase
  }

} else {

  initval(ph_index-1,v19);

  // Asign phases for hard pulses
  getelem(t1, v19, v1);
  getelem(t2, v19, v2);

  if (numreps == 1) {
    getelem(t21, v19, v21); // 1st iteration sel. 180 phase (2-step)
    getelem(t31, v19, oph); // rec. phase
  }
  if (numreps == 2) {
    getelem(t22, v19, v21); // 1st iteration sel. 180 phase (4-step)
    getelem(t21, v19, v22);  // 2nd iteration sel. 180 phase (2-step)
    getelem(t32, v19, oph); // rec. phase
  }
  if (numreps == 3) {
    getelem(t21, v19, v21); // 1st iteration sel. 180 phase  (2-step)
    getelem(t22, v19, v22);  // 2nd iteration sel. 180 phase (4-step)
    getelem(t23, v19, v23);  // 3rd iteration sel. 180 phase (2-step)
    getelem(t33, v19, oph); // rec. phase
  }
  if (numreps == 4) {
    getelem(t22, v19, v21); // 1st iteration sel. 180 phase  (4-step)
    getelem(t21, v19, v22);  // 2nd iteration sel. 180 phase (2-step)
    getelem(t24, v19, v23);  // 3rd iteration sel. 180 phase (4-step)
    getelem(t23, v19, v24); // 4th iteration sel. 180 phase (2-step)
    getelem(t34, v19, oph); // rec. phase
  }
  if (numreps == 5) {
    getelem(t21, v19, v21); // 1st iteration sel. 180 phase  (2-step)
    getelem(t22, v19, v22);  // 2nd iteration sel. 180 phase (4-step)
    getelem(t23, v19, v23);  // 3rd iteration sel. 180 phase (2-step)
    getelem(t24, v19, v24);  // 4th iteration sel. 180 phase (4-step)
    getelem(t25, v19, v25);  // 5th iteration sel. 180 phase (2-step)
    getelem(t35, v19, oph); // rec. phase
  }
}

// Chunk to chunk phase sequencing options
```

```
kp_scyc_len = strlen(kp_scyc);
//printf("Length %d",kp_scyc_len);

// No phase sequencing
if (kp_scyc[0] == 'n') {
  settable(t6,4,ph6);
}

// Phase sequencing with m4/m8/t5/t7/t9
if (kp_scyc_len == 2) {

  //m4 phase sequencing
  if ((kp_scyc[0] == 'm') && (kp_scyc[1] == '4')) {
    settable(t6,4,ph6_m4);
  }
  //m8 phase sequencing
  if ((kp_scyc[0] == 'm') && (kp_scyc[1] == '8')) {
    settable(t6,8,ph6_m8);
  }
  //t5 phase sequencing
  if ((kp_scyc[0] == 't') && (kp_scyc[1] == '5')) {
    settable(t6,5,ph6_t5);
    obsstepsize(30.0);
  }
  //t7 phase sequencing
  if ((kp_scyc[0] == 't') && (kp_scyc[1] == '7'))
  {
    settable(t6,7,ph6_t7);
    obsstepsize(15.0);
  }
  //t9 phase sequencing
  if ((kp_scyc[0] == 't') && (kp_scyc[1] == '9')) {
    settable(t6,9,ph6_t9);
    obsstepsize(15.0);
  }
}

// Phase sequencing with m16/m32/m64
if (kp_scyc_len == 3) {

  //m16 phase sequencing
  if ((kp_scyc[0] == 'm') && (kp_scyc[1] == '1'))
  {
  settable(t6,16,ph6_m16);
  }
  //m32 phase sequencing
  if ((kp_scyc[0] == 'm') && (kp_scyc[1] == '3'))
  {
  settable(t6,32,ph6_m32);
  }
  //m64 phase sequencing
  if ((kp_scyc[0] == 'm') && (kp_scyc[1] == '6'))
  {
  settable(t6,64,ph6_m64);
  }
}

// Phase sequencing with t5m4/t7m4/t9m4
if (kp_scyc_len == 4) {

  //t5m4 phase sequencing
  if (kp_scyc[1] == '5') {
    settable(t6,20,ph6_t5m4);
    obsstepsize(30.0);
  }
  //t7m4 phase sequencing
  if (kp_scyc[1] == '7') {
```

```
    settable(t6,28,ph6_t7m4);
    obsstepsize(15.0);
  }
  //t9m4 phase sequencing
  if (kp_scyc[1] == '9') {
    settable(t6,36,ph6_t9m4);
    obsstepsize(15.0);
  }
}

// Phase sequencing with t5m16/t7m16
if (kp_scyc_len == 5) {
  //t5m16 phase sequencing
  if (kp_scyc[1] == '5') {
    settable(t6,80,ph6_t5m16);
    obsstepsize(30.0);
  }
  //t7m16 phase sequencing
  if (kp_scyc[1] == '7') {
    settable(t6,112,ph6_t7m16);
    obsstepsize(15.0);
  }
}
// End of the chunk to chunk phase sequencing options


// Define timings
double /*tau_B = tau_a -rof1 -gt2 -gstab -kp_pfgtc,*/
    tau_C = 0.25*npoints/sw,
    tau_G = gt1 + gstab + rof1,
    tau_H = tau_C - tau_G,
    tau_D = (droppts1/sw) + rof2,
    tau_S = tau_C + tau_D;

// Determine if odd or even transient for alternating gradients
mod2(ct,v9);


/* ACTUAL PULSE SEQUENCE ------------------------------------------------- */

// Relaxation delay (and optional sspul)
status(A);
delay(rof3);
  txphase(zero);
  xmtrphase(zero);
  obsoffset(tof);
  obspower(tpwr);
  obspwrf(tpwrf);
  delay(0.001);
  if (sspul[0]=='y') {
    if ( (lkgate_flg[0] == 'y') || (lkgate_flg[0] == 'k') )  lk_hold();
    delay(0.001);
    zgradpulse(hsglvl,hsgt);
    delay(gstab);
    rgpulse(pw,zero,rof1,rof1);
    zgradpulse(hsglvl,hsgt);
    delay(0.05);
  }
  if ((lkgate_flg[0] == 'y') || (lkgate_flg[0] == 'k'))  lk_sample();
  delay(d1);
  if ( (lkgate_flg[0] == 'y') || (lkgate_flg[0] == 'k') )  lk_hold();
  delay(0.001);

// Pulse sequence proper
status(B);

// 90 PULSE
```

```
rgpulse(pw,v1,rof1,0.0);

// "FIFTH" REPETITION
if (numreps > 4) {

  // HARD 180

    // Set pulse power for hard 180
    obspower(pplvl);
    if (tpwrf!=pplvlf) obspwrf(pplvlf);

    // CTP grad
    // Opposite gradient polarity for consecutive repetitions
    delay(tau_H);

    ifzero(v9); zgradpulse(gzlvl1,gt1);
    elsenz(v9);  zgradpulse(-gzlvl1,gt1);
    endif(v9);

    delay(gstab);

    // Hard 180
    if (BIPflg[0] == 'y') {
      shaped_pulse(shp_BIP,pw_BIP,v2,rof1,rof1);
    } else {
      rgpulse(pp,v2,rof1,rof1);
    }

    // Set selective pulse power
    obspower(pwr180_a);
    if (tpwrf!=pplvlf) obspwrf(4095.0);

    // CTP grad
    ifzero(v9); zgradpulse(gzlvl1,gt1);
    elsenz(v9);  zgradpulse(-gzlvl1,gt1);
    endif(v9);

    delay(gstab + tau_H);


  // SELECTIVE 180

    // CTP grad
    // shift the delay from around the hard pulse in the normal sequence
    // to around the selective pulse in the repeating block
    delay(tau_S + gstab + tau_E);


    // CTP grad
    ifzero(v9); zgradpulse(gzlvl2,gt2);
    elsenz(v9); zgradpulse(-gzlvl2,gt2);
    endif(v9);

    delay(gstab);


    // Turn on grad for ZS experiment
    if (gzlvl7>0.0) {
      rgradient('z',gzlvl7);
      delay(kp_pfgtc);
    }

    // Selective 180
    shaped_pulse(shp_a,pw180_a,v25,rof1,rof1);

    // Turn off grad for ZS experiment
    if (gzlvl7>0.0) {
```

```
      rgradient('z',0.0);
      delay(kp_pfgtc);
    }

    // CTP grad
    delay(gstab);

    ifzero(v9); zgradpulse(gzlvl2,gt2);
    elsenz(v9); zgradpulse(-gzlvl2,gt2);
    endif(v9);

    delay(gstab + tau_S + tau_E);

}

// "FOURTH" REPETITION
if (numreps > 3) {

  // HARD 180

    // Set pulse power for hard 180
    obspower(pplvl);
    if (tpwrf!=pplvlf) obspwrf(pplvlf);

    // CTP grad
    // Opposite gradient polarity for consecutive repetitions
    delay(tau_H);

    ifzero(v9); zgradpulse(-gzlvl1,gt1);
    elsenz(v9);  zgradpulse(gzlvl1,gt1);
    endif(v9);

    delay(gstab);

    // Hard 180
    if (BIPflg[0] == 'y') {
      shaped_pulse(shp_BIP,pw_BIP,v2,rof1,rof1);
    } else {
      rgpulse(pp,v2,rof1,rof1);
    }

    // Set selective pulse power
    obspower(pwr180_a);
    if (tpwrf!=pplvlf) obspwrf(4095.0);

    // CTP grad
    ifzero(v9); zgradpulse(-gzlvl1,gt1);
    elsenz(v9);  zgradpulse(gzlvl1,gt1);
    endif(v9);

    delay(gstab + tau_H);


  // SELECTIVE 180

    // CTP grad
    // shift the delay from around the hard pulse in the normal sequence
    // to around the selective pulse in the repeating block
    delay(tau_S + gstab + tau_E);


    // CTP grad
    ifzero(v9); zgradpulse(-gzlvl2,gt2);
    elsenz(v9); zgradpulse(gzlvl2,gt2);
    endif(v9);

    delay(gstab);
```

```
    // Turn on grad for ZS experiment
    if (gzlvl7>0.0) {
      rgradient('z',gzlvl7);
      delay(kp_pfgtc);
    }

    // Selective 180
    shaped_pulse(shp_a,pw180_a,v24,rof1,rof1);

    // Turn off grad for ZS experiment
    if (gzlvl7>0.0) {
      rgradient('z',0.0);
      delay(kp_pfgtc);
    }

    // CTP grad
    delay(gstab);

    ifzero(v9); zgradpulse(-gzlvl2,gt2);
    elsenz(v9); zgradpulse(gzlvl2,gt2);
    endif(v9);

    delay(gstab + tau_S + tau_E);

}


// "THIRD" REPETITION
if (numreps > 2) {

  // HARD 180

    // Set pulse power for hard 180
    obspower(pplvl);
    if (tpwrf!=pplvlf) obspwrf(pplvlf);

    // CTP grad
    // Opposite gradient polarity for consecutive repetitions
    delay(tau_H);

    ifzero(v9); zgradpulse(gzlvl1,gt1);
    elsenz(v9);  zgradpulse(-gzlvl1,gt1);
    endif(v9);

    delay(gstab);

    // Hard 180
    if (BIPflg[0] == 'y') {
      shaped_pulse(shp_BIP,pw_BIP,v2,rof1,rof1);
    } else {
      rgpulse(pp,v2,rof1,rof1);
    }

    // Set selective pulse power
    obspower(pwr180_a);
    if (tpwrf!=pplvlf) obspwrf(4095.0);

    // CTP grad
    ifzero(v9); zgradpulse(gzlvl1,gt1);
    elsenz(v9);  zgradpulse(-gzlvl1,gt1);
    endif(v9);

    delay(gstab + tau_H);
```

```
  // SELECTIVE 180

    // CTP grad
    // shift the delay from around the hard pulse in the normal sequence
    // to around the selective pulse in the repeating block
    delay(tau_S + gstab + tau_E);


    // CTP grad
    ifzero(v9); zgradpulse(gzlvl2,gt2);
    elsenz(v9); zgradpulse(-gzlvl2,gt2);
    endif(v9);

    delay(gstab);


    // Turn on grad for ZS experiment
    if (gzlvl7>0.0) {
      rgradient('z',gzlvl7);
      delay(kp_pfgtc);
    }

    // Selective 180
    shaped_pulse(shp_a,pw180_a,v23,rof1,rof1);


    // Turn off grad for ZS experiment
    if (gzlvl7>0.0) {
      rgradient('z',0.0);
      delay(kp_pfgtc);
    }

    // CTP grad
    delay(gstab);

    ifzero(v9); zgradpulse(gzlvl2,gt2);
    elsenz(v9); zgradpulse(-gzlvl2,gt2);
    endif(v9);

    delay(gstab + tau_S + tau_E);

}


// "SECOND" REPETITION
if (numreps > 1) {

  // HARD 180

    // Set pulse power for hard 180
    obspower(pplvl);
    if (tpwrf!=pplvlf) obspwrf(pplvlf);

    // CTP grad
    delay(tau_H);
    ifzero(v9); zgradpulse(-gzlvl1,gt1);
    elsenz(v9); zgradpulse(gzlvl1,gt1);
    endif(v9);
    delay(gstab);

    // Hard 180
    if (BIPflg[0] == 'y') {
      shaped_pulse(shp_BIP,pw_BIP,v2,rof1,rof1);
    } else {
      rgpulse(pp,v2,rof1,rof1);
    }
```

```
    // Set selective pulse power
    obspower(pwr180_a);
    if (tpwrf!=pplvlf) obspwrf(4095.0);

    // CTP grad
    ifzero(v9); zgradpulse(-gzlvl1,gt1);
    elsenz(v9); zgradpulse(gzlvl1,gt1);
    endif(v9);
    delay(gstab + tau_H);


  // SELECTIVE 180

    // Extra delay
    if (numreps % 2 == 0) {
      delay(tau_D + tau_E);
    } else {
      delay(tau_S + tau_E);
    }

    // CTP grad
    delay(gstab);
    ifzero(v9); zgradpulse(-gzlvl2,gt2);
    elsenz(v9); zgradpulse(gzlvl2,gt2);
    endif(v9);
    delay(gstab);

    // Turn on grad for ZS experiment
    if (gzlvl7>0.0) {
      rgradient('z',gzlvl7);
      delay(kp_pfgtc);
    }

    // Selective 180
    shaped_pulse(shp_a,pw180_a,v22,rof1,rof1);

    // Turn off grad for ZS experiment
    if (gzlvl7>0.0) {
      rgradient('z',0.0);
      delay(kp_pfgtc);
    }

    // CTP grad
    delay(gstab);
    ifzero(v9); zgradpulse(-gzlvl2,gt2);
    elsenz(v9); zgradpulse(gzlvl2,gt2);
    endif(v9);
    delay(gstab);

    // Extra delay
    if (numreps % 2 == 0) {
      delay(tau_D + tau_E);
    } else {
      delay(tau_S + tau_E);
    }
}

// "FIRST" REPETITION

// 1st t1/2 evolution
delay(d2/2.0);

// Set pulse power for hard 180
obspower(pplvl);
if (tpwrf!=pplvlf) obspwrf(pplvlf);

// CTP grad
```

```
if (numreps % 2 == 0) {
  delay(tau_C);
}
delay(tau_H);
ifzero(v9); zgradpulse(gzlvl1,gt1);
elsenz(v9); zgradpulse(-gzlvl1,gt1);
endif(v9);
delay(gstab);

// Hard 180
if (BIPflg[0] == 'y') {
  shaped_pulse(shp_BIP,pw_BIP,v2,rof1,rof1);
} else {
  rgpulse(pp,v2,rof1,rof1);
}

// Set selective pulse power
obspower(pwr180_a);
if (tpwrf!=pplvlf) obspwrf(4095.0);

// CTP grad
ifzero(v9); zgradpulse(gzlvl1,gt1);
elsenz(v9); zgradpulse(-gzlvl1,gt1);
endif(v9);

delay(gstab + tau_H);
if (numreps % 2 == 0) {
  delay(tau_C);
}

// Drop points
delay(tau_D);

// CTP grad
delay(gstab + tau_C + tau_E);
ifzero(v9); zgradpulse(gzlvl2,gt2);
elsenz(v9); zgradpulse(-gzlvl2,gt2);
endif(v9);
delay(gstab);

// Turn on grad for ZS experiment
if (gzlvl7>0.0) {
  rgradient('z',gzlvl7);
  delay(kp_pfgtc);
}

// Selective 180
shaped_pulse(shp_a,pw180_a,v21,rof1,rof1);


// Turn off grad for ZS experiment
if (gzlvl7>0.0) {
  rgradient('z',0.0);
  delay(kp_pfgtc);
}

// CTP grad
// if/else to have the longer delay after the gradient pulse
delay(gstab);
ifzero(v9); zgradpulse(gzlvl2,gt2);
elsenz(v9); zgradpulse(-gzlvl2,gt2);
endif(v9);
delay(gstab + tau_C + tau_E);

// 2nd t1/2 evolution
delay(d2/2.0);
```

```
// ACQUISITION
status(C);
startacq(getval("alfa"));


loop(v10,v11);
//acquire a chunk of data
  sample((droppts1+npoints+droppts2)/sw);
  recoff();

obspower(pwr180_p);
obsunblank();

//setup phase sequencing
getelem(t6,v11,v7);
if (kp_scyc[0]=='t') {
  xmtrphase(v7);
} else {
  add(v7,v4,v14);
}
//apply the next PFG level
mod4(v11,v12);
delay(tau_r);
ifzero(v12);
 zgradpulse(1.0*ACQ_gzlvl1,ACQ_gt1);
 delay(ACQ_gstab);
endif(v12);
ifrtEQ(v12,one,v13);
 zgradpulse(1.0*ACQ_gzlvl2,ACQ_gt1);
 delay(ACQ_gstab);
endif(v13);
ifrtEQ(v12,two,v13);
 zgradpulse(1.0*ACQ_gzlvl3,ACQ_gt1);
 delay(ACQ_gstab);
endif(v13);
ifrtEQ(v12,three,v13);
 zgradpulse(1.0*ACQ_gzlvl4,ACQ_gt1);
 delay(ACQ_gstab);
endif(v13);

shaped_pulse(shp_p,pw180_p,v14,rof1,rof1);

if (ACQ_gstab > tau_r) {
  delay(tau_r-rof3);
  ifzero(v12);
   zgradpulse(-1.0*ACQ_gzlvl1,ACQ_gt1);
   delay(ACQ_gstab);
  endif(v12);
  ifrtEQ(v12,one,v13);
   zgradpulse(-1.0*ACQ_gzlvl2,ACQ_gt1);
   delay(ACQ_gstab);
  endif(v13);
  ifrtEQ(v12,two,v13);
   zgradpulse(-1.0*ACQ_gzlvl3,ACQ_gt1);
   delay(ACQ_gstab);
  endif(v13);
  ifrtEQ(v12,three,v13);
   zgradpulse(-1.0*ACQ_gzlvl4,ACQ_gt1);
   delay(ACQ_gstab);
  endif(v13);
} else {
  ifzero(v12);
   delay(ACQ_gstab);
   zgradpulse(-1.0*ACQ_gzlvl1,ACQ_gt1);
  endif(v12);
  ifrtEQ(v12,one,v13);
```

```
   delay(ACQ_gstab);
   zgradpulse(-1.0*ACQ_gzlvl2,ACQ_gt1);
  endif(v13);
  ifrtEQ(v12,two,v13);
   delay(ACQ_gstab);
   zgradpulse(-1.0*ACQ_gzlvl3,ACQ_gt1);
  endif(v13);
  ifrtEQ(v12,three,v13);
   delay(ACQ_gstab);
   zgradpulse(-1.0*ACQ_gzlvl4,ACQ_gt1);
  endif(v13);
  delay(tau_r-rof3);
}

  rcvron();
endloop(v11);

  sample((droppts1+npoints+droppts2)/sw);
  recoff();
  endacq();

delay(0.05);
  if ( (lkgate_flg[0] == 'y') || (lkgate_flg[0] == 'k') )  lk_sample();
delay(0.05);

}
```

S11.3. Bruker interferogram acquisition mode pulse sequence code

```
; UoM_EXQUISITE_if_2d
; Dervived from hf_EXQUISITE_if v 0.2
; Howard Foster
; University of Manchester
; Version 1.0
; Last modified: 01/05/2024
;
; EXQUISITE pure shift NMR interferogram acquisition mode experiment with iterations of the
; J-refocusing element to provide an exponential attenuation of signal integral.
;
; Set up as a series of 2D experiments, each with a different value of cnst23, which controls
; the number of iterations.
;
; Data can be reconstructed using a macro available at http://nmr.chemistry.manchester.ac.uk
;
; References:
; Band-selection: (1) L. Castanar, P. Nolis, A. Virgili, T. Parella, Chem. Eur. J., 19, 17283-
17286, (2013)
;                 (2) J. Ying, J. Roche, A. Bax, J. Magn. Reson., 241, 97-102, (2014)
;                 (3) R. W. Adams, L. Byrne, P. kiraly, M. Foroozabdeh, L. Paudel, M. Nilsson,
J. Clayden, G. A. Morris, Chem. Commun., 50, 2512, (2014)
; Interferogram:  (1) K. Zangger, H. Sterk, J. Magn. Reson., 124, 486-489, (1997)
;                   (2) J. A. Aguilar, S. Faulkner, M. Nilsson, G. A. Morris, 49, 3901-3903,
(2010)
;
;
; WARNING: In recent versions of TopSpin (4.3+) on Windows systems, users may experience an
error with the message
; "The system detected an overrun of a stack-based buffer in this application" when attempting
to run the wvm command.
; It seems that this error is related to the length of the phase cycles of the selective 180°
RF pulses (ph11 - ph15).
; Shortening the length of these phase cycles to 64 steps appears to solve the issue, but does
preclude the use of
; experiments with five iterations.
;
; $CLASS=HighRes; $DIM=2D
; $TYPE=
; $SUBTYPE=
; $COMMENT=

#include <Avance.incl>
#include <Delay.incl>
#include <Grad.incl>

;WaveMaker definitions
;cnst50: band-width of the band-selective pulse [Hz]

;sp12(p12):wvm:hf_shape_a_wvm:f1 userA1(cnst50 Hz) ss=10.0us;

define delay tauC
define delay tauG
define delay tauH
define delay tauD
define delay tauS

"d0 = 0"
"in0 = inf1/2"

"p2 = p1*2"

"p16 = 1m"
"d17 = 1m"
```

```
"p17 = 1m"

"tauC = in0/2"
"tauG = p16 + d16 + 50u"
"tauH = tauC - tauG"
"tauD = dw*2*cnst4"
"tauS = tauC + tauD"

"l0 = cnst23"

"d11 = 30m+1s/(1+cnst50)-1s/(1+cnst50)"
"d11 = 30m"

"acqt0 = 0"
baseopt_echo


1 ze
2 d11
  10u BLKGRAMP
  10u LOCKH_OFF
  d1 pl1:f1
  10u LOCKH_ON
  10u UNBLKGRAMP

3 p1 ph1

; "FIFTH" ITERATION
if "l0 > 4" {

; hard 180
  50u
  tauH
  if "nsdone % 2 == 0" {
    p16:gp1
  } else {
    p16:gp1*-1.0
  }
  d16
  p2 ph2
  if "nsdone % 2 == 0" {
    p16:gp1
  } else {
    p16:gp1*-1.0
  }
  d16
  tauH
  50u

; sel. 180
  50u
  d20
  tauS
  d17
  if "nsdone % 2 == 0" {
    p17:gp2
  } else {
    p17:gp2*-1.0
  }
  d17
  (p12:sp12 ph15):f1
  d17
  if "nsdone % 2 == 0" {
    p17:gp2
  } else {
    p17:gp2*-1.0
  }
```

```
  d17
  tauS
  d20
  50u
}

; "FOURTH" ITERATION
if "l0 > 3" {

; hard 180
  50u pl1:f1
  tauH
  if "nsdone % 2 == 0" {
    p16:gp1*-1.0
  } else {
    p16:gp1
  }
  d16
  p2 ph2
  if "nsdone % 2 == 0" {
    p16:gp1*-1.0
  } else {
    p16:gp1
  }
  d16
  tauH
  50u

; sel. 180
  50u
  d20
  tauS
  d17
  if "nsdone % 2 == 0" {
    p17:gp2*-1.0
  } else {
    p17:gp2
  }
  d17
  (p12:sp12 ph14):f1
  d17
  if "nsdone % 2 == 0" {
    p17:gp2*-1.0
  } else {
    p17:gp2
  }
  d17
  tauS
  d20
  50u
}

; "THIRD" ITERATION
if "l0 > 2" {

; hard 180
  50u pl1:f1
  tauH
  if "nsdone % 2 == 0" {
    p16:gp1
  } else {
    p16:gp1*-1.0
  }
  d16
  p2 ph2
  if "nsdone % 2 == 0" {
    p16:gp1
```

```
  } else {
    p16:gp1*-1.0
  }
  d16
  tauH
  50u

; sel. 180
  50u
  d20
  tauS
  d17
  if "nsdone % 2 == 0" {
    p17:gp2
  } else {
    p17:gp2*-1.0
  }
  d17
  (p12:sp12 ph13):f1
  d17
  if "nsdone % 2 == 0" {
    p17:gp2
  } else {
    p17:gp2*-1.0
  }
  d17
  tauS
  d20
  50u
}

; "SECOND" ITERATION
if "l0 > 1" {

; hard 180
  50u pl1:f1
  tauH
  if "nsdone % 2 == 0" {
    p16:gp1*-1.0
  } else {
    p16:gp1
  }
  d16
  p2 ph2
  if "nsdone % 2 == 0" {
    p16:gp1*-1.0
  } else {
    p16:gp1
  }
  d16
  tauH
  50u

; sel. 180
  50u
  d20
  if "l0 % 2 == 0" {
    tauD
  } else {
    tauS
  }
  d17
  if "nsdone % 2 == 0" {
    p17:gp2*-1.0
  } else {
    p17:gp2
  }
```

```
  d17
  (p12:sp12 ph12):f1
  d17
  if "nsdone % 2 == 0" {
    p17:gp2*-1.0
  } else {
    p17:gp2
  }
  d17
  if "l0 % 2 == 0" {
    tauD
  } else {
    tauS
  }
  d20
  50u

}

; "FIRST" ITERATION

; first half of t1 evolution
  d0

; hard 180
  50u pl1:f1
  if "l0 % 2 == 0" {
    tauC
  }
  tauH
  if "nsdone % 2 == 0" {
    p16:gp1
  } else {
    p16:gp1*-1.0
  }
  d16
  p2 ph2
  if "nsdone % 2 == 0" {
    p16:gp1
  } else {
    p16:gp1*-1.0
  }
  d16
  tauH
  if "l0 % 2 == 0" {
    tauC
  }
  50u

; drop points
  tauD

; sel. 180
  50u
  d20
  tauC
  d17
  if "nsdone % 2 == 0" {
    p17:gp2
  } else {
    p17:gp2*-1.0
  }
  d17
  (p12:sp12 ph11):f1
  d17
  if "nsdone % 2 == 0" {
    p17:gp2
```

```
  } else {
    p17:gp2*-1.0
  }
  d17
  tauC
  d20
  50u BLKGRAMP

; second half of t1 evolution
  d0

; acquire
  if "l0 == 1" {
    go=2 ph21
  }
  if "l0 == 2" {
    go=2 ph22
  }
  if "l0 == 3" {
    go=2 ph23
  }
  if "l0 == 4" {
    go=2 ph24
  }
  if "l0 == 5" {
    go=2 ph25
  }
  d11 mc #0 to 2
  F1QF(caldel(d0, +in0))

  10u LOCKH_OFF

exit

; Hard pulse phase cycles
ph1  = 0 0  0 0  0 0 0 0  0 0 0 0 0 0 0 0  0 0 0 0 0 0 0 0 0 0 0 0 0 0 0 0  0 0 0 0 0 0 0 0 0
0 0 0 0 0 0 0 0 0 0 0 0 0 0 0 0 0 0 0 0 0 0 0  0 0 0 0 0 0 0 0 0 0 0 0 0 0 0 0 0 0 0 0 0 0 0 0
0 0 0 0 0 0 0 0 0 0 0 0 0 0 0 0 0 0 0 0 0 0 0 0 0 0 0 0 0 0 0 0 0 0 0 0 0 0 0 0; initial 90
ph2  = 0 0  0 0  0 0 0 0  0 0 0 0 0 0 0 0  0 0 0 0 0 0 0 0 0 0 0 0 0 0 0 0  0 0 0 0 0 0 0 0 0
0 0 0 0 0 0 0 0 0 0 0 0 0 0 0 0 0 0 0 0 0 0 0  0 0 0 0 0 0 0 0 0 0 0 0 0 0 0 0 0 0 0 0 0 0 0 0
0 0 0 0 0 0 0 0 0 0 0 0 0 0 0 0 0 0 0 0 0 0 0 0 0 0 0 0 0 0 0 0 0 0 0 0 0 0 0 0;  hard 180

; Selective pulse phase cycles
ph11 = 0 1  0 1  0 1 0 1  0 1 0 1 0 1 0 1  0 1 0 1 0 1 0 1 0 1 0 1 0 1 0 1  0 1 0 1 0 1 0 1 0
1 0 1 0 1 0 1 0 1 0 1 0 1 0 1 0 1 0 1 0 1 0 1  0 1 0 1 0 1 0 1 0 1 0 1 0 1 0 1 0 1 0 1 0 1 0 1
0 1 0 1 0 1 0 1 0 1 0 1 0 1 0 1 0 1 0 1 0 1 0 1 0 1 0 1 0 1 0 1 0 1 0 1 0 1 0 1;  1st
iteration sel. 180
ph12 = 0 0  1 1  2 2 3 3  0 0 1 1 2 2 3 3  0 0 1 1 2 2 3 3 0 0 1 1 2 2 3 3  0 0 1 1 2 2 3 3 0
0 1 1 2 2 3 3 0 0 1 1 2 2 3 3 0 0 1 1 2 2 3 3  0 0 1 1 2 2 3 3 0 0 1 1 2 2 3 3 0 0 1 1 2 2 3 3
0 0 1 1 2 2 3 3 0 0 1 1 2 2 3 3 0 0 1 1 2 2 3 3 0 0 1 1 2 2 3 3 0 0 1 1 2 2 3 3;  2nd
iteration sel. 180
ph13 = 0 0  0 0  0 0 0 0  1 1 1 1 1 1 1 1  0 0 0 0 0 0 0 0 1 1 1 1 1 1 1 1  0 0 0 0 0 0 0 0 1
1 1 1 1 1 1 1 0 0 0 0 0 0 0 0 1 1 1 1 1 1 1 1  0 0 0 0 0 0 0 0 1 1 1 1 1 1 1 1 0 0 0 0 0 0 0 0
1 1 1 1 1 1 1 1 0 0 0 0 0 0 0 0 1 1 1 1 1 1 1 1 0 0 0 0 0 0 0 0 1 1 1 1 1 1 1 1;  3rd
iteration sel. 180
ph14 = 0 0  0 0  0 0 0 0  0 0 0 0 0 0 0 0  1 1 1 1 1 1 1 1 1 1 1 1 1 1 1 1  2 2 2 2 2 2 2 2 2
2 2 2 2 2 2 2 3 3 3 3 3 3 3 3 3 3 3 3 3 3 3 3  0 0 0 0 0 0 0 0 0 0 0 0 0 0 0 0 1 1 1 1 1 1 1 1
1 1 1 1 1 1 1 1 2 2 2 2 2 2 2 2 2 2 2 2 2 2 2 2 3 3 3 3 3 3 3 3 3 3 3 3 3 3 3 3; 4th iteration
sel. 180
ph15 = 0 0  0 0  0 0 0 0  0 0 0 0 0 0 0 0  0 0 0 0 0 0 0 0 0 0 0 0 0 0 0 0  0 0 0 0 0 0 0 0 0
0 0 0 0 0 0 0 0 0 0 0 0 0 0 0 0 0 0 0 0 0 0 0  1 1 1 1 1 1 1 1 1 1 1 1 1 1 1 1 1 1 1 1 1 1 1 1
1 1 1 1 1 1 1 1 1 1 1 1 1 1 1 1 1 1 1 1 1 1 1 1 1 1 1 1 1 1 1 1 1 1 1 1 1 1 1 1; 5th iteration
sel. 180

; Receiver phase cycles
ph21 = 0 2  0 2  0 2 0 2  0 2 0 2 0 2 0 2  0 2 0 2 0 2 0 2 0 2 0 2 0 2 0 2  0 2 0 2 0 2 0 2 0
2 0 2 0 2 0 2 0 2 0 2 0 2 0 2 0 2 0 2 0 2 0 2  0 2 0 2 0 2 0 2 0 2 0 2 0 2 0 2 0 2 0 2 0 2 0 2
```

```
0 2 0 2 0 2 0 2 0 2 0 2 0 2 0 2 0 2 0 2 0 2 0 2 0 2 0 2 0 2 0 2 0 2 0 2 0 2 0 2 0 2 0 2; 1st
iteration rec. phase
ph22 = 0 2  2 0  0 2 2 0  0 2 2 0 0 2 2 0  0 2 2 0 0 2 2 0 0 2 2 0 0 2 2 0  0 2 2 0 0 2 2 0 0
2 2 0 0 2 2 0 0 2 2 0 0 2 2 0 0 2 2 0 0 2 2 0  0 2 2 0 0 2 2 0 0 2 2 0 0 2 2 0 0 2 2 0 0 2 2 0
0 2 2 0 0 2 2 0 0 2 2 0 0 2 2 0 0 2 2 0 0 2 2 0 0 2 2 0 0 2 2 0 0 2 2 0 0 2 2 0; 2nd
iteration rec. phase
ph23 = 0 2  2 0  0 2 2 0  2 0 0 2 2 0 0 2  0 2 2 0 0 2 2 0 2 0 0 2 2 0 0 2  0 2 2 0 0 2 2 0 2
0 0 2 2 0 0 2 0 2 2 0 0 2 2 0 2 0 0 2 2 0 0 2  0 2 2 0 0 2 2 0 2 0 0 2 2 0 0 2 0 2 2 0 0 2 2 0
2 0 0 2 2 0 0 2 0 2 2 0 0 2 2 0 2 0 0 2 2 0 0 2 0 2 2 0 0 2 2 0 2 0 0 2 2 0 0 2; 3rd
iteration rec. phase
ph24 = 0 2  2 0  0 2 2 0  2 0 0 2 2 0 0 2  2 0 0 2 2 0 0 2 0 2 2 0 0 2 2 0  0 2 2 0 0 2 2 0 2
0 0 2 2 0 0 2 2 0 0 2 2 0 0 2 0 2 2 0 0 2 2 0  0 2 2 0 0 2 2 0 2 0 0 2 2 0 0 2 2 0 0 2 2 0 0 2
0 2 2 0 0 2 2 0 0 2 2 0 0 2 2 0 2 0 0 2 2 0 0 2 2 0 0 2 2 0 0 2 0 2 2 0 0 2 2 0; 4th
iteration rec. phase
ph25 = 0 2  2 0  0 2 2 0  2 0 0 2 2 0 0 2  2 0 0 2 2 0 0 2 0 2 2 0 0 2 2 0  0 2 2 0 0 2 2 0 2
0 0 2 2 0 0 2 2 0 0 2 2 0 0 2 0 2 2 0 0 2 2 0  2 0 0 2 2 0 0 2 0 2 2 0 0 2 2 0 0 2 2 0 0 2 2 0
2 0 0 2 2 0 0 2 2 0 0 2 2 0 0 2 0 2 2 0 0 2 2 0 0 2 2 0 0 2 2 0 2 0 0 2 2 0 0 2; 5th iteration
rec. phase
ph31 = 0

;POWER LEVEL
;pl0 : zero power [0 W]
;pl1 : power level for pulse (default)

;PULSES
;p1  : hard 90 pulse (high power)
;p16 : duration of CTP gradients of hard 180 pulse [1 ms]
;p17 : duration of CTP gradients of selective 180 pulse [1 ms]
;p12 : duration of 180 selective pulse (low power)

;DELAYS
;d0  : incremented delay
;d1  : relaxation delay
;d16 : recovery delay for CTP gradients of hard 180 pulse       [0.95 ms]
;d17 : recovery delay for CTP gradients of selective 180 pulse  [1 ms]

;SHAPE PULSE
;sp12 : selective 180 shaped pulse

;GRADIENTS
;gpz1: 31% [CTP gradient for hard 180 pulse]
;gpz2: 43% [CTP gradient for selective 180 pulse]
;gpnam1: SMSQ10.100
;gpnam2: SMSQ10.100

;OTHERS
;cnst23: number of iterations     [2-5]
;cnst4 : number of drop points    [4 or 8]
;td1   : number of chunks     [16-128]
;in0   : 1 / (2*SW2)
;nd0   : 2
;MC2   : QF
;d1    : relaxation delay; 1-7 * T1
;ns    : 8,16,64,128 for cnst23 = 2,3,4,5
;ds    : 2
```

S12. Varian/Agilent setup and processing macros

S12.1. Setup macros

S12.1.1. UoM_setup_EXQUISITE

```
/* UoM_setup_EXQUISITE ----------------------------------------------------

 Setup macro for parameters common to all EXQUISITE pure shift NMR
 experiments.

 Argument $1:
  $1 = 0: Only create parameters which don't exist
  $1 = 1: Create parameters which don't exist and set all parameters to
          default values

 Howard M. Foster
 The University of Manchester
 Manchester NMR Methodology Group
 v1.0 31/01/2024
-------------------------------------------------------------------------*/

$kp_setpar=0
if ($#>0) then $kp_setpar=$1 else $kp_setpar=1 endif

exists('numreps','parameter'):$e
if $e=0 then create('numreps','integer') setlimit('numreps',5,1,1) array('numreps',5,1,1) endif
if ($kp_setpar<>0) then array('numreps',5,1,1) endif
exists('simple_phcycle','parameter'):$e
if   $e=0   then   create('simple_phcycle','string')   setenumeral('simple_phcycle',2,'n','y')
simple_phcycle='n' endif if ($kp_setpar<>0) then simple_phcycle='n' endif
exists('tau_E','parameter'):$e
if $e=0 then create('tau_E','delay') setlimit('tau_E',8192,0,0.0000000125) tau_E=0.0 endif if
($kp_setpar<>0) then tau_E=0.0 endif

// Reset 'array' parameter to only be for numreps
array = 'numreps'
```

S12.1.2. UoM_setup_EXQUISITE_if

```
/* UoM_setup_EXQUISITE_if --------------------------------------------------

 Setup macro for an EXQUISITE pure shift NMR experiment in semi-real-time
 acquisition mode.

 Argument $1:
  $1 = 0: Only create parameters which don't exist
  $1 = 1: Create parameters which don't exist and set all parameters to
          default values

 Derived from the "UoM_setup_1d_if_BS" macro

 Howard M. Foster
 The University of Manchester
 Manchester NMR Methodology Group
 v1.0 31/01/2024
--------------------------------------------------------------------------*/

$kp_setpar=0
if ($#>0) then $kp_setpar=$1 else $kp_setpar=1 endif
seqgen('UoM_EXQUISITE_if.c')
if ($kp_setpar<>0) then seqfil='UoM_EXQUISITE_if' endif

// Basic parameters
exists('pw','parameter'):$e
if $e=0 then create('pw','pulse') setlimit('pw',100,0,0.0125) pw=10 endif
exists('tpwr','parameter'):$e
if $e=0 then create('tpwr','real') setlimit('tpwr',63,-16,1) tpwr=58 endif
exists('tpwrf','parameter'):$e
if $e=0 then create('tpwrf','real') setlimit('tpwrf',4095,0,1) tpwrf=4095 endif if
($kp_setpar<>0) then tpwrf=4095 endif
exists('pw90','parameter'):$e
if $e=0 then create('pw90','pulse') setlimit('pw90',100,0,0.0125) pw90=pw endif if
($kp_setpar<>0) then pw90=pw endif
exists('ref_pw90','parameter'):$e
if $e=0 then create('ref_pw90','pulse') setlimit('ref_pw90',1000,0,0.0125) ref_pw90=pw90 endif
if ($kp_setpar<>0) then ref_pw90=pw90 endif
exists('ref_pwr','parameter'):$e
if $e=0 then create('ref_pwr','real') setlimit('ref_pwr',63,-16,1) ref_pwr=tpwr endif if
($kp_setpar<>0) then ref_pwr=tpwr endif

// Hard 180 parameters
exists('pp','parameter'):$e
if $e=0 then create('pp','pulse') setlimit('pp',200,0,0.0125) pp=2.0*pw endif if ($kp_setpar<>0)
then pp=2.0*pw endif
exists('pplvl','parameter'):$e
if $e=0 then create('pplvl','real') setlimit('pplvl',63,-16,1) pplvl=tpwr endif if
($kp_setpar<>0) then pplvl=tpwr endif
exists('pplvlf','parameter'):$e
if $e=0 then create('pplvlf','real') setlimit('pplvlf',4095,0,1) pplvlf=tpwrf endif if
($kp_setpar<>0) then pplvlf=tpwrf endif

// Selective 180 for active spins
exists('pw180_a','parameter'):$e
if $e=0 then create('pw180_a','pulse') setlimit('pw180_a',500000,0,0.0125) pw180_a=18500 endif
if ($kp_setpar<>0) then pw180_a=18500 endif
exists('pwr180_a','parameter'):$e
if $e=0 then create('pwr180_a','real') setlimit('pwr180_a',63,-16,1) pwr180_a=-16 endif if
($kp_setpar<>0) then pwr180_a=-16 endif
exists('shp_a','parameter'):$e
if $e=0 then create('shp_a','string') shp_a='kp_ifBASHD_active' endif if ($kp_setpar<>0) then
shp_a='kp_ifBASHD_active' endif
exists('bw_a','parameter'):$e
```

```
if $e=0 then create('bw_a','real') setlimit('bw_a',100000,-100000,1) bw_a=100
setgroup('bw_a','processing') endif if ($kp_setpar<>0) then bw_a=100 endif
exists('kp_beta_a','parameter'):$e
if $e=0 then create('kp_beta_a','real') setlimit('kp_beta_a',360,0,0.01) kp_beta_a=180 endif if
($kp_setpar<>0) then kp_beta_a=180 endif
exists('kp_phincr_a','parameter'):$e
if $e=0 then create('kp_phincr_a','real') setlimit('kp_phincr_a',180,-180,0.001) kp_phincr_a=0
endif if ($kp_setpar<>0) then kp_phincr_a=0 endif
exists('kp_stepsize_a','parameter'):$e
if $e=0 then create('kp_stepsize_a','real') setlimit('kp_stepsize_a',100,0.25,0.25)
kp_stepsize_a=5 endif if ($kp_setpar<>0) then kp_stepsize_a=5 endif
exists('kp_wave_a','parameter'):$e
if $e=0 then create('kp_wave_a','string') kp_wave_a='rsnob' endif if ($kp_setpar<>0) then
kp_wave_a='rsnob' endif
exists('offset','parameter'):$e
if $e=0 then create('offset','frequency') setlimit('offset',100000,-100000,0.1) offset=0
setgroup('offset','processing') endif if ($kp_setpar<>0) then offset=0 endif
exists('tau_a','parameter'):$e
if $e=0 then create('tau_a','delay') setlimit('tau_a',8192,0,0.0000000125) tau_a=0.003 endif if
($kp_setpar<>0) then tau_a=0.003 endif

// Basic DDR parameters
exists('rof3','parameter'):$e
if $e=0 then create('rof3','pulse') setlimit('rof3',100,0,0.0125) rof3=10.0 endif if
($kp_setpar<>0) then rof3=10.0 endif
exists('ddrcr','parameter'):$e
if $e=0 then create('ddrcr','integer') setlimit('ddrcr',1000,2,1) ddrcr=70 endif if
($kp_setpar<>0) then ddrcr=70 endif
exists('rof1','parameter'):$e
if $e=0 then create('rof1','pulse') setlimit('rof1',100,0,0.0125) rof1=4.0 endif if
($kp_setpar<>0) then rof1=4.0 endif
exists('rof2','parameter'):$e
if $e=0 then create('rof2','pulse') setlimit('rof2',100,0,0.0125) rof2=10.0 endif if
($kp_setpar<>0) then rof2=10.0 endif
exists('alfa','parameter'):$e
if $e=0 then create('alfa','pulse') setlimit('alfa',1000,0,0.4) alfa=10.0 endif if
($kp_setpar<>0) then alfa=10.0 endif
exists('ddrtc','parameter'):$e
if $e=0 then create('ddrtc','pulse') setlimit('ddrtc',1000,0,0.4) ddrtc=20.0 endif if
($kp_setpar<>0) then ddrtc=20.0 endif
exists('ddrpm','parameter'):$e
if $e=0 then create('ddrpm','string') ddrpm='e' endif if ($kp_setpar<>0) then ddrpm='e' endif

// Setup DDR timing
if ($kp_setpar<>0) then setrc endif
setlimit('np',524288,64,2)

// String parameters
exists('sspul','parameter'):$e
if $e=0 then create('sspul','flag') setenumeral('sspul',2,'n','y') sspul='y' endif if
($kp_setpar<>0) then sspul='y' endif
exists('PFGflg','parameter'):$e
if $e=0 then create('PFGflg','flag') setenumeral('PFGflg',2,'n','y') PFGflg='y' endif if
($kp_setpar<>0) then PFGflg='y' endif
exists('altgradflg','parameter'):$e
if $e=0 then create('altgradflg','flag') setenumeral('altgradflg',2,'n','y') altgradflg='y'
endif if ($kp_setpar<>0) then altgradflg='y' endif
exists('lkgate_flg','parameter'):$e
if $e=0 then create('lkgate_flg','string') setenumeral('lkgate_flg',3,'n','y','k')
lkgate_flg='k' endif if ($kp_setpar<>0) then lkgate_flg='k' endif
exists('kp_auto','parameter'):$e
if $e=0 then create('kp_auto','flag') setenumeral('kp_auto',2,'n','y') kp_auto='y' endif if
($kp_setpar<>0) then kp_auto='y' endif

// PFG parameters
exists('hsgt','parameter'):$e
```

```
if $e=0 then create('hsgt','delay') setlimit('hsgt',0.02,0,0.0000000125) hsgt=0.002 endif if
($kp_setpar<>0) then hsgt=0.002 endif
exists('hsglvl','parameter'):$e
if $e=0 then create('hsglvl','real') setlimit('hsglvl',32768,-32767,1) hsglvl=11177 endif if
($kp_setpar<>0) then hsglvl=11177 endif
exists('gstab','parameter'):$e
if $e=0 then create('gstab','delay') setlimit('gstab',0.1,0,0.0000000125) gstab=0.001 endif if
($kp_setpar<>0) then gstab=0.001 endif
exists('gt1','parameter'):$e
if $e=0 then create('gt1','delay') setlimit('gt1',0.02,0,0.0000000125) gt1=0.001 endif if
($kp_setpar<>0) then gt1=0.001 endif
exists('gzlvl1','parameter'):$e
if $e=0 then create('gzlvl1','real') setlimit('gzlvl1',32768,-32767,1) gzlvl1=7864 endif if
($kp_setpar<>0) then gzlvl1=7864 endif
exists('gt2','parameter'):$e
if $e=0 then create('gt2','delay') setlimit('gt2',0.02,0,0.0000000125) gt2=0.001 endif if
($kp_setpar<>0) then gt2=0.001 endif
exists('gzlvl2','parameter'):$e
if $e=0 then create('gzlvl2','real') setlimit('gzlvl2',32768,-32767,1) gzlvl2=10158 endif if
($kp_setpar<>0) then gzlvl2=10158 endif
exists('gzlvl7','parameter'):$e
if $e=0 then create('gzlvl7','real') setlimit('gzlvl7',8192,-8192,1) gzlvl7=0 endif if
($kp_setpar<>0) then gzlvl7=0 endif
exists('kp_pfgtc','parameter'):$e
if $e=0 then create('kp_pfgtc','pulse') setlimit('kp_pfgtc',1000,0,0.0125) kp_pfgtc=6.0 endif
if ($kp_setpar<>0) then kp_pfgtc=6.0 endif

// Chunking parameters
exists('droppts','parameter'):$e
if $e=0 then create('droppts','real') setlimit('droppts',16384,0,1) droppts=4 endif if
($kp_setpar<>0) then droppts=4 endif

// 2D experiment parameters
exists('sw1','parameter'):$e
if $e=0 then par2D sw1=50.000 phase=0 ni=16 endif

// Phase cycling parameter
exists('kpph','parameter'):$e
if $e=0 then create('kpph','integer') setlimit('kpph',64,0,1) kpph=0 endif if ($kp_setpar<>0)
then kpph=0 endif

// BIP parameters
exists('BIPflg','parameter'):$e
if $e=0 then create('BIPflg','flag') setenumeral('BIPflg',2,'n','y') BIPflg='n' endif if
($kp_setpar<>0) then BIPflg='n' endif
exists('pw_BIP','parameter'):$e
if $e=0 then create('pw_BIP','pulse') pw_BIP=125 endif if ($kp_setpar<>0) then pw_BIP=125 endif
exists('shp_BIP','parameter'):$e
if $e=0 then create('shp_BIP','string') shp_BIP='hf_HBIP' endif if ($kp_setpar<>0) then
shp_BIP='hf_HBIP' endif
exists('pwr_BIP','parameter'):$e
if $e=0 then create('pwr_BIP','real') setlimit('pwr_BIP',63,-16,1) pwr_BIP=-16 endif if
($kp_setpar<>0) then pwr_BIP=-16 endif /*power of pulse*/

// Set up the basics for any EXQUISITE experiment
UoM_setup_EXQUISITE

if ($kp_setpar<>0) then
 nt=2 ss=2 bs=4 il='n'
 wexp = ''
 UoM_makePS9(1)

UoM_nowt fn=sw/sw1*ni*2*8 gf=ni/sw1/2 proc='ft'

endif

dps
```

S12.1.3. UoM_setup_EXQUISITE_srt

```
/* UoM_setup_EXQUISITE_srt -------------------------------------------------

 Setup macro for EXQUISITE pure shift NMR experiment in semi-real-time
 acquisition mode.

 Argument $1:
  $1 = 0: Only create parameters which don't exist
  $1 = 1: Create parameters which don't exist and set all parameters to
          default values

 Derived from the "kp_setup_srtPS_v1" macro written by Peter Kiraly.

 Howard M. Foster
 The University of Manchester
 Manchester NMR Methodology Group
 v1.0 31/01/2024
---------------------------------------------------------------------------*/


$kp_setpar=0
if ($#>0) then $kp_setpar=$1 else $kp_setpar=1 endif
seqgen('UoM_EXQUISITE_srt.c')
if ($kp_setpar<>0) then seqfil='UoM_EXQUISITE_srt' endif

// Basic parameters
exists('pw','parameter'):$e
if $e=0 then create('pw','pulse') setlimit('pw',100,0,0.0125) pw=10 endif
exists('tpwr','parameter'):$e
if $e=0 then create('tpwr','real') setlimit('tpwr',63,-16,1) tpwr=58 endif
exists('tpwrf','parameter'):$e
if $e=0 then create('tpwrf','real') setlimit('tpwrf',4095,0,1) tpwrf=4095 endif if
($kp_setpar<>0) then tpwrf=4095 endif
exists('pw90','parameter'):$e
if $e=0 then create('pw90','pulse') setlimit('pw90',100,0,0.0125) pw90=pw endif if
($kp_setpar<>0) then pw90=pw endif
exists('ref_pw90','parameter'):$e
if $e=0 then create('ref_pw90','pulse') setlimit('ref_pw90',1000,0,0.0125) ref_pw90=pw90 endif
if ($kp_setpar<>0) then ref_pw90=pw90 endif
exists('ref_pwr','parameter'):$e
if $e=0 then create('ref_pwr','real') setlimit('ref_pwr',63,-16,1) ref_pwr=tpwr endif if
($kp_setpar<>0) then ref_pwr=tpwr endif

// Hard 180 parameters
exists('pp','parameter'):$e
if $e=0 then create('pp','pulse') setlimit('pp',200,0,0.0125) pp=2.0*pw endif if ($kp_setpar<>0)
then pp=2.0*pw endif
exists('pplvl','parameter'):$e
if $e=0 then create('pplvl','real') setlimit('pplvl',63,-16,1) pplvl=tpwr endif if
($kp_setpar<>0) then pplvl=tpwr endif
exists('pplvlf','parameter'):$e
if $e=0 then create('pplvlf','real') setlimit('pplvlf',4095,0,1) pplvlf=tpwrf endif if
($kp_setpar<>0) then pplvlf=tpwrf endif

// Selective 180 for active spins
exists('pw180_a','parameter'):$e
if $e=0 then create('pw180_a','pulse') setlimit('pw180_a',500000,0,0.0125) pw180_a=18500 endif
if ($kp_setpar<>0) then pw180_a=18500 endif
exists('pwr180_a','parameter'):$e
if $e=0 then create('pwr180_a','real') setlimit('pwr180_a',63,-16,1) pwr180_a=-16 endif if
($kp_setpar<>0) then pwr180_a=-16 endif
exists('shp_a','parameter'):$e
if $e=0 then create('shp_a','string') shp_a='kp_srtACQ_active' endif if ($kp_setpar<>0) then
shp_a='kp_srtACQ_active' endif
```

```
exists('bw_a','parameter'):$e
if $e=0 then create('bw_a','real') setlimit('bw_a',10000,5,1) bw_a=100
setgroup('bw_a','processing') endif if ($kp_setpar<>0) then bw_a=100 endif
exists('kp_beta_a','parameter'):$e
if $e=0 then create('kp_beta_a','real') setlimit('kp_beta_a',360,0,0.01) kp_beta_a=180 endif if
($kp_setpar<>0) then kp_beta_a=180 endif
exists('kp_phincr_a','parameter'):$e
if $e=0 then create('kp_phincr_a','real') setlimit('kp_phincr_a',180,-180,0.001) kp_phincr_a=0
endif if ($kp_setpar<>0) then kp_phincr_a=0 endif
exists('kp_stepsize_a','parameter'):$e
if $e=0 then create('kp_stepsize_a','real') setlimit('kp_stepsize_a',100,0.25,0.25)
kp_stepsize_a=10 endif if ($kp_setpar<>0) then kp_stepsize_a=10 endif
exists('kp_wave_a','parameter'):$e
if $e=0 then create('kp_wave_a','string') kp_wave_a='rsnob' endif if ($kp_setpar<>0) then
kp_wave_a='rsnob' endif
exists('tau_a','parameter'):$e
if $e=0 then create('tau_a','delay') setlimit('tau_a',8192,0,0.0000000125) tau_a=0.005 endif if
($kp_setpar<>0) then tau_a=0.005 endif

// Selective 180 for passive spins
exists('pw180_p','parameter'):$e
if $e=0 then create('pw180_p','pulse') setlimit('pw180_p',500000,0,0.0125) pw180_p=18500 endif
if ($kp_setpar<>0) then pw180_p=18500 endif
exists('pwr180_p','parameter'):$e
if $e=0 then create('pwr180_p','real') setlimit('pwr180_p',63,-16,1) pwr180_p=-16 endif if
($kp_setpar<>0) then pwr180_p=-16 endif
exists('shp_p','parameter'):$e
if $e=0 then create('shp_p','string') shp_p='kp_srtACQ_passive' endif if ($kp_setpar<>0) then
shp_p='kp_srtACQ_passive' endif
exists('bw_p','parameter'):$e
if $e=0 then create('bw_p','real') setlimit('bw_p',10000,5,1) bw_p=100
setgroup('bw_p','processing') endif if ($kp_setpar<>0) then bw_p=100 endif
exists('offset','parameter'):$e
if $e=0 then create('offset','frequency') setlimit('offset',100000,-100000,0.1) offset=1000
setgroup('offset','processing') endif if ($kp_setpar<>0) then offset=1000 endif
exists('kp_beta_p','parameter'):$e
if $e=0 then create('kp_beta_p','real') setlimit('kp_beta_p',360,0,0.01) kp_beta_p=180 endif if
($kp_setpar<>0) then kp_beta_p=180 endif
exists('kp_phincr_p','parameter'):$e
if $e=0 then create('kp_phincr_p','real') setlimit('kp_phincr_p',180,-180,0.001) kp_phincr_p=0
endif if ($kp_setpar<>0) then kp_phincr_p=0 endif
exists('kp_stepsize_p','parameter'):$e
if $e=0 then create('kp_stepsize_p','real') setlimit('kp_stepsize_p',100,0.25,0.25)
kp_stepsize_p=10 endif if ($kp_setpar<>0) then kp_stepsize_p=10 endif
exists('kp_wave_p','parameter'):$e
if $e=0 then create('kp_wave_p','string') kp_wave_p='rsnob' endif if ($kp_setpar<>0) then
kp_wave_p='rsnob' endif
exists('tau_p','parameter'):$e
if $e=0 then create('tau_p','delay') setlimit('tau_p',8192,0,0.0000000125) tau_p=0.025 endif if
($kp_setpar<>0) then tau_p=0.025 endif
exists('kp_scyc','parameter'):$e
if $e=0 then create('kp_scyc','string')
setenumeral('kp_scyc',14,'n','m4','t5','t7','m8','t9','m16',
't5m4','t7m4','m32','t9m4','m64','t5m16','t7m16') kp_scyc='t7m4' endif if ($kp_setpar<>0) then
kp_scyc='t7m4' endif

// Basic DDR parameters
exists('rof3','parameter'):$e
if $e=0 then create('rof3','pulse') setlimit('rof3',100,0,0.0125) rof3=10.0 endif if
($kp_setpar<>0) then rof3=10.0 endif
exists('ddrcr','parameter'):$e
if $e=0 then create('ddrcr','integer') setlimit('ddrcr',1000,2,1) ddrcr=2 endif if
($kp_setpar<>0) then ddrcr=2 endif
exists('rof1','parameter'):$e
if $e=0 then create('rof1','pulse') setlimit('rof1',100,0,0.0125) rof1=4.0 endif if
($kp_setpar<>0) then rof1=4.0 endif
exists('rof2','parameter'):$e
```

```
if $e=0 then create('rof2','pulse') setlimit('rof2',100,0,0.0125) rof2=10.0 endif if
($kp_setpar<>0) then rof2=10.0 endif
exists('alfa','parameter'):$e
if $e=0 then create('alfa','pulse') setlimit('alfa',1000,0,0.4) alfa=10.0 endif if
($kp_setpar<>0) then alfa=10.0 endif
exists('ddrtc','parameter'):$e
if $e=0 then create('ddrtc','pulse') setlimit('ddrtc',1000,0,0.4) ddrtc=20.0 endif if
($kp_setpar<>0) then ddrtc=20.0 endif
exists('ddrpm','parameter'):$e
if $e=0 then create('ddrpm','string') ddrpm='e' endif if ($kp_setpar<>0) then ddrpm='e' endif

// Setup DDR timing
if ($kp_setpar<>0) then setrc endif
setlimit('np',524288,64,2)

// String parameters
exists('sspul','parameter'):$e
if $e=0 then create('sspul','flag') setenumeral('sspul',2,'n','y') sspul='y' endif if
($kp_setpar<>0) then sspul='y' endif
exists('PFGflg','parameter'):$e
if $e=0 then create('PFGflg','flag') setenumeral('PFGflg',2,'n','y') PFGflg='y' endif if
($kp_setpar<>0) then PFGflg='y' endif
exists('lkgate_flg','parameter'):$e
if $e=0 then create('lkgate_flg','string') setenumeral('lkgate_flg',3,'n','y','k')
lkgate_flg='k' endif if ($kp_setpar<>0) then lkgate_flg='k' endif
exists('kp_auto','parameter'):$e
if $e=0 then create('kp_auto','flag') setenumeral('kp_auto',2,'n','y') kp_auto='y' endif if
($kp_setpar<>0) then kp_auto='y' endif

// PFG parameters
exists('hsgt','parameter'):$e
if $e=0 then create('hsgt','delay') setlimit('hsgt',0.02,0,0.0000000125) hsgt=0.002 endif if
($kp_setpar<>0) then hsgt=0.002 endif
exists('hsglvl','parameter'):$e
if $e=0 then create('hsglvl','real') setlimit('hsglvl',32768,-32767,1) hsglvl=11177 endif if
($kp_setpar<>0) then hsglvl=11177 endif
exists('gstab','parameter'):$e
if $e=0 then create('gstab','delay') setlimit('gstab',0.1,0,0.0000000125) gstab=0.0005 endif if
($kp_setpar<>0) then gstab=0.0005 endif
exists('gt1','parameter'):$e
if $e=0 then create('gt1','delay') setlimit('gt1',0.02,0,0.0000000125) gt1=0.001 endif if
($kp_setpar<>0) then gt1=0.001 endif
exists('gzlvl1','parameter'):$e
if $e=0 then create('gzlvl1','real') setlimit('gzlvl1',32768,-32767,1) gzlvl1=7864 endif if
($kp_setpar<>0) then gzlvl1=7864 endif
exists('gt2','parameter'):$e
if $e=0 then create('gt2','delay') setlimit('gt2',0.02,0,0.0000000125) gt2=0.001 endif if
($kp_setpar<>0) then gt2=0.001 endif
exists('gzlvl2','parameter'):$e
if $e=0 then create('gzlvl2','real') setlimit('gzlvl2',32768,-32767,1) gzlvl2=10158 endif if
($kp_setpar<>0) then gzlvl2=10158 endif
exists('gzlvl7','parameter'):$e
if $e=0 then create('gzlvl7','real') setlimit('gzlvl7',8192,-8192,1) gzlvl7=320 endif if
($kp_setpar<>0) then gzlvl7=320 endif
exists('kp_pfgtc','parameter'):$e
if $e=0 then create('kp_pfgtc','pulse') setlimit('kp_pfgtc',1000,0,0.0125) kp_pfgtc=6.0 endif
if ($kp_setpar<>0) then kp_pfgtc=6.0 endif
exists('kp_gmult','parameter'):$e
if $e=0 then create('kp_gmult','real') setlimit('kp_gmult',100,-100,0) kp_gmult=10 endif if
($kp_setpar<>0) then kp_gmult=10 endif
if $e=0 then create('ACQ_gt1','delay') setlimit('ACQ_gt1',0.005,0,0.0000000125) ACQ_gt1=0.0005
endif if ($kp_setpar<>0) then ACQ_gt1=0.0005 endif
exists('ACQ_gzlvl1','parameter'):$e
if $e=0 then create('ACQ_gzlvl1','real') setlimit('ACQ_gzlvl1',32768,-32767,1) ACQ_gzlvl1=3079
endif if ($kp_setpar<>0) then ACQ_gzlvl1=3079 endif
exists('ACQ_gzlvl2','parameter'):$e
```

```
if $e=0 then create('ACQ_gzlvl2','real') setlimit('ACQ_gzlvl2',32768,-32767,1) ACQ_gzlvl2=4139
endif if ($kp_setpar<>0) then ACQ_gzlvl2=4139 endif
exists('ACQ_gzlvl3','parameter'):$e
if $e=0 then create('ACQ_gzlvl3','real') setlimit('ACQ_gzlvl3',32768,-32767,1) ACQ_gzlvl3=3547
endif if ($kp_setpar<>0) then ACQ_gzlvl3=3547 endif
exists('ACQ_gzlvl4','parameter'):$e
if $e=0 then create('ACQ_gzlvl4','real') setlimit('ACQ_gzlvl4',32768,-32767,1) ACQ_gzlvl4=4733
endif if ($kp_setpar<>0) then ACQ_gzlvl4=4733 endif
exists('ACQ_gstab','parameter'):$e
if $e=0 then create('ACQ_gstab','delay') setlimit('ACQ_gstab',0.1,0,0.0000000125)
ACQ_gstab=0.0001 endif if ($kp_setpar<>0) then ACQ_gstab=0.0001 endif

// Chunking parameters
exists('droppts1','parameter'):$e
if $e=0 then create('droppts1','real') setlimit('droppts1',16384,0,1) droppts1=2 endif if
($kp_setpar<>0) then droppts1=2 endif
exists('droppts2','parameter'):$e
if $e=0 then create('droppts2','real') setlimit('droppts2',16384,0,1) droppts2=2 endif if
($kp_setpar<>0) then droppts2=2 endif
exists('npoints','parameter'):$e
if $e=0 then create('npoints','real') setlimit('npoints',524288,0,1) npoints=256 endif if
($kp_setpar<>0) then npoints=256 endif

// BIP parameters
exists('BIPflg','parameter'):$e
if $e=0 then create('BIPflg','flag') setenumeral('BIPflg',2,'n','y') BIPflg='n' endif if
($kp_setpar<>0) then BIPflg='n' endif
exists('pw_BIP','parameter'):$e
if $e=0 then create('pw_BIP','pulse') pw_BIP=125 endif if ($kp_setpar<>0) then pw_BIP=125 endif
exists('shp_BIP','parameter'):$e
if $e=0 then create('shp_BIP','string') shp_BIP='hf_HBIP' endif if ($kp_setpar<>0) then
shp_BIP='hf_HBIP' endif
exists('pwr_BIP','parameter'):$e
if $e=0 then create('pwr_BIP','real') setlimit('pwr_BIP',63,-16,1) pwr_BIP=-16 endif if
($kp_setpar<>0) then pwr_BIP=-16 endif /*power of pulse*/

// Phase cycling parameter
exists('kpph','parameter'):$e
if $e=0 then create('kpph','integer') setlimit('kpph',64,0,1) kpph=0 endif if ($kp_setpar<>0)
then kpph=0 endif

// 2D experiment parameters
exists('sw1','parameter'):$e
if $e=0 then par2D sw1=50 phase=0 ni=2 endif

// Set up the basics for any EXQUISITE experiment
UoM_setup_EXQUISITE

if ($kp_setpar<>0) then
 wexp = ''
 np = (npoints+droppts1+droppts2)*2*16
 kp_makePS7(3)
endif

dps
```

S12.1.4. UoM_srt_ps_calcs

```
/* UoM_srt_ps_calcs ---------------------------------------------------------

 Macro to calculate ni, np and tau_p values for semi-real-time acquisition
 mode pure shift NMR experiments.

 Howard M. Foster
 The University of Manchester
 Manchester NMR Methodology Group
 v1.0 04/08/2023
--------------------------------------------------------------------------*/

// Let ni be set to 1 for calculations
ni = 1
$ni = ni

// Check that sw1 is an integer submultiple of sw
if (trunc(sw/sw1) <> (sw/sw1)) then
  write('line3','sw1 must be an integer submultiple of sw')
  abort
endif

// Set npoints to acquire a full pure shift FID
npoints = sw/sw1
write('line3','npoints calculated as %.0f',npoints)

// Determine optimal ni value
$pas_dur = (2.0*ACQ_gt1 + 2.0*ACQ_gstab + 2.0*rof1/1e6 + pw180_p/1e6)
$acq_dur = (npoints + droppts1 + droppts2)/sw
$ni = ($acq_dur + $pas_dur) / (npoints/sw)
$ni = trunc($ni) + 1
if (ni < $ni) then
  ni = $ni
  write('line3','ni calculated as %.0f',ni)
endif

// Determine tau_p
tau_p = ($ni/sw1) - $acq_dur
//tau_p = ($ni/sw1) - $acq_dur + droppts2/sw
write('line3','tau_p calculated as %.6f',tau_p)

// Check np to be acquired (this should include droppts acquired)
$np_ratio = np / (2*(npoints + droppts1 + droppts2))

if (trunc($np_ratio) <> $np_ratio) then
  write('line3','The supplied value of np is not an integer multiple of (npoints + droppts1 +
droppts2). Recalculating...')

  if trunc($np_ratio + 0.5) > trunc($np_ratio) then
    np = trunc($np_ratio + 0.5) * 2*(npoints + droppts1 + droppts2)
  else
    np = trunc($np_ratio) * 2*(npoints + droppts1 + droppts2)
  endif

  write('line3','np calculated as %.0f',np)
endif
```

S12.1.5. go_UoM_EXQUISITE_if

```
/* go_UoM_EXQUISITE_if -----------------------------------------------------

 Go macro for an EXQUISITE pure shift NMR experiment in interferogram
 acquisition mode. Generates RF pulses.

 Howard M. Foster
 The University of Manchester
 Manchester NMR Methodology Group
 v1.0 31/01/2024
--------------------------------------------------------------------------*/

if (kp_auto = 'y') then
  UoM_makePS9(1)
  if (BIPflg = 'y') then
    UoM_bip125('hf_HBIP',tpwr,ref_pw90):shp_BIP,pwr_BIP,pw_BIP
  endif
endif
```

S12.1.6. go_UoM_EXQUISITE_srt

```
/* go_UoM_EXQUISITE_srt ----------------------------------------------------

 Go macro for an EXQUISITE pure shift NMR experiment in semi-real-time
 acquisition mode. Generates RF pulses and determines the values of ni, np
 and tau_p.

 Howard M. Foster
 The University of Manchester
 Manchester NMR Methodology Group
 v1.0 31/01/2024
--------------------------------------------------------------------------*/

// Generate RF pulses
if (kp_auto = 'y') then
  kp_makePS7(3)
endif

// Calculate ni, np and tau_p values
UoM_srt_ps_calcs
```

S12.2. Processing macros

S12.2.1. UoM_proc_1d_if_array

```
/* UoM_proc_1d_if_array -----------------------------------------------------

 Macro to process an array of EXQUISITE pure shift NMR interferogram
 acquisition mode datasets. Produces an array of 1D pure shift NMR spectra.

 Derived from the "UoM_proc_1d_if" macro.

 Howard M. Foster
 The University of Manchester
 Manchester NMR Methodology Group
 v1.0 23/04/2022
--------------------------------------------------------------------------*/

jexp:$exp,$expname

$nfid=ni

if (ni<2) then
banner('Data has already been processed..')
abort
endif

cptmp('pureshift')

if (lsfid>0) then
  $droppts=lsfid+1
else
  $droppts=1
ENDIF
exists('droppts','parameter'):$ex
IF $ex>0 then
  $droppts=droppts
ENDIF

$npoint=trunc((sw/sw1)+0.5)
$tmpfile=userdir+'/'+$expname+'/homodec_writefid'
$tmpfile3=userdir+'/'+$expname+'/homodec_writefid_drop'
$tmpfile2=userdir+'/'+$expname+'/homodec_fid'
$tmpfile4=userdir+'/'+$expname+'/homodec_single_fid'
beepoff

$chunk1=$npoint

$imag=0.0
$real=0.0

exists($tmpfile,'file'):$ex1
IF $ex1>0 then
    shell('rm',$tmpfile)
ENDIF

$kp_droppts_str=''
$kp_npoints_str=''
$kp_np_str=''
format($droppts+1,0,0):$kp_droppts_str
format(np/2,0,0):$kp_np_str
format($npoint,0,0):$kp_npoints_str

exists($tmpfile2,'file'):$ex1
IF $ex1>0 then
    shell('rm',$tmpfile2)
```

```
ENDIF


$totalfids = arraydim
$arraysize = $totalfids / ni



/*$i=1
$i_str=''
format($i,0,0):$i_str*/

$k = 1
$k_str=''
format($k,0,0):$k_str

REPEAT

  $i = $k

  REPEAT
    writefid($tmpfile,$i)
    shell('sed -n '+$kp_droppts_str+','+$kp_np_str+'p '+$tmpfile+' > '+ $tmpfile3)
    shell('sed -n 1,'+$kp_npoints_str+'p '+$tmpfile3+' >> '+ $tmpfile2)
    exists($tmpfile,'file'):$ex1
    IF $ex1>0 then
      shell('rm',$tmpfile)
    ENDIF
    exists($tmpfile3,'file'):$ex1
    IF $ex1>0 then
      shell('rm',$tmpfile3)
    ENDIF

    $i = $i + $arraysize
  UNTIL ($i > $totalfids)

  $k = $k + 1
UNTIL ($k > $arraysize)


rm(curexp+'/acqfil/fid')
shell('sleep 1')

$j = 1
$j_str=''
format($j,0,0):$j_str
$start_index_str=''
$end_index_str=''

REPEAT

  $start_index = (($j - 1) * $npoint * ni) + 1
  $end_index = $j * $npoint * ni
  format($start_index,0,0):$start_index_str
  format($end_index,0,0):$end_index_str

  shell('sed -n '+$start_index_str+','+$end_index_str+'p '+$tmpfile2+' > '+ $tmpfile4)
  makefid($tmpfile4,$j,'float')
  exists($tmpfile4,'file'):$ex1
  IF $ex1>0 then
      shell('rm',$tmpfile4)
  ENDIF

  $j = $j + 1
UNTIL ($j > $arraysize)

setvalue('np',2.0*($npoint*($nfid-1)+$chunk1))
```

```
setvalue('fn',np)
setvalue('at', 0.5*np/sw)
groupcopy('current','processed','acquisition')

lb='n' gf=at/2 lsfid=0
fn=16*np ni=0
UoM_nowt gf=at/2
groupcopy('current','processed','acquisition')
wft('nodc') aph0 vsadj
```

S12.2.1. UoM_proc_1d_srt_array

```
/* UoM_proc_1d_srt_array --------------------------------------------------

 Macro to process an array of EXQUISITE pure shift NMR semi-real-time
 acquisition mode datasets. Produces an array of 1D pure shift NMR spectra.

 Derived from the "kp_srtACQ31_proc_v6" macro written by Peter Kiraly.

 Howard M. Foster
 The University of Manchester
 Manchester NMR Methodology Group
 v1.0 29/05/2023
---------------------------------------------------------------------------*/

jexp:$exp,$expname

$nfid = arraydim
$numreps = arraydim / ni
write('line3','numreps is %f',$numreps)

// Check whether data has already been processed
if (ni<2) then
banner('Data has already been processed...')
abort
endif

// Set drop points
if (lsfid > 0) then
  $droppts1 = lsfid+1
else
  $droppts1 = 1
endif

exists('droppts1','parameter'):$e
if $e > 0 then
  $droppts1 = droppts1
endif

exists('droppts2','parameter'):$e
if $e > 0 then
  $droppts2 = droppts2
endif

// Get npoints
$npoints=trunc((sw/sw1)+0.5)
write('line3','npoints is %f',$npoints)

// Get nchunks
$nchunks = (0.5*(np))/($droppts1 + $droppts2 + $npoints)
write('line3','nchunks is %f',$nchunks)

// Get np_max
$np_max = ni*$nchunks*$npoints
write('line3','np_max is %f',$np_max)


// Create temporary directory for pure shift data
exists(curexp+'/subexp/pureshift','file'):$e
if $e = 1 then
  shell('rm -r '+curexp+'/subexp/pureshift')
endif
cptmp('pureshift')

// Clean up temporary files for pure shift data if necessary
$tmpfile=userdir+'/'+$expname+'/homodec_writefid'
$tmpfile3=userdir+'/'+$expname+'/homodec_writefid_drop'
```

```
$tmpfile2=userdir+'/'+$expname+'/homodec_fid'
$tmpfile4=userdir+'/'+$expname+'/homodec_all_reps'
beepoff

exists($tmpfile,'file'):$e
if $e > 0 then
  shell('rm',$tmpfile)
endif

exists($tmpfile3,'file'):$e
if $e > 0 then
  shell('rm',$tmpfile3)
endif

exists($tmpfile2,'file'):$e
if $e > 0 then
  shell('rm',$tmpfile2)
endif


// Initialise strings used in processing
$kp_npoints_str=''
format($npoints,0,0):$kp_npoints_str

// Loop through each increment, write increment to tmpfile
$k = 1
$i = 1
$x = 1
$last = 0
$first_str = ''
$last_str = ''

REPEAT

  write('line3','Starting loop through iteration %f',$x)

  // Reset $k
  $k = 1

  // Loop through each chunk
  REPEAT
    if $k = 1 then
      $first = $droppts1 +1
    else
      $first = $last + ($droppts1 + $droppts2) +1
    endif
    $last = $first-1 + $npoints

    format($first,0,0):$first_str
    format($last,0,0):$last_str

    // Reset $i
    $i = $x

    // Loop through each increment
    while ($i < $nfid+1) do

      //write('line3','Starting loop through fid %f',$i)

      writefid($tmpfile,$i)
      shell('sed -n '+$first_str+','+$last_str+'p '+$tmpfile+' > '+ $tmpfile3)
      shell('sed -n 1,'+$kp_npoints_str+'p '+$tmpfile3+' >> '+ $tmpfile2)
      exists($tmpfile,'file'):$ex1
      IF $ex1>0 then
        shell('rm',$tmpfile)
      ENDIF
      exists($tmpfile3,'file'):$ex1
```

```
      IF $ex1>0 then
        shell('rm',$tmpfile3)
      ENDIF

      $i = $i + $numreps

      if $i>200 then
        return
      endif

    endwhile


    $k = $k + 1

  UNTIL ($k > $nchunks)

  $x = $x +1

UNTIL ($x > $numreps)

write('line3','Completed loops through each increment')

// Remove current FID in experiment
rm(curexp+'/acqfil/fid')
shell('sleep 1')

// Write new FID
$x = 1
$first = 1
$last = $np_max

REPEAT

  exists($tmpfile4,'file'):$e
  if $e > 0 then
    shell('rm',$tmpfile4)
  endif

  if $x > 1 then
    $first = $last +1
    $last = $last + $np_max
  endif

  format($first,0,0):$first_str
  format($last,0,0):$last_str

  // Write new FID
  shell('sed -n '+$first_str+','+$last_str+'p '+$tmpfile2+' > '+ $tmpfile4)
  makefid($tmpfile4,$x,'float')

  $x = $x+1

UNTIL ($x > $numreps)

setvalue('np',$np_max*2.0)
setvalue('fn',$np_max*4.0)
setvalue('at',$np_max/sw)
groupcopy('current','processed','acquisition')

lb='n' gf=at/2 lsfid=0
ni = 0
UoM_nowt gf=at/2
groupcopy('current','processed','acquisition')
wft('nodc') aph0 vsadj
```
